# Hindcast‐validated species distribution models reveal future vulnerabilities of mangroves and salt marsh species

**DOI:** 10.1002/ece3.9252

**Published:** 2022-09-19

**Authors:** Richard G. J. Hodel, Douglas E. Soltis, Pamela S. Soltis

**Affiliations:** ^1^ Department of Botany National Museum of Natural History Washington DC USA; ^2^ Department of Biology University of Florida Gainesville Florida USA; ^3^ Florida Museum of Natural History University of Florida Gainesville Florida USA; ^4^ The Genetics Institute, University of Florida Gainesville Florida USA; ^5^ The Biodiversity Institute University of Florida Gainesville Florida USA

**Keywords:** coastal species, ecological niche modeling, ground truth, hindcast validation, model validation, species distribution modeling

## Abstract

Rapid climate change is threatening biodiversity via habitat loss, range shifts, increases in invasive species, novel species interactions, and other unforeseen changes. Coastal and estuarine species are especially vulnerable to the impacts of climate change due to sea level rise and may be severely impacted in the next several decades. Species distribution modeling can project the potential future distributions of species under scenarios of climate change using bioclimatic data and georeferenced occurrence data. However, models projecting suitable habitat into the future are impossible to ground truth. One solution is to develop species distribution models for the present and project them to periods in the recent past where distributions are known to test model performance before making projections into the future. Here, we develop models using abiotic environmental variables to quantify the current suitable habitat available to eight Neotropical coastal species: four mangrove species and four salt marsh species. Using a novel model validation approach that leverages newly available monthly climatic data from 1960 to 2018, we project these niche models into two time periods in the recent past (i.e., within the past half century) when either mangrove or salt marsh dominance was documented via other data sources. Models were hindcast‐validated and then used to project the suitable habitat of all species at four time periods in the future under a model of climate change. For all future time periods, the projected suitable habitat of mangrove species decreased, and suitable habitat declined more severely in salt marsh species.

## Graphical abstract

Species distribution modeling can project the potential future distributions of species under scenarios of climate change using bioclimatic data and georeferenced occurrence data. However, models projecting suitable habitat into the future are impossible to ground truth. Here, we overcome this limitation by developing species distribution models for the present and projecting them to periods in the recent past where distributions are known to test model performance before making projections into the future.

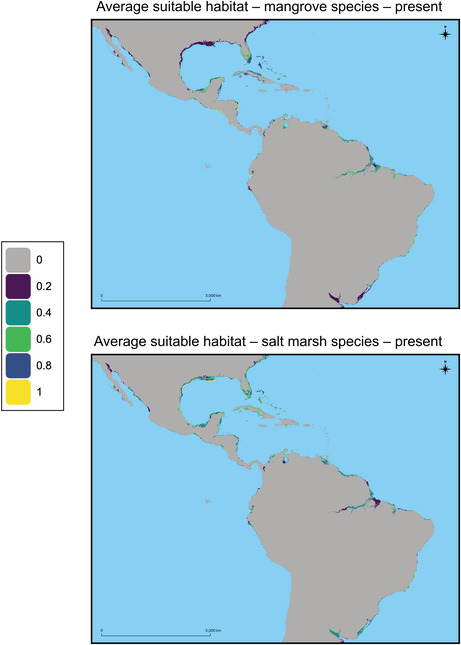

## 1 INTRODUCTION

Climate change is rapidly impacting biodiversity, and research over the past several decades has provided many key insights regarding how diverse species might respond to climate change in the near future (e.g., Franks et al., 2007; Parmesan et al., 2015; Sinervo et al., 2010; Tingley et al., 2012; Visser et al., 1998). Recent studies have demonstrated that climate change may have dramatic effects on organisms through population decline (Jenouvrier et al., 2009), extinction (e.g., Brook et al., 2008; Cahill et al., 2013), and shifts in geographic distributions (e.g., Kearney et al., 2009; Lafferty, 2009). In contrast, other species are predicted to have significant range expansions following climate change (e.g., Cudmore et al., 2010; Soltis & Soltis, 2016). Whereas many species are vulnerable to the effects of climate change, there is mounting evidence that species with exclusively coastal distributions are especially at risk and have already undergone significant distributional shifts (Bowman et al., 2010; Ellison, 1993; Everitt et al., 2010; Feagin et al., 2005; Gilman et al., 2007; Howari et al., 2009; López‐Medellín et al., 2011; Shearman, 2010; Williamson et al., 2011). Climate change is projected to have a dramatic impact on coastal plant species in the near future (i.e., remainder of this century), but not all species and coastal plant communities will be affected in the same way (Bowman et al., 2010; Ellison, 1993; Everitt et al., 2010; Gilman et al., 2007; Shearman, 2010; Williamson et al., 2011 ). Some species will be severely threatened by habitat loss and are predicted to experience a dramatic decrease in distribution (e.g., Feagin et al., 2005), whereas others may undergo shifts in their ranges (e.g., López‐Medellín et al., 2011).

A critical threat to coastal communities is their hypothesized inability to move inland rapidly enough to keep pace with rapid changes in sea level rise (SLR) (Kirwan & Megonigal, 2013). SLR directly impacts species inhabiting coastal zones and leads to habitat change and eventual habitat loss for many taxa, including migratory shore birds (Iwamura et al., 2013), salt marsh grasses (Adam, 2002), and gastropods (McFarlin et al., 2015). Mendoza‐González et al. (2013) found striking impacts on coastal sand dune taxa in the Yucatán Peninsula of Mexico—they projected up to an 85% reduction in suitable habitat for dune plant species by the end of the century. In Panama and Costa Rica, 40% of mangrove species are considered threatened (Polidoro et al., 2010). Many studies have been conducted on relatively small spatial scales (e.g., in several neighboring estuaries) and have provided vital insights into how climate change is currently affecting, and will impact, the species in local coastal study sites (e.g., Stevens et al., 2006). While localized studies are crucial, they are also time‐consuming, and rapid climate change means that we do not have the luxury of protracted studies to identify coastal areas where inhabitant species are vulnerable to climate change. A modeling approach can rapidly project the future suitable habitat for multiple coastal species over a wide geographic area. Such models, in concert with local studies, can provide a useful projection of climate change impact on coastal species.

Species distribution modeling (SDM) is a powerful tool for projecting where suitable habitat may exist in the future by using layers of environmental data (e.g., mean annual temperature, mean annual precipitation) and species occurrence data (e.g., georeferenced records in natural history collections). SDM has been used to predict species range shifts, invasions, and novel species interactions in response to climate change (e.g., Gilman et al., 2010; Urban et al., 2012). SDM approaches can identify locations with suitable habitat for species, by using information where species currently live or have lived in the recent past. By quantifying environmental variables in discrete, predefined areas (e.g., 1 km^2^ patches across a landscape), researchers can identify abiotic environmental factors that make some areas more favorable than others for the survival of species of interest. Next, using models of past (e.g., climate projections for the mid‐Holocene) or future (e.g., IPCC projections for future time periods) climate change, SDM analyses can be used to identify areas that had or will likely have suitable habitat for species of interest based on projected values of environmental variables in different time periods (e.g., Hodel et al., 2021).

SDM analyses use occurrence data and environmental data to predict the geographic space where abiotic conditions allow existence of a population or species. SDM analyses typically only take into account abiotic environmental factors to predict suitable habitat, and biotic data are therefore not incorporated into the model. Despite a lack of biotic data, SDM still provides critical insights about a species' distribution on large spatial scales; it would be virtually impossible to collect biotic data on such a large scale. Another drawback of projecting suitable habitat into the future is the impossibility of ground‐truthing modeling results. We propose a novel solution to this conundrum: constructing SDMs for the present and hindcasting them into past time periods when species presence was documented to test model accuracy. To our knowledge, no other study has implemented this approach to validate SDMs before projecting into the future. Ground‐truthing strategies were used in at least two other studies; Wogan (2016) used historical climate and occurrence data in a niche modeling framework to test the spatial transferability of SDMs, and Varma and Bebber (2019) used hindcast climate data and banana yield data to infer the impact of climate on yield in the past and projected into the future.

A recent analysis of historical images and topographic sheets detected shifts toward mangrove dominance in a mangrove‐salt marsh ecotone after decades of oscillating dominance (Cavanaugh et al., 2019), and investigations using satellite imagery documented poleward shifts in mangrove distributions in North America over just the last few decades (Cavanaugh et al., 2014). On a more local scale, the ecotone in northeastern Florida that defines a transition from mangroves to salt marsh species has alternated between periods of occupancy by mangroves and salt marsh species on fine temporal scales (i.e., less than a decade; Cavanaugh et al., 2019). Extreme low‐temperature events are often attributed to be a cause of mangrove dieback, which would facilitate salt marsh dominance (Duke et al., 2017; Saintilan et al., 2014). Subsequent periods lacking extreme cold events may then promote mangrove invasion of salt marshes (Cavanaugh et al., 2014). The frequency of extreme cold events creates a dynamic mosaic of different ecosystem types on small spatial scales (Yando et al., 2016). Salt marshes are also vulnerable to the environmental effects associated with climate change, such as drought events (Alber et al., 2008). Coarse‐resolution modeling studies in the southeastern United States identified areas where salt marshes are at risk for mangrove invasion in the future (areas in Florida, Louisiana, and Texas; Osland et al., 2013). Using an SDM approach to examine the dynamics of mangrove communities under scenarios of climate change and SLR, Record et al. (2013) identified poleward shifts in some mangrove communities. Coastal ecosystems, notably salt marshes and mangroves, require further investigation using SDM at both a fine spatial resolution (i.e., using <5 km^2^ grid cells) and on a large scale (i.e., the Americas). In the present study, we leverage known periods of dominance (Cavanaugh et al., 2019) to validate SDMs that model present suitable habitat and hindcast into time periods where either mangrove species or salt marsh species dominated to determine if the model predicts the correct trend in each group.

For SDM analyses, the present is typically considered any time after 1950 (Hijmans et al., 2005). However, with the release of WorldClim 2.1, bioclimatic variables are available for every month between 1960 and 2018, which enables SDM analyses at much finer temporal scales. We constructed SDMs for the present (which we define as 2013–2018) to infer locations of suitable habitat for eight species in the Neotropics. We then made projections into two time periods in the past that correspond to documented periods of either mangrove or salt marsh dominance (Cavanaugh et al., 2019). Specifically, our objectives were to (1) construct SDMs to infer suitable habitat for four mangrove species and four salt marsh species in the present; (2) validate SDMs by projecting the model backward to time periods in the recent past of known dominance by either mangroves (early 2000s) or salt marsh species (late 1980s) by using change in suitable habitat; (3) use projections of climate change to infer the putative suitable habitat available to these species in the future over three 20‐year periods (2021–2040, 2041–2060, and 2061–2080); and (4) compare current and future habitat suitability for mangrove and salt marsh species by quantifying changes in the geographic extent of species' suitable habitat from present to future.

## 2 METHODS

### 2.1 Data acquisition

We obtained specimen‐based occurrence data for each species from iDigBio (Integrated Digitized Biocollections; idigbio.org) and GBIF (Global Biodiversity Information Facility; gbif.org) and supplemented these data with locality data from personal collections for three mangrove species (*Avicennia germinans*, *Laguncularia racemosa*, *Rhizophora mangle*). Four of the species included in the analysis are mangroves (*Avicennia germinans*, black mangrove; *Laguncularia racemosa*, white mangrove; and *Rhizophora mangle*, red mangrove) or mangrove‐associated species (*Conocarpus erectus*, buttonwood). For simplicity, these four species will hereafter be collectively referred to as “mangroves,” even though *Conocarpus erecuts* is not considered a true mangrove (Tomlinson, 2016). We also selected four salt marsh species (*Batis maritima*, turtleweed; *Sesuvium portulacastrum*, sea purslane; *Spartina alterniflora*, smooth cordgrass; and *Sporobolus virginicus*, seashore dropseed) for analyses. These four species were selected because they occur in close proximity to one another—indicating the presence of salt marsh habitat—and because of their broad and exclusively coastal distributions in the Neotropics. We used SDM to investigate changes in suitable habitat for all eight species. The raw data were cleaned using standard approaches and R scripts (e.g., Marchant et al., 2017); duplicates and incorrect data (e.g., latitude and longitude of 0) were removed from the data set (all scripts used in this paper were deposited in GitHub [github.com/richiehodel/coastal_ENM]), and all cleaned occurrence data, layers, and models were deposited in Dryad (https://doi.org/10.5061/dryad.08kprr55b). We included species that had exclusively coastal or estuarine distributions, and only species with at least 50 occurrence points (after cleaning) were used in the analyses. Given the complexities of the modeling approach, we focused on the Neotropics as opposed to a global analysis; only mangrove and salt marsh species with native ranges in the Americas were used (i.e., cosmopolitan species were excluded). Certain species that inhabit salt marshes, but that have extensive inland distributions, including freshwater wetlands, were excluded (e.g., *Distichlis spicata*).

We acquired bioclimatic environmental layers from Worldclim 2.1 (worldclim.org; Fick & Hijmans, 2017) for multiple time periods. The bioclimatic layers, which contain temperature and precipitation data for every continent except Antarctica, have been used extensively and successfully in SDM studies (Booth, 2018). In Worldclim 2.1, annual precipitation, maximum temperature, and minimum temperature data are available for every month from 1960 to 2018 at 2.5 arc minute resolution; these three variables can be used to calculate values of all 19 bioclimatic variables (Fick & Hijmans, 2017; Harris et al., 2014; Hijmans et al., 2017). We considered the present to be 2013–2018, the 1980s salt marsh dominance period to be 1984–1989, and the early 2000s mangrove dominance to be 2001–2006. These time periods were selected to capture the optimal amount of either mangrove or salt marsh dominance during each documented oscillation (Cavanaugh et al., 2019), and we selected these windows of time so that the present and past time periods were all 6 years. Although many of the study species may be longer‐lived than each of the time periods (i.e., 6 years), we prioritized using time periods that captured either mangrove or salt marsh dominance. Due to the oscillations of mangrove versus salt marsh dominance, many individual plants were likely exterminated on short time scales. We used all occurrence data to construct an SDM for each species for our defined present time (2013–2018) regardless of when the specimens were collected. It would be ideal to use separate occurrence specimens from each time period to assess SDM performance, but this was not possible with the temporal distribution of georeferenced data points. For each 6‐year time period, we averaged the annual precipitation, maximum temperature, and minimum temperature for each month (e.g., average values of these three variables were calculated across the six January months, six February months, etc., in each time period) and used the resulting 12 monthly averages to calculate the standard 19 bioclimatic variable values using the “biovars” function in the “dismo” R package for each 6‐year time period (Hijmans et al., 2017). The standard 19 bioclimatic variables are not available on a monthly basis because some of them incorporate seasonality and require data for at least 1 year. By using monthly data for annual precipitation, maximum temperature, and minimum temperature variables, all of the 19 bioclimatic variables can be calculated (Hijmans et al., 2017).

**FIGURE 1:**
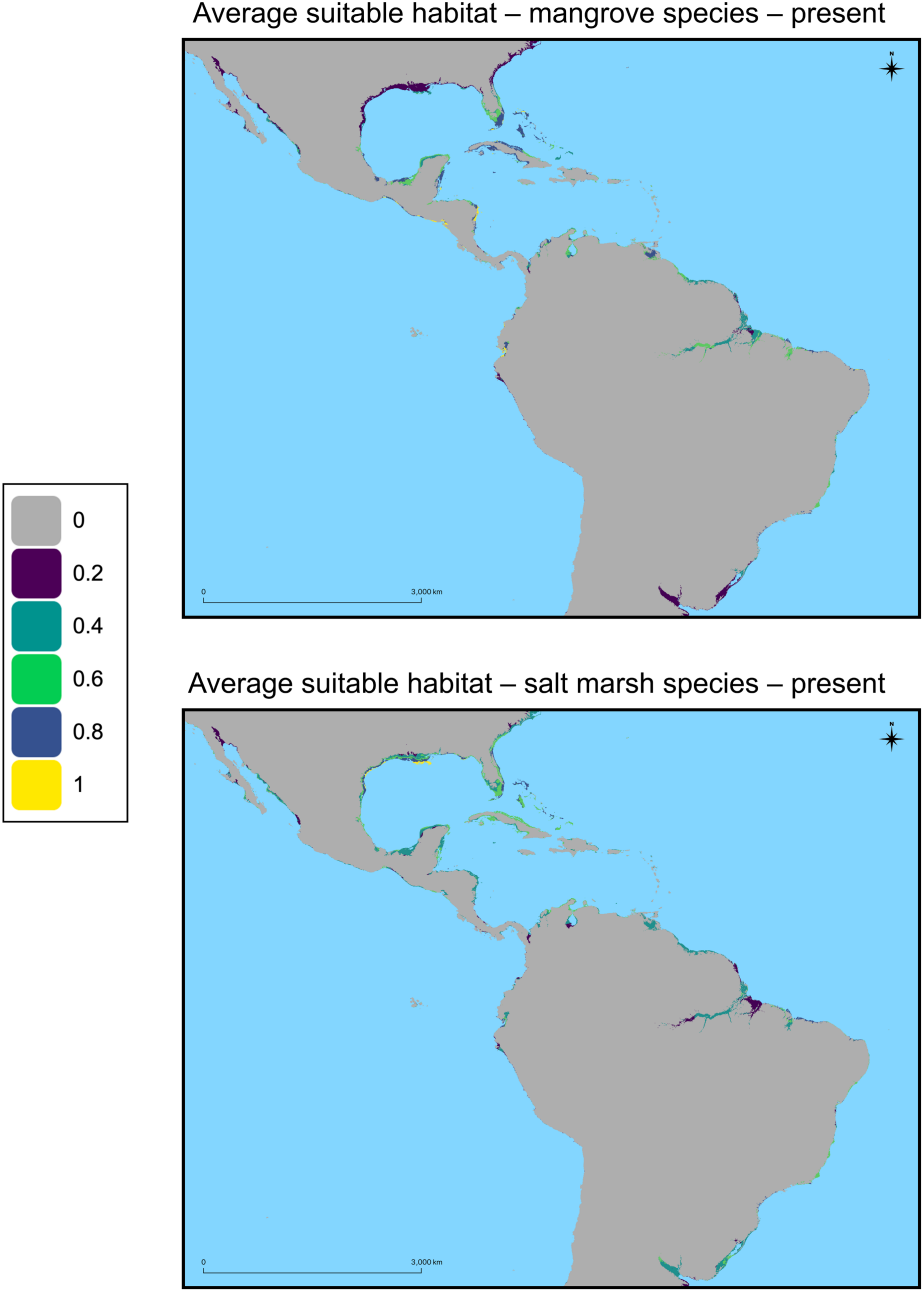
The suitable habitat averaged for the four mangrove species (top) and the four salt marsh species (bottom) in the present (defined as 2013–2018) for the entire geographic study region. For each plot the average suitable habitat is shown to in Table 2.

All layers were then trimmed so that the extent of the study area was between −120 and −32 degrees longitude, and −36 and 36 degrees latitude using custom scripts and the R package “raster” (Hijmans et al., 2015) and exported in ASCII format (Figure 1). This study area was selected because it included subtropical and tropical regions of both the Northern and Southern Hemispheres, captured the ecotone between mangrove and salt marsh species in both Hemispheres, and allowed for an expansion zone as some species may expand their ranges in the future as the climate changes. Regions such as Hawaii, where some Neotropical mangrove species have been introduced, were not included in the study. We used an R script and the R package “raster” (Hijmans et al., 2015) to measure the pairwise correlation of the 19 bioclimatic variables. When variables were correlated with one another (*r* > .7), only one of the layers was retained for subsequent analyses (Dormann et al., 2013). After removing correlated layers, we had a data set of six bioclimatic variables (BIO2, mean diurnal temperature range; BIO5, maximum temperature of warmest month; BIO6, minimum temperature of coldest month; BIO12, annual precipitation; BIO15, precipitation seasonality; BIO18, precipitation of warmest quarter). BIO6 and BIO1 were highly correlated (*r* = .956), and BIO1 (mean annual temperature) was excluded even though it is frequently included in SDM analyses because BIO6 has been identified as an important variable shaping range limits of coastal species (Tomlinson, 2016). All layers were clipped using the “mask” function in the “raster” R package (Hijmans et al., 2015) such that all cells with elevation greater than 10 m were considered “no data” cells. This was done to ensure that the SDM analyses were not trained on inland regions representing areas where these coastal species do not occur.

### 2.2 Species distribution modeling

The occurrence data obtained from digitized herbaria records and the six environmental layers were used as input for the SDM analyses. SDM uses the occurrence data for each species in the present to identify pixels that have suitable habitat for the species of interest based on environmental data. We used the maximum entropy algorithm implemented in MAXENT v3.4.1 (Phillips et al., 2006, 2017) to conduct SDM analyses. The maximum entropy algorithm uses presence data and random background sampling to develop the model, and it has been shown to perform well with presence‐only data (Elith et al., 2006; Wisz et al., 2008). Optimal settings for MAXENT model fit were determined using the “ENMevaluate” function in the ENMeval R package (Muscarella et al., 2014). We investigated regularization multipliers from 0.5 to 4 at intervals of 0.5 and the following features/combinations of features: linear, linear/quadratic, linear/quadratic/hinge, linear/quadratic/hinge/product, linear/quadratic/product/threshold, and linear/quadratic/hinge/product/threshold. The “ENMevaluate” function was run for each species, using the same 10,000 background points, occurrence data for the species of interest, and the “maxnet” algorithm with the “checkerboard2” method. The ΔAICc scores for all models tested for each species were compared to determine the optimal model to be inputted into MAXENT. Other non‐default settings used include fivefold cross‐validation, a minimum training presence threshold, and fading by clamping. Cloglog output was used because it produces an estimate for each pixel between 0 and 1 that represents probability of presence (Phillips et al., 2017).

We assessed each model's prediction ability by using partial receiver operating characteristic (pROC), which measures the ratio of the area under the receiver operating characteristic curve (AUC). AUC ranges from 0 to 1 and measures the model's ability to predict suitable habitat, with 1 indicating perfect discrimination between suitable and unsuitable habitat. The pROC is the ratio of the partial AUC divided by random expectation, and it can range from 0 to 2, with 1 representing random model performance (Escobar et al., 2018). For independent occurrence points, this metric measures the relationship of omission error and proportion of suitable area under conditions of low omission errors (Peterson et al., 2008). Jackknife tests of regularized training gain were used to measure the relative contribution of each bioclimatic variable to the model. Average habitat suitability values for each pixel were modeled for the present, past, and future for each species, and these values were used in downstream analyses. For a given region, the sum of habitat suitability scores was considered the total suitable habitat.

### 2.3 SDM validation and projection into future

We measured each species' suitable habitat and how it was projected to change from the present to the past. First, we defined an area representing the northeastern Florida ecotone used in previous studies to use for hindcast validations: between −82 and −80 degrees longitude, and 28 and 31 degrees latitude (Cavanaugh et al., 2019). For convenience, we hereafter refer to this region as “NE Florida.” We considered the SDM to be properly fit when it accurately inferred the anticipated relative change in suitable habitat between the average mangrove species and the average salt marsh species for all past time periods in the NE Florida validation region. We also used a larger geographic region (between −87 and −79 degrees longitude, and 24 and 31 degrees latitude) to test if the hindcast validations were consistent when a larger region was used; we hereafter refer to this region as “Florida” for simplicity.

Once the SDMs were hindcast‐validated, the same approach was used to infer projected change in suitable habitat for the three time periods in the future. To project future values of environmental variables, we used a widely used and well‐validated climate model—the CNRM‐CM6‐1 model, which is a fully coupled atmosphere–ocean general circulation model developed by Centre National de Recherches Météorologiques (CNRM) for the sixth generation of the IPCC Coupled Model Intercomparison Project 6 (CMIP6), and with the shared socioeconomic pathway 245 (Eyring et al., 2016). This climate model was selected because it is one of 49 used in the most recent IPCC CMIP6, is compatible with the WorldClim 2.1 data used for hindcast analyses (https://worldclim.org/data/cmip6/cmip6climate.html), and the shared socioeconomic pathway was selected because it represents a central part of the range of plausible future pathways. SDMs for the full geographic study region were projected into both the past and future time periods. Future time periods were determined by availability of WorldClim 2.1 data.

## 3 RESULTS

### 3.1 Species distribution modeling

For all mangrove species, the most important bioclimatic variable in terms of model contribution was BIO6 (minimum temperature of coldest month) (Table 1). For all salt marsh species except *Sesuvium portulacastrum*, BIO6 was one of the two most important bioclimatic variables. Additionally, all salt marsh species had BIO12 (annual precipitation) as one of the two most important variables (Table 1). We used between 69 and 449 georeferenced occurrence points per species for SDM (Tables S1 and S2, Figure S1). Model parameters were optimized for each species using ΔAICc scores (Table S3). The pROC scores indicated good model performance across all species (Figure S2).

**TABLE 1:** For each species, the percent contribution of each bioclimatic variable to the species distribution model; the two variables with the highest percent contribution are shown in bold.

Species	*Avicennia germinans*	*Conocarpus erectus*	*Laguncularia racemosa*	*Rhizophora mangle*	*Batis maritima*	*Sesuvium portulacastrum*	*Spartina alterniflora*	*Sporobolus virginicus*
BIO2	9.8	1.6	9.2	4.8	11.9	**19.8**	10.3	13.9
BIO5	2.4	4.9	2.4	1.8	0.2	5.1	1.4	21.5
BIO6	**45.0**	**69.6**	**46.8**	**52.7**	**14.4**	11.7	**41.7**	**24.6**
BIO12	16.8	5.0	**15.4**	15.5	**55.2**	**28.7**	**30.8**	**24.4**
BIO15	**19.8**	5.2	14.3	4.6	9.4	19.1	8.1	8.4
BIO18	6.3	**13.7**	11.9	**20.5**	9.0	15.8	7.8	7.3

*Note*: The first four taxa listed are mangrove species and the last four are salt marsh species. BIO2, mean diurnal temperature range; BIO5, maximum temperature of warmest month; BIO6, minimum temperature of coldest month; BIO12, annual precipitation; BIO15, precipitation seasonality; BIO18, precipitation of warmest quarter.

### 3.2 Model validation

**FIGURE 2:**
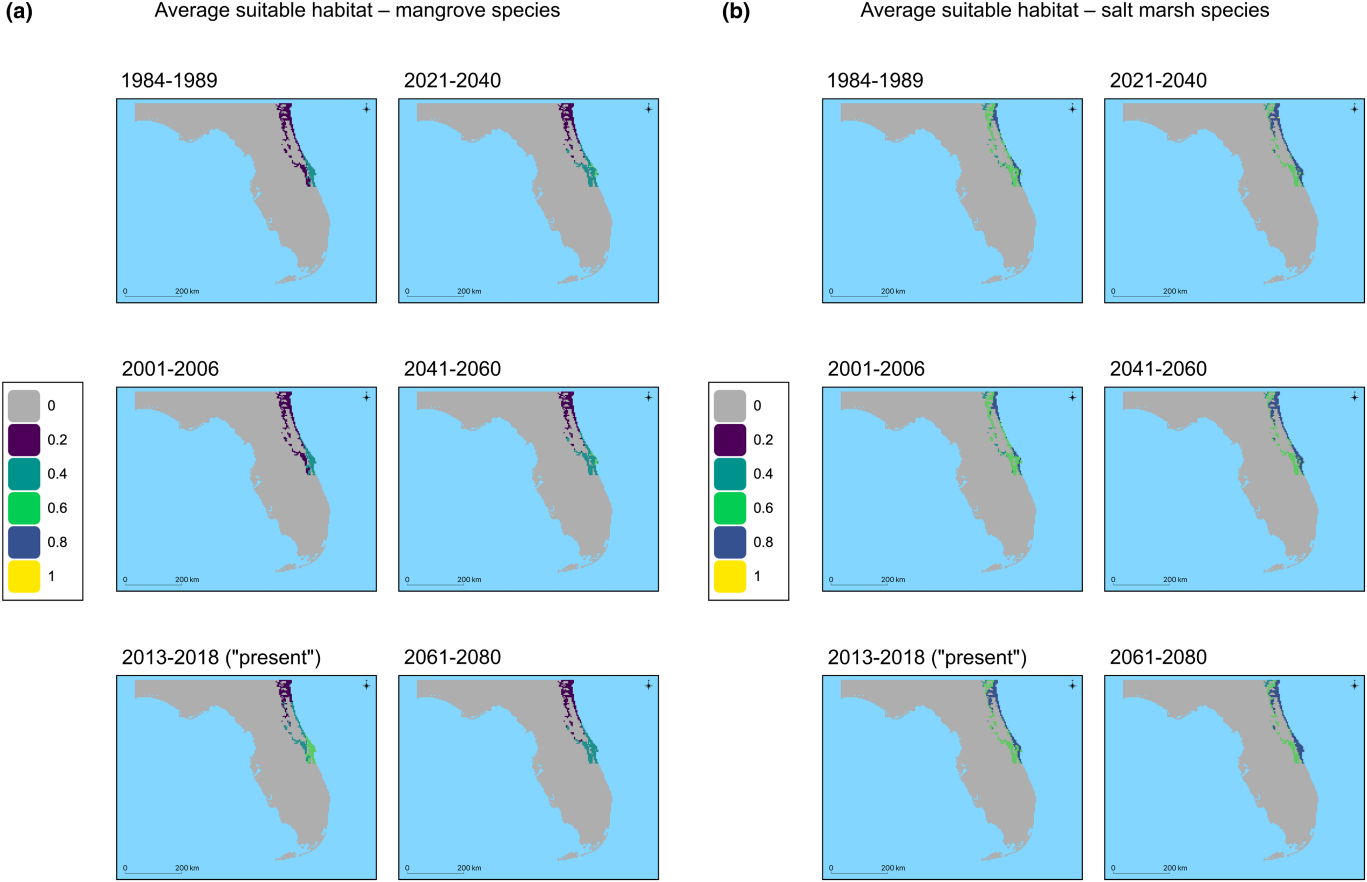
For the NE Florida validation region, the projected suitable habitat is shown for the two past hindcast‐validation time periods, as well as the present, and for three future time periods. The average mangrove suitable habitat is shown in (a) and average salt marsh suitable habitat is shown in (b). For each plot the average suitable habitat is shown in Table 2.

**FIGURE 3:**
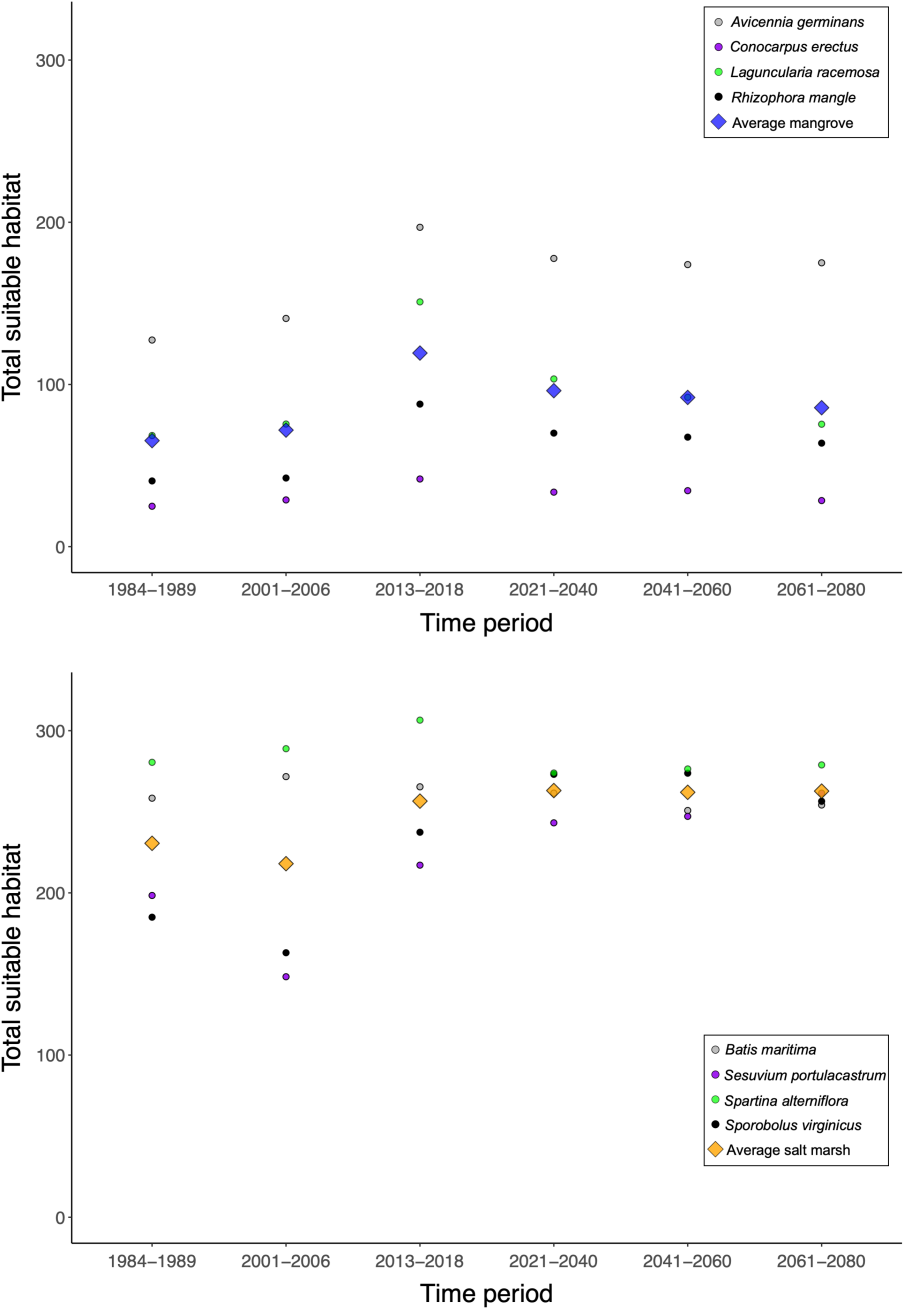
The SDM‐defined suitable habitat for the time periods in the past used to validate the model on the smallest spatial scale (NE Florida), in the present, and projected into the future. The total suitable habitat available for each mangrove species (top) and each salt marsh species (bottom) is shown using colored circles. The average across all four species is shown in colored diamonds.

The SDM results were hindcasted into past time periods when relative mangrove species versus salt marsh species dominance was known on several geographic scales (Figure 2; Figures S3–S5). There were not specific expectations as to whether mangrove species or salt marsh species would exhibit an increase or decrease in suitable habitat relative to the present; rather, we expected that in periods of dominance by one group, there would be a larger increase or smaller decrease in suitable habitat for that group relative to the other group. In the NE Florida validation region, average mangrove suitable habitat was smallest in the period of salt marsh dominance (1984–1989), larger in the period of mangrove dominance (2001–2006), and largest in the present (2013–2018; Table 2, Figure 3). These trends were consistent when each mangrove species was considered separately. Meanwhile, average salt marsh suitable habitat was smallest in the period of mangrove dominance (2001–2006), larger in the period of salt marsh dominance (1984–1989), and largest in the present (2013–2018; Table 2, Figure 3). However, this trend was only matched by two of the individual salt marsh species (*Sesuvium portulacastrum* and *Sporobolus virginicus*). In the larger Florida validation region, all results had the same qualitative trends as in the NE Florida hindcast validation region (Table 2; Figure S6). Considering the ratio of mangrove:salt marsh suitable habitat also demonstrated the utility of hindcast validation (Table 3). In NE Florida, the ratio of mangrove:salt marsh suitable habitat was greater in the period of mangrove dominance than in the salt marsh dominance time period (Table 3). This trend held in both Florida and on the larger geographic scale (i.e., the Americas; Table 3). Based on the above results, we consider the model to be validated by hindcast ground‐truthing.

**TABLE 2:** For the four mangrove species (top), the measure of habitat suitability is shown for each of the three regions examined for all six time periods.

Region	Time period	*Avicennia germinans*	*Conocarpus erectus*	*Laguncularia racemosa*	*Rhizophora mangle*	Average mangrove
NE Florida	1984–1989	127.4	24.9	68.5	40.5	65.3
	2001–2006	140.7	28.8	75.6	42.3	71.8
	2013–2018	196.9	41.7	150.9	87.9	119.4
	2021–2040	177.7	33.6	103.4	70.0	96.2
	2041–2060	173.9	34.5	92.1	67.5	92.0
	2061–2080	175.0	28.4	75.5	63.8	85.7
Florida	1984–1989	1141.8	526.7	1051.7	740.9	865.3
	2001–2006	1299.4	501.4	1090.5	825.9	929.3
	2013–2018	1502.3	731.9	1445.3	1332.0	1252.9
	2021–2040	1381.8	687.7	1227.0	1086.3	1095.7
	2041–2060	1363.6	692.5	1134.9	1018.0	1052.2
	2061–2080	1428.9	633.2	1145.8	1032.4	1060.1
Americas	1984–1989	12,928.4	11,707.3	10,947.2	11,172.9	11,688.9
	2001–2006	14,242.3	11,144.4	11,518.6	11,021.2	11,981.6
	2013–2018	13,878.3	11,047.5	10,674.8	10,949.7	11,637.6
	2021–2040	13,566.9	10,914.5	10,227.8	9740.8	11,112.5
	2041–2060	13,882.7	10,401.4	9304.6	8713.3	10,575.5
	2061–2080	14,395.7	9821.3	7948.7	7987.3	10,038.2

Region	Time period	*Batis maritima*	*Sesuvium portulacastrum*	*Spartina alterniflora*	*Sporobolus virginicus*	Average salt marsh
NE Florida	1984–1989	258.4	198.4	280.5	185.0	230.6
	2001–2006	271.7	148.3	288.9	163.1	218.0
	2013–2018	265.4	217.1	306.5	237.4	256.6
	2021–2040	261.5	243.2	273.9	273.8	263.1
	2041–2060	250.8	247.2	276.4	273.8	262.0
	2061–2080	254.2	261.5	278.9	256.5	262.8
Florida	1984–1989	1597.5	1163.5	1137.0	1271.9	1292.5
	2001–2006	1708.6	686.2	1371.2	1010.2	1194.0
	2013–2018	1635.6	929.3	1339.7	1391.3	1324.0
	2021–2040	1583.5	1161.6	1169.3	1456.6	1342.7
	2041–2060	1549.0	1230.1	1123.7	1461.9	1341.2
	2061–2080	1578.5	1305.5	1216.6	1330.6	1357.8
Americas	1984–1989	1,4517.5	12,383.0	8084.7	13,812.8	12,199.5
	2001–2006	15,197.3	12,776.0	8406.6	13,329.2	12,427.3
	2013–2018	14,524.1	12,578.8	8272.7	13,230.8	12,151.6
	2021–2040	13,875.3	10,805.1	6192.8	12,122.5	10,748.9
	2041–2060	14,878.5	9902.6	5814.7	11,527.0	10,530.7
	2061–2080	15,496.8	10,102.2	6094.5	10,607.0	10,575.1

*Note*: For each species, habitat suitability was calculated by the sum of all Cloglog values in each study region. The NE Florida and Florida regions were used for model hindcast validation and their geographic extent is defined in the text. The average habitat suitability across all mangrove species is shown in the rightmost column. The analogous values for the four salt marsh species are displayed in the bottom half of the table.

**TABLE 3:** The ratio of modeled mangrove:salt marsh suitable habitat for each time period and each of the three study regions.

Time period	NE Florida	Florida peninsula	Americas
1984–1989	0.283	0.669	0.958
2001–2006	0.330	0.778	0.964
Present	0.465	0.946	0.958
2021–2040	0.366	0.816	1.034
2041–2060	0.351	0.785	1.004
2061–2080	0.326	0.781	0.949

### 3.3 Projecting future distributions

**FIGURE 4:**
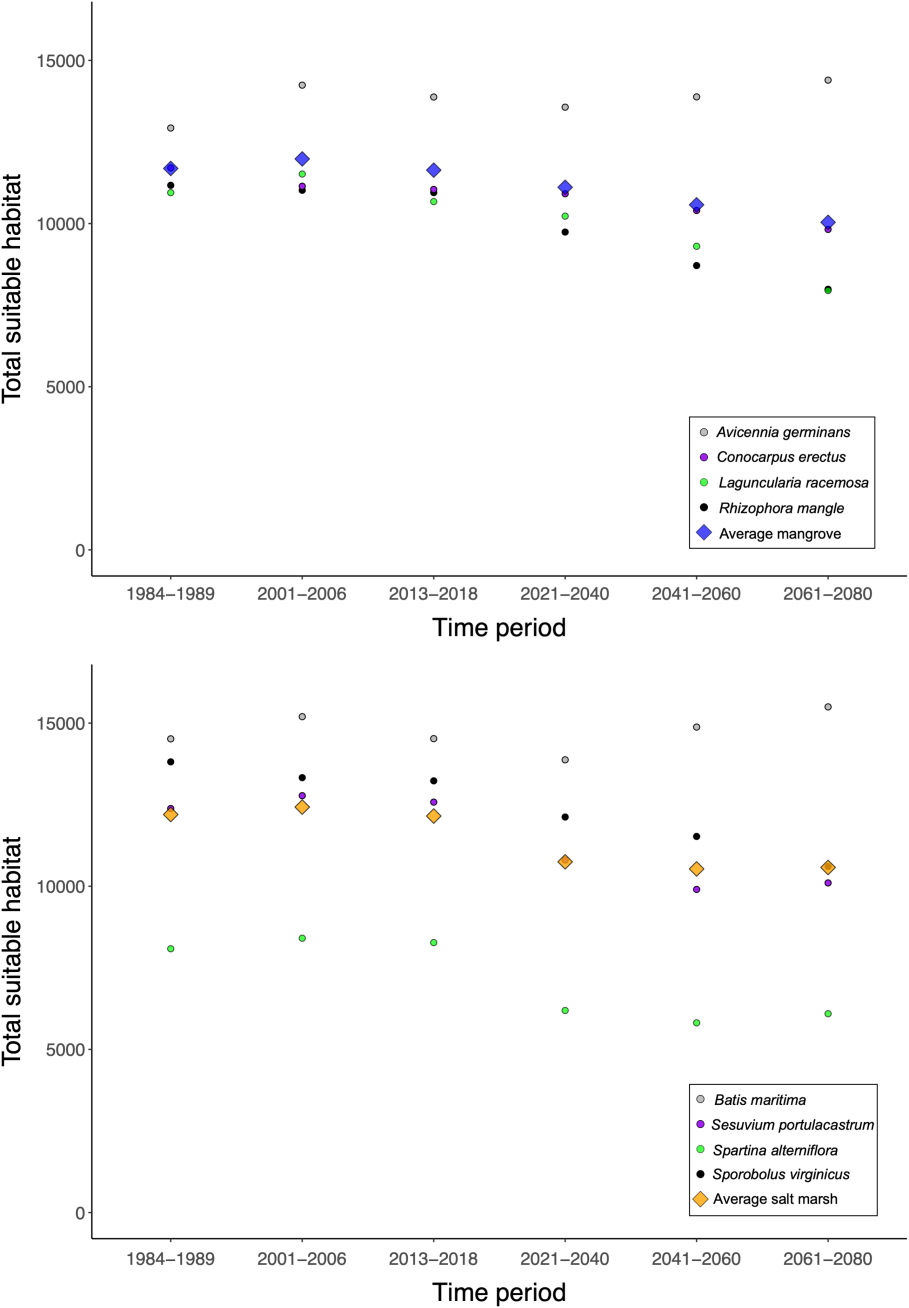
The SDM‐defined suitable habitat for the time periods in the past, present, and future on the large geographic scale (Americas). The total suitable habitat available for each mangrove species (top) and each salt marsh species (bottom) is shown using colored circles. The average across all four species is shown in colored diamonds.

In the NE Florida region, the hindcast‐validated SDMs displayed contrasting results between mangrove and salt marsh species when projected from the present to all future time periods. In the future time periods, there was a projected small decrease in average mangrove suitable habitat relative to the present and a projected small increase in salt marsh suitable habitat (Figure 3, Table 2). These trends also broadly held in the Florida validation region (Table 2; Figure S6). However, on the large geographic scale (i.e., the Americas), there was a decrease in suitable habitat relative to the present in both average mangrove and average salt marsh suitable habitat (Tables 2 and 4, Figure 4; Figures S7 and S8). These trends applied to all species except the mangrove species *Avicennia germinans* and the salt marsh species *Batis maritima*, which each underwent small predicted increases in suitable habitat in the time periods 2041–2060 and 2061–2080 relative to the present (Figure 4, Table 5). In future time periods, the ratio of mangrove:salt marsh suitable habitat was more similar to the past period of mangrove dominance versus the salt marsh dominance time period (Table 3). In the NE Florida and Florida validation regions, the ratio of mangrove:salt marsh suitable habitat was greater in the present than it was in any other time period and was typically greater in the future versus the past (Table 3). On the largest geographic scale, the ratio was greatest in the near future time periods (2021–2040, 2041–2060; Table 3).

**TABLE 4:** The percent change in suitable habitat between the present and the time period listed.

Time period	Species type	Region		
		NE Florida	Florida	Americas
1984–1989	Mangrove	−45.3	−30.9	0.4
	Salt marsh	−10.1	−2.4	0.4
2001–2006	Mangrove	−39.8	−25.8	3.0
	Salt marsh	−15.0	−9.8	2.3
2021–2040	Mangrove	−19.4	−12.5	−4.5
	Salt marsh	2.5	1.4	−11.5
2041–2060	Mangrove	−22.9	−16.0	−9.1
	Salt marsh	2.1	1.3	−13.3
2061–2080	Mangrove	−28.2	−15.4	−13.7
	Salt marsh	2.4	2.6	−13.0

*Note*: Within each time period, the percent change in habitat suitability averaged across the four mangrove species (top) and four salt marsh species (bottom) is shown. In the past, the 1984–1989 time period was dominated by salt marsh species, and the 2001–2006 time period was defined by mangrove dominance.

**TABLE 5:** Within each time period, the percent change in suitable habitat is listed for each of the four mangrove species (top) and each of the four salt marsh species (bottom) for each st region.

Time period	Species	Region		
		NE Florida	Florida	Americas
1984–1989	*Avicennia germinans*	−35.3	−34.0	−6.9
	*Conocarpus erectus*	−40.3	−28.0	6.0
	*Laguncularia racemosa*	−54.6	−27.2	2.6
	*Rhizophora mangle*	−53.9	−44.4	2.0
	*Batis maritima*	−2.6	−2.3	−0.1
	*Sesuvium portulacastrum*	−8.6	25.2	−1.6
	*Spartina alterniflora*	−8.5	−15.1	−2.3
	*Sporobolus virginicus*	−22.1	−8.6	4.4
2001–2006	*Avicennia germinans*	−28.5	−13.5	2.6
	*Conocarpus erectus*	−30.9	−31.5	0.9
	*Laguncularia racemosa*	−49.9	−24.5	7.9
	*Rhizophora mangle*	−51.9	−38.0	0.7
	*Batis maritima*	2.4	4.5	4.6
	*Sesuvium portulacastrum*	−31.7	−26.2	1.6
	*Spartina alterniflora*	−5.7	2.4	1.6
	*Sporobolus virginicus*	−31.3	−27.4	0.7
2021–2040	*Avicennia germinans*	−9.8	−8.0	−2.2
	*Conocarpus erectus*	−19.4	−6.0	−1.2
	*Laguncularia racemosa*	−31.4	−15.1	−4.2
	*Rhizophora mangle*	−20.4	−18.4	−11.0
	*Batis maritima*	−1.5	−3.2	−4.5
	*Sesuvium portulacastrum*	12.0	25.0	−14.1
	*Spartina alterniflora*	−10.6	−12.7	−25.1
	*Sporobolus virginicus*	15.0	4.7	−8.4
2041–2060	*Avicennia germinans*	−11.7	−9.2	0.1
	*Conocarpus erectus*	−17.3	−5.4	−5.8
	*Laguncularia racemosa*	−39.0	−21.5	−12.8
	*Rhizophora mangle*	−23.2	−23.6	−20.4
	*Batis maritima*	−5.5	−5.3	2.4
	*Sesuvium portulacastrum*	13.9	32.4	−21.2
	*Spartina alterniflora*	−9.8	−16.1	−29.7
	*Sporobolus virginicus*	15.3	5.1	−12.9
2061–2080	*Avicennia germinans*	−11.1	−4.9	3.7
	*Conocarpus erectus*	−31.9	−13.5	−11.1
	*Laguncularia racemosa*	−50.0	−20.7	−25.5
	*Rhizophora mangle*	−27.4	−22.5	−27.1
	*Batis maritima*	−4.2	−3.5	6.7
	*Sesuvium portulacastrum*	20.5	40.5	−19.7
	*Spartina alterniflora*	−9.0	−9.2	−26.3
	*Sporobolus virginicus*	8.0	−4.4	−19.8

*Note*: In the past, the 1984–1989 time period was dominated by salt marsh species, and the 2001–2006 time window was a period of mangrove dominance.

## 4 DISCUSSION

SDM approaches that use species occurrence data and environmental variables are valuable tools that can project future changes in suitable habitat, but one unavoidable limitation of these models is the impossibility of ground‐truthing the accuracy of the models in the future. Here, we use a novel validation approach: external data on the relative dominance of mangrove and salt marsh species in the previous century in an ecotone in NE Florida were used to validate our SDMs before projecting them into the future. We demonstrate a new way for researchers to ground‐truth SDMs to increase confidence when projecting SDMs into novel geographic or temporal space. We apply the hindcast‐validation method to project the potential future impacts of climate change on coastal angiosperm species, but our approach can be applied to a variety of research objectives using SDMs and to many different taxa across the Tree of Life. Using hindcast‐validated SDMs, our projections suggest a decline in suitable habitat in the future for nearly all mangrove and salt marsh species investigated (Figure 4). In most future time periods, the ratio of mangrove:salt marsh suitable habitat is projected to increase relative to the present across the Americas (Table 3). Below, we contextualize and discuss the results and offer recommendations for future research using hindcast‐validated SDMs.

When validating hindcast models, although the trends averaged across mangrove species and salt marsh species were consistent with expectations based on documented dominance, individual species did not always follow the expected trend. For example, in NE Florida, the salt marsh species *Batis maritima* and *Spartina alterniflora* exhibited slightly higher suitable habitat scores during the period of mangrove dominance (2001–2006) versus the period of salt marsh dominance (1989–1984). Although there are species‐specific differences when hindcasting, nevertheless the averaged results by species type (mangrove vs. salt marsh) all indicated proper model validation (Figures 2 and 3, Tables 2, 3, 4, 5; Figures S3–S6). We followed other large‐scale mangrove studies that grouped together and averaged species results to identify overarching trends (e.g., Osland et al., 2013; Record et al., 2013), and we also present results for each species separately (Table 5).

Overall, our results confirm findings of mangrove‐salt marsh oscillations in the NE Florida ecotone on the Atlantic coast of Florida reported in Cavanaugh et al. (2019). The present study also investigated a much larger geographic scope and therefore reveals key insights about the future dynamics between mangrove and salt marsh species at their range limit in the Southern Hemisphere and in the more central portions of these species' ranges. On a large geographic scale, in most future time periods, the ratio of mangrove:salt marsh suitable habitat is projected to increase relative to the present (Table 3). However, for most mangrove and salt marsh species studied, suitable habitat declines in the future time periods relative to the present. Therefore, future environmental conditions are projected to be detrimental to both species types, but they will be more favorable to mangroves, which may mean an increase in mangrove dominance at the expense of salt marsh species. It is possible that SLR could create new inland habitats suitable for mangrove and/or salt marsh species, although it is very difficult for many plant species to colonize new areas quickly enough to keep pace with changing climates (Corlett & Westcott, 2013).

### 4.1 Novelty and limitations of hindcast‐validated SDMs

We consider the hindcast validation approach to be an innovative way to use the fine temporal scale of WorldClim 2.1 data to ground‐truth SDMs and a template for how future SDM investigations can validate models before projecting them into the future or distant past (i.e., mid‐Holocene or earlier). When species have ample georeferenced data from different time periods between 1960 and 2018 and/or there are external data sources that can confirm species presence at specific times in the past, hindcast‐validated SDMs are a powerful tool for verifying models that will be used to project future suitable habitat. Researchers can follow our approach of constructing models for the present and hindcasting them, or if there are sufficient temporally diverse occurrence data, SDMs could be constructed for time periods in the past (e.g., 1960–1980) and validated using time periods closer to the present (e.g., 2000–2018) before being used to project future suitable habitat. Our methods will be easier to apply in certain species; the large availability of coastal photographs from many time periods and the relatively low species richness of mangrove and salt marsh communities compared with other communities facilitated our analyses. However, future studies on different species could leverage other data sources, such as flora and fauna checklists, to confirm species presence at certain time periods.

While a strength of our approach is the magnitude of the geographic range investigated, this also adds associated limitations. We validated the models using data from previous decades in the NE Florida ecotone, but projected future suitable habitat on a geographic extent larger than the validation area. Ideally, there would be external data sources that could be used for hindcast validation from other geographic regions, but to our knowledge there are no such data sources on the same timescale as the northeastern Florida ecotone data. The future projection results from our study align well with predictions in a small region of the Northern Hemisphere (Cavanaugh et al., 2019); therefore, it is reasonable to project our SDMs beyond that limited geographic region (i.e., Florida) because they could lead to critical insights about the future of these species in other regions (e.g., a mangrove‐salt marsh ecotone in southern Brazil).

A caveat associated with our study is that there were insufficient occurrence points for each species in each time period, given the short duration of the time periods (i.e., 6 years). Therefore, we followed a standard assumption in SDM analyses that the present is any time after 1950 (Hijmans et al., 2005). We used all occurrence data to construct an SDM for our defined present time (2013–2018) regardless of when the specimens were collected. While this is not an ideal approach and it would be preferable to have tens or hundreds of specimen records for each time period, that was impossible with the datasets used. We view the use of hindcast validation of SDMs as a valuable step forward for studies of this kind, despite this caveat.

One known limitation of SDMs is the difficulty in incorporating biotic interactions (e.g., competition or mutualisms) into a model. Especially at large scales, biotic interactions can have a significant effect on the predictive and explanatory power of models based on bioclimatic data (Araújo & Luoto, 2007). Some studies have successfully addressed biotic interactions on a spatial scale orders of magnitude smaller than the present study area (e.g., Crase et al., 2015). Bardou et al. (2021) incorporated species‐specific physiological data to refine distribution models of mangrove species on the Atlantic and Pacific coasts of North America. However, it remains prohibitively difficult to incorporate biotic interactions into an SDM framework on a large (i.e., continental) spatial scale. For this study, we prioritized using a large geographic extent to infer future suitable habitat in the ecotones in the Southern Hemisphere as opposed to investigating species interactions on a very limited geographic scale. We could project the future environmental niche of a species, but we could not fully understand how species interactions, such as competition, may affect a species' environmental niche in the future. Although our SDM approach does not explicitly consider species interactions, SDMs can be used to interpret interactions between species or groups of species. We measured whether a species or species type (mangrove vs. salt marsh) was projected to increase or decrease in suitable habitat. Any increase or decrease in suitable habitat may impact other species that occupy similar geographic ranges. Even though both species types are projected to decrease in suitable habitat in the future, the fact that the ratio of mangrove:salt marsh suitable habitat skews in favor of mangroves suggests interactions between the species with a likely result of mangroves occupying coastal habitats at the expense of salt marsh species.

Some projections of suitable habitat occurred outside of the documented ranges of many of these coastal species. For example, several mangrove species were projected to have suitable habitat adjacent to the Amazon River, hundreds of kilometers inland from their actual historically observed ranges (Figure 1). The model used abiotic factors to determine habitat suitability, and therefore the SDM analyses identified the pixels that contain suitable habitat for each species. These inland suitability scores >0 may indicate a misfit model, but we consider this unlikely; it is unsurprising that some of these regions would be categorized as moderately suitable habitat given that our SDMs are based on abiotic bioclimatic variables. Virtually all inland regions that were classified as suitable habitat were adjacent to rivers, and likely competition from rapidly growing riparian flora or physical dispersal limitations would prevent coastal species from colonizing these regions (Tomlinson, 2016). In fact, there are several documented instances of mangrove species occurring substantially inland (e.g., >50 km inland) in the Sundarbans in Bangladesh (Cornforth et al., 2013) and in the Yucatán peninsula (Aburto‐Oropeza et al., 2021), and several salt marsh species often occur tens of kilometers inland (Costa & Davy, 2013). Moreover, there are biotic explanations for the regions of unexpected inland suitable habitat. Competition from fast‐growing freshwater vascular plants has often been cited as the reason that mangroves are only found in coastal habitats (Simberloff, 1983; Tomlinson, 2016; Wang et al., 2011). Most inland regions where mangroves or salt marsh species could occur due to environmental tolerances, but are not present due to competition, have relatively low suitability scores (Figures 1 and 2; Figures S3–S5, S7, S8). We take this as evidence that the models are performing well; inland areas with implausible habitat suitability are regions where environmental conditions permit species existence, but biotic interactions (i.e., competition from fast‐growing riparian species) explain the absence of coastal taxa in these inland regions. However, rapid SLR in the future, which was not explicitly modeled, could mean that inland areas may soon experience increased salinity in the water, rendering the environment unsuitable for freshwater riparian species and enabling colonization by mangrove and/or salt marsh species.

### 4.2 Prospects and conclusions

We should carefully monitor areas where novel overlaps of salt marsh and mangrove species are projected, as species in these areas could be vulnerable to decline. Some processes that may be exacerbated by climate change, such as flooding, can have a different impact on mangrove versus salt marsh communities (Cruz et al., 2020; Schaeffer‐Novelli et al., 2016). The relative importance of climatic variables in our SDMs indicates that different factors may be more important for determining suitable habitat for each community—cold temperature events for mangroves and annual precipitation for salt marsh species (Table 1). As the climate changes, a decrease in cold events means mangrove species will have the potential to occupy areas that are currently inhabited by salt marsh or estuarine plant species. Meanwhile, changes in flooding and/or precipitation may enable salt marsh species to dominate in habitats that become unsuitable for mangroves (Cruz et al., 2020).

There is a fixed (or diminishing) amount of coastal land; any range increases will come at the expense of other coastal plant species. Although SLR could drastically affect coastal and estuarine regions by creating new suitable habitat for mangrove and/or salt marsh species, it will be challenging for plant species to occupy newly available habitat as the climate changes rapidly (Corlett & Westcott, 2013). In the majority of cases (e.g., Atlantic coast of North America), regions that are historically salt marshes will become suitable habitat for mangrove species based on our projections. Several ecological studies on small scales (e.g., Florida, Louisiana, Texas) have already documented mangrove invasions of salt marshes (Osland et al., 2013, 2020), which had negative impacts on native flora and fauna. Based on our SDM results, areas other than the Gulf of Mexico that may be at risk include southern Brazil. In southern Brazil, the salt marsh‐mangrove ecotone is in flux due to both human over‐development and the climate‐induced poleward shifts in mangroves (Schaeffer‐Novelli et al., 2016). The overlap of mangrove species and salt marsh species in the future will probably be detrimental to salt marsh species and may have a negative impact on mangrove species—biotic interactions with native species may mean that abiotic projections of suitable habitat overestimate their actual future range. Given the large spatial extent of our study, we view our results as a broad characterization of likely future trends in mangrove and salt marsh distributions, which should be further investigated by localized studies.

The projected future distribution change for the mangrove species modeled in this paper will likely not only negatively impact salt marsh species, but may also have unanticipated effects on the many taxa that depend on mangroves for survival. Many other organisms, including birds, fish, invertebrates, algae, and other plants (Cannicci et al., 2008; Lefebvre & Poulin, 1997; Nagelkerken et al., 2000; Rodriguez & Stoner, 1990; Tomlinson, 2016), rely on mangroves. Some species experience range shifts to keep pace with a changing climate; initial research suggests that mangroves are able to spread just quickly enough to adjust to climate change (Cavanaugh et al., 2014; Saintilan et al., 2014). However, it is unclear if the animal species depend on mangroves for food, shelter, and/or reproduction will be able to shift their ranges similarly as the climate changes. Some taxa are extirpated when species they depend on experience even minor range shifts (Foster, 2001). Additionally, as the various species that inhabit communities will likely experience range shifts at different rates, there is great potential for novel community assemblages in the future, as salt marsh flora and fauna interact with mangrove taxa (Lurgi et al., 2012). Our models can project the future suitable habitat of mangrove and salt marsh plants, but it is very difficult to predict how novel biotic interactions may impact the biodiversity associated with these two communities.

## AUTHOR CONTRIBUTIONS

**Richard G. J. Hodel:** Conceptualization (lead); data curation (lead); funding acquisition (supporting); methodology (lead); visualization (lead); writing – original draft (lead); writing – review and editing (lead). **Douglas E. Soltis:** Funding acquisition (supporting); methodology (supporting); supervision (equal); validation (equal); writing – review and editing (supporting). **Pamela S. Soltis:** Funding acquisition (lead); methodology (supporting); supervision (equal); validation (equal); writing – review and editing (supporting).

## CONFLICT OF INTEREST

The authors declare no conflict of interest.

## Supporting information


Appendix S1

## Data Availability

Bioclimatic data, sampling locations, MAXENT input files, and scripts are deposited in Dryad (https://doi.org/10.5061/dryad.08kprr55b) and GitHub (github.com/richiehodel/coastal_ENM).
