# Supplementary material for: Hindcast‐validated species distribution models reveal future vulnerabilities of mangroves and salt marsh species
Source: Ecol Evol. 2022 Sep 19;12(9):e9252. doi: 10.1002/ece3.9252 (PMC9484403; doi:10.1002/ece3.9252)
Supplement: Supplementary file 1 — Appendix S1 [file ECE3-12-e9252-s001.docx]

**Hindcast-validated species distribution models reveal future vulnerabilities of mangroves and salt marsh species**

**Supplementary Material**

Supplemental Figure S1 2

Supplemental Figure S2 3

Supplemental Figure S3 4

Supplemental Figure S4 7

Supplemental Figure S5 16

Supplemental Figure S6 25

Supplemental Figure S7 27

Supplemental Figure S8 33

Supplemental Table S1 58

Supplemental Table S2 59

Supplemental Table S3 75

**Supplemental Figure S1.** The geographic distribution of the GPS coordinates of occurrence data for each species.

**Supplemental Figure S2.** For each of the eight species, the partial Receiver Operating Characteristic (pROC) ratio, which is a measure of model performance calculated by dividing the partial AUC by random expectation, is shown.

**Supplemental Figure S3.** For the larger Florida validation region, the average suitable habitat for (**A**) mangrove species and (**B**) salt marsh species in two past time periods, the present, and three future time periods.

**Supplemental Figure S4.** For the smaller validation region (NE Florida), the projected suitable habitat in two past hindcast validation time periods, as well as the present, and three future time periods are shown for all eight study species. The summed habitat suitability scores for each time period on a per species basis are shown in Table 2.

**Supplemental Figure S5.** For the larger validation region (Florida), the projected suitable habitat in two past hindcast validation time periods, as well as the present, and three future time periods are shown for all eight study species. The summed habitat suitability scores for each time period on a per species basis are shown in Table 2.

**Supplemental Figure S6.** For the larger Florida validation region, the total suitable habitat available for each mangrove species (top) and each salt marsh species (bottom) are shown using colored circles. The average across all four species is shown in colored diamonds.

**Supplemental Figure S7.** The average suitable habitat for mangrove species (top) and salt marsh species (bottom) for the two past hindcast validation periods (**A**, **B**), and for three future time periods (**C-E**).

**Supplemental Figure S8.** For each study species, the projected suitable habitat in two past time periods, the present, and three future time periods for the entire geographic study region. The mangrove species are shown in **A-C** (*Avicennia germinans*), **D-F** (*Conocarpus erectus*), **G-I** (*Laguncularia racemosa*), and **J-L** (*Rhizophora mangle*). The salt marsh species are depicted in **M-O** (*Batis maritima*), **P-R** (*Sesuvium portulacastrum*), **S-U** (*Spartina alterniflora*), and **V-X** (*Sporobolus virginicus*).

**Supplemental Table S1.** The eight species, with the four mangrove/mangrove-associated species on top, and the four salt marsh species on the bottom, and the number of occurrences (after data cleaning) used in niche modeling.

| **Species** | **Number of occurrence points used** |
| --- | --- |
| *Avicennia germinans* | 367 |
| *Conocarpus erectus* | 404 |
| *Laguncularia racemosa* | 449 |
| *Rhizophora mangle* | 395 |
| *Batis maritima* | 166 |
| *Sesuvium portulacastrum* | 289 |
| *Spartina alterniflora* | 69 |
| *Sporobolus virginicus* | 192 |

**Supplemental Table S2.** The GPS coordinates of occurrence points used for SDM analyses. The four mangrove species are shown on the left and the data for salt marsh species are on the right.

| Mangrove species | | | | | | | | Salt marsh species | | | | | | | |
| --- | --- | --- | --- | --- | --- | --- | --- | --- | --- | --- | --- | --- | --- | --- | --- |
| *Avicennia germinans* | | *Conocarpus erectus* | | *Laguncularia racemosa* | | *Rhizophora mangle* | | *Batis maritima* |  | *Sesuvium portulacastrum* | | *Spartina alterniflora* | | *Sporobolus virginicus* | |
| Longitude | Latitude | Longitude | Latitude | Longitude | Latitude | Longitude | Latitude | Longitude | Latitude | Longitude | Latitude | Longitude | Latitude | Longitude | Latitude |
| -89.9527950 | 29.2619100 | -35.2166670 | -5.8000000 | -37.1430830 | -10.8413610 | -35.2750000 | -5.8416670 | -80.3818652 | 27.7086562 | -90.7173200 | 29.0517160 | -83.6025814 | 30.0477036 | -36.6344440 | -5.1150000 |
| -110.5790540 | 24.8343150 | -70.8056670 | 19.8661000 | -37.5833330 | -11.8833330 | -110.9838810 | 27.9634450 | -79.7467700 | 32.8140600 | -36.6344440 | -5.1150000 | -94.9558330 | 29.4652780 | -82.2637496 | 26.7529762 |
| -44.2238890 | -2.4827780 | -38.8666670 | -15.8500000 | -80.5059722 | 27.8320556 | -48.6985280 | -26.3376110 | -80.4352370 | 32.3850070 | -37.1080560 | -4.9475000 | -40.3375000 | -20.3197220 | -36.5000000 | -10.4880560 |
| -48.4901111 | -0.7252778 | -38.9500000 | -12.6166670 | -38.5000000 | -12.7608330 | -46.2563890 | -23.9930560 | -97.3666670 | 25.9500000 | -97.0530000 | 27.8276000 | -44.3425000 | -2.5308330 | -90.1874167 | 29.1030667 |
| -36.4394694 | -10.4922417 | -47.1132222 | -0.6817222 | -32.4250000 | -3.8361110 | -40.3375000 | -20.3197220 | -37.4242780 | -11.3780560 | -40.1555560 | -19.9538890 | -84.0190700 | 30.1279900 | -38.8666670 | -15.8500000 |
| -83.0360650 | 29.1424700 | -41.4173060 | -2.9280560 | -46.6125000 | -0.8194444 | -38.7064170 | -12.9987500 | -113.6170000 | 31.4167000 | -46.6190000 | -0.8355556 | -80.7630556 | 32.3983333 | -48.5093610 | -27.4346110 |
| -112.0507920 | 24.7557430 | -46.6250000 | -0.8375278 | -46.6394444 | -0.8402778 | -48.5232720 | -27.5797420 | -95.2400830 | 16.1685830 | -40.2291670 | -10.1002780 | -94.9598470 | 29.2032690 | -44.1817780 | -2.4636110 |
| -109.2333330 | 26.4041670 | -35.3736111 | -9.0227778 | -46.8854722 | -0.8390833 | -41.8362780 | -2.7941670 | -117.8847200 | 33.6291700 | -74.8370300 | -15.5623900 | -88.5136111 | 30.3475000 | -82.2954148 | 27.4710806 |
| -81.2861270 | 29.8917920 | -36.5216670 | -10.4000000 | -35.3736111 | -9.0227778 | -48.1344722 | -0.8234444 | -81.2287560 | 29.6869240 | -95.0549310 | 29.1357630 | -83.1576994 | 29.6109735 | -112.2000000 | 28.9700000 |
| -47.1186940 | -1.1533330 | -37.4258610 | -11.3855280 | -37.1450000 | -11.1288890 | -48.1172222 | -0.7666667 | -97.0679000 | 27.8378000 | -80.7981944 | 28.5630278 | -97.1014000 | 28.0258000 | -111.1083300 | 27.9666700 |
| -81.2287560 | 29.6869240 | -39.0333330 | -13.4833330 | -112.2019400 | 28.9686110 | -39.0731944 | -14.8109444 | -95.2102860 | 29.0130110 | -97.1226000 | 27.7445000 | -38.7500000 | -12.8666670 | -82.8219444 | 28.0236111 |
| -39.0575830 | -3.4128890 | -38.4000000 | -12.5833330 | -37.1369440 | -4.9561110 | -46.6374167 | -0.8402778 | -81.4203333 | 24.6533333 | -35.2166670 | -5.8000000 | -48.6844440 | -25.4522220 | -81.2363667 | 28.7172079 |
| -46.8829722 | -0.8466944 | -37.4483330 | -11.3508330 | -44.0447970 | -2.8658170 | -48.4838330 | -0.6640560 | -81.2509200 | 29.7508780 | -47.5613890 | -0.6063890 | -44.1025000 | -2.4169440 | -40.7833330 | -20.7833330 |
| -41.8290280 | -2.7864720 | -34.9083110 | -7.2606940 | -48.7085000 | -25.4284440 | -34.8438889 | -7.0583333 | -118.0597222 | 33.7088889 | -38.5000000 | -12.7608330 | -90.1841667 | 29.1386667 | -40.9688890 | -21.2802780 |
| -37.8497220 | -4.4036110 | -38.3500000 | -12.9333330 | -48.4875000 | -0.7119444 | -36.5552780 | -10.3719440 | -95.7652650 | 18.3644490 | -39.5613890 | -10.6736110 | -80.6632700 | 28.4946007 | -39.0491670 | -14.7891780 |
| -68.6678030 | 10.8820910 | -97.2170560 | 27.5778610 | -37.8497220 | -4.4036110 | -34.8019440 | -7.2777780 | -46.6205556 | -0.8408333 | -40.8058330 | -12.7900000 | -84.3684100 | 30.0277000 | -42.8263890 | -2.7469440 |
| -90.1875000 | 29.1055556 | -38.4525310 | -3.7912390 | -79.9190000 | 9.2970000 | -110.8997340 | 27.9181800 | -96.3800690 | 19.6032500 | -39.8166670 | -11.3666670 | -82.4598963 | 28.8525453 | -46.6737222 | -0.9145833 |
| -46.7123611 | -0.9372222 | -34.9166670 | -6.7666670 | -38.5775000 | -12.7016670 | -80.2683333 | 25.6750000 | -118.0847222 | 33.7433333 | -40.3077560 | -20.2779880 | -46.6053056 | -0.8556389 | -46.6094444 | -0.8222222 |
| -44.1648610 | -2.4564440 | -41.3347222 | -2.9250833 | -38.4527780 | -12.8005560 | -111.0617970 | 27.9507000 | -80.6632700 | 28.4946007 | -38.3166670 | -12.6833330 | -46.6171389 | -0.9634444 | -94.9738640 | 29.1898060 |
| -38.4750140 | -3.7574810 | -38.0833330 | -11.9333330 | -47.2956389 | -0.5991111 | -46.6560000 | -0.8908333 | -37.0997220 | -4.9322220 | -38.5000000 | -12.9666670 | -82.3061757 | 27.9312481 | -39.0666670 | -13.3666670 |
| -81.8313333 | 26.2946667 | -35.2916670 | -6.1833330 | -37.1166670 | -10.8669440 | -37.5433330 | -11.7786110 | -93.2264670 | 18.3706780 | -41.4173060 | -2.9280560 | -95.0816667 | 29.1200000 | -39.8238890 | -19.6455560 |
| -111.8916670 | 26.7583330 | -39.0166670 | -14.1833330 | -38.9419440 | -12.7091670 | -35.8352780 | -7.6744440 | -37.0386110 | -10.9088890 | -44.2319440 | -2.4819440 | -79.1232140 | 33.4277200 | -36.4427780 | -6.0844440 |
| -81.3411488 | 26.1174766 | -35.0941670 | -8.7136110 | -70.8056670 | 19.8661000 | -44.1025000 | -2.4169440 | -110.9838810 | 27.9634450 | -37.0716670 | -10.9111110 | -94.9527780 | 29.4200000 | -44.3168330 | -2.5058610 |
| -81.2540800 | 24.6682270 | -46.6094444 | -0.8222222 | -38.8833330 | -13.1000000 | -40.2442140 | -18.9481470 | -113.1500000 | 28.6333330 | -37.0202780 | -6.0338890 | -46.6125000 | -0.8194444 | -97.2638890 | 25.9963890 |
| -80.6632700 | 28.4946007 | -37.3102780 | -10.9983330 | -38.3166670 | -12.6833330 | -44.3425000 | -2.5308330 | -97.8201110 | 27.5146390 | -36.7841670 | -10.6772220 | -89.9563333 | 30.2622167 | -44.1025000 | -2.4169440 |
| -111.0617970 | 27.9507000 | -40.3375000 | -20.3197220 | -39.1166670 | -14.6833330 | -37.4380560 | -11.2686110 | -111.0670000 | 27.9500000 | -37.3441670 | -5.1875000 | -82.2954148 | 27.4710806 | -37.0386110 | -10.9088890 |
| -93.2264670 | 18.3706780 | -35.0919440 | -6.1869440 | -38.9833330 | -15.6666670 | -50.0107222 | 1.7116667 | -88.8491700 | 30.3755600 | -44.3146940 | -2.5010000 | -79.0463889 | 33.5316667 | -37.3416670 | -4.8833330 |
| -110.4333330 | 24.1500000 | -37.5269440 | -11.7555560 | -48.2969167 | -0.9345000 | -37.1369440 | -4.9561110 | -46.6666667 | -0.8833333 | -34.9786110 | -6.4936110 | -96.6880556 | 28.2127778 | -66.5116736 | 18.2187469 |
| -112.0996250 | 24.7891680 | -35.0641670 | -8.3994440 | -37.2063890 | -11.0147220 | -37.3594440 | -4.6955560 | -37.1369440 | -4.9561110 | -96.3800690 | 19.6032500 | -94.8716667 | 29.2841667 | -40.2166670 | -20.2336110 |
| -41.8463890 | -2.7848060 | -44.8734720 | -1.6373890 | -41.4178611 | -2.9109167 | -34.8566670 | -7.5319440 | -90.1841667 | 29.1386667 | -40.5069490 | -20.6488480 | -82.4186170 | 27.7623330 | -40.3266670 | -20.4069440 |
| -46.6666667 | -0.8833333 | -34.8338890 | -6.9811110 | -44.3189110 | -2.5408220 | -40.3041670 | -20.2766670 | -84.7996654 | 29.8901467 | -40.7561110 | -9.6147220 | -80.4682324 | 26.6461008 | -48.5594440 | -27.3152780 |
| -48.2969167 | -0.9345000 | -39.2658330 | -17.7319440 | -49.6095110 | -0.0568780 | -41.4178611 | -2.9109167 | -89.1030600 | 30.2263900 | -44.1516000 | -2.6466250 | -84.4039437 | 30.1705652 | -41.8055560 | -2.8833330 |
| -44.2727780 | -2.5963890 | -37.4380560 | -11.2686110 | -39.2694440 | -14.2788890 | -40.4197220 | -20.2641670 | -119.0850000 | 34.1100000 | -46.6275000 | -0.8155833 | -40.5108330 | -20.6580560 | -97.0891000 | 27.7979000 |
| -39.0500000 | -14.7905560 | -37.0541670 | -10.7891670 | -39.0325000 | -14.2788890 | -39.0333330 | -13.4833330 | -109.3310000 | 26.6500000 | -99.0500000 | 19.3333330 | -84.7996654 | 29.8901467 | -79.0429000 | 33.5250000 |
| -35.1258330 | -6.8388890 | -34.9786330 | -6.4936170 | -39.0333330 | -14.8000000 | -47.8055560 | -24.8950000 | -83.6025814 | 30.0477036 | -81.4205000 | 24.6581667 | -84.5125600 | 29.9168200 | -38.8833330 | -13.1000000 |
| -37.0541670 | -10.7891670 | -36.4166670 | -10.3833330 | -39.9666670 | -16.6166670 | -47.9294720 | -25.0364170 | -81.3411488 | 26.1174766 | -39.8166670 | -13.2833330 | -97.0458000 | 28.0257800 | -45.0055610 | -23.3963330 |
| -34.9372220 | -8.2375000 | -38.7500000 | -12.8666670 | -34.8427780 | -7.2341670 | -44.1659560 | -2.4574250 | -112.1975000 | 29.1833000 | -40.5613890 | -11.1894440 | -38.8666670 | -15.8500000 | -46.6501111 | -0.8739444 |
| -39.0744440 | -14.5250000 | -38.8333330 | -13.0333330 | -34.9886111 | -8.4523611 | -46.8851944 | -0.8391667 | -81.0439298 | 25.5225162 | -37.0120833 | -7.8937500 | -80.6577100 | 32.5247500 | -97.1688890 | 27.6772220 |
| -46.6148611 | -0.8164722 | -38.2833330 | -12.5166670 | -39.1500000 | -13.7666670 | -37.4483330 | -11.3508330 | -111.0386450 | 27.9610410 | -97.1695000 | 27.6834000 | -89.2830000 | 29.0830000 | -46.6685556 | -0.9237778 |
| -35.0114720 | -8.5254440 | -35.1824280 | -8.6363480 | -37.9425000 | -12.3777780 | -82.4088400 | 29.6121400 | -81.4073120 | 30.3789930 | -34.9772972 | -6.5850833 | -80.6099000 | 28.4171000 | -83.0408980 | 29.1489170 |
| -82.8219440 | 28.0236110 | -38.3166670 | -12.6833330 | -38.6166670 | -12.7333330 | -35.0508060 | -8.5948330 | -93.8083260 | 15.9337460 | -36.9886110 | -10.6483330 | -86.8138800 | 30.3888800 | -83.6025814 | 30.0477036 |
| -97.2197000 | 27.6281000 | -46.6170278 | -0.8363056 | -38.2833330 | -12.5166670 | -48.2969167 | -0.9345000 | -113.6138450 | 31.3897790 | -40.2818610 | -6.7148610 | -80.7500000 | 28.3000000 | -38.5000000 | -12.9666670 |
| -43.4558330 | -2.5988890 | -38.5000000 | -12.7608330 | -39.2500000 | -17.7333330 | -111.9833330 | 26.8833330 | -97.1963890 | 27.6480560 | -97.0891000 | 27.7979000 | -47.7000000 | -0.6166600 | -39.7322220 | -18.5933330 |
| -35.2583330 | -5.8833330 | -34.8427780 | -7.2341670 | -39.0772220 | -14.7905560 | -95.7652650 | 18.3644490 | -88.8700000 | 21.3900000 | -80.1474676 | 8.5084689 | -82.8219000 | 28.0236000 | -39.8588890 | -18.7161110 |
| -38.8666670 | -15.8500000 | -34.8594444 | -7.8191667 | -38.7072220 | -12.9950000 | -39.7346530 | -18.5925690 | -96.3800000 | 19.5800000 | -80.9616000 | 25.1350000 | -45.8950000 | -23.7525000 | -40.2733330 | -19.8205560 |
| -111.6833330 | 26.5833330 | -40.3041670 | -20.2766670 | -48.7933330 | -26.3066670 | -38.8666670 | -15.8500000 | -76.5324500 | 17.8603000 | -40.0533330 | -9.2519440 | -57.3572200 | -35.7072200 | -35.2166670 | -5.8000000 |
| -92.6489700 | 18.5322340 | -36.5000000 | -10.4880560 | -44.1659560 | -2.4574250 | -44.3027780 | -2.5297220 | -95.9588900 | 15.8300000 | -37.4483330 | -11.3508330 | -48.5097444 | -25.5241867 | -84.4039437 | 30.1705652 |
| -82.2954148 | 27.4710806 | -34.8438890 | -7.0583330 | -37.4169440 | -11.3922220 | -38.6211110 | -12.7413890 | -57.5666700 | 6.3333300 | -37.0779889 | -7.8817306 | -48.5749506 | -25.8869914 | -79.2385960 | 33.3515940 |
| -48.4926110 | -0.6603060 | -34.8025000 | -7.2772220 | -34.9786330 | -6.4936170 | -35.1824260 | -8.6363460 | -83.2926700 | 22.9530700 | -39.4169440 | -7.9727780 | -89.9781600 | 29.2555400 | -109.4166670 | 25.8000000 |
| -39.0333330 | -14.8000000 | -34.8630560 | -7.1150000 | -36.2313889 | -10.1161111 | -38.4458330 | -12.7741670 | -80.4330000 | -0.5500000 | -109.3830000 | 26.6389000 | -47.9200000 | -25.0200000 | -85.6255313 | 30.3051536 |
| -97.0886000 | 27.7982000 | -80.5168990 | 25.0097640 | -39.0736944 | -14.8052500 | -84.7996654 | 29.8901467 | -95.5333300 | 16.0333300 | -44.1819440 | -2.4655560 | -81.2416600 | 31.4775000 | -40.6000000 | -20.7833330 |
| -95.2012670 | 29.0212530 | -88.1666670 | 21.6000000 | -39.9833330 | -15.0966670 | -113.5666670 | 28.9333330 | -75.5798400 | 10.3346200 | -46.6125000 | -0.8194444 | -76.6861100 | 34.7216700 | -37.0761110 | -11.0094440 |
| -97.2541670 | 27.6930560 | -37.0716670 | -10.9111110 | -34.8019440 | -7.2777780 | -34.8427778 | -7.2341667 | -80.0000000 | -3.2660000 | -39.4548889 | -8.5454167 | -81.0847000 | 29.3486000 | -81.4534510 | 30.6340820 |
| -82.3906440 | 28.3093622 | -38.7000000 | -12.5333330 | -37.0386110 | -10.9088890 | -39.0333330 | -14.8000000 | -72.9166700 | 19.8500000 | -40.5565694 | -9.3310194 | -88.1205500 | 30.2561100 | -84.7996654 | 29.8901467 |
| -82.6148795 | 27.3629810 | -37.5833330 | -11.8833330 | -37.5313889 | -11.2716667 | -80.4030556 | -0.5888889 | -80.7333300 | -1.5166600 | -81.5468167 | 30.7210000 | -47.4000000 | -24.6500000 | -109.3530000 | 26.6583000 |
| -41.4178611 | -2.9109167 | -35.2247220 | -5.6913890 | -110.9838810 | 27.9634450 | -111.0386450 | 27.9610410 | -58.0333300 | 6.8000000 | -36.1350000 | -7.4816670 | -93.6636667 | 29.7501667 | -81.0439298 | 25.5225162 |
| -46.6194444 | -0.8355556 | -82.6558333 | 27.4155556 | -111.0617970 | 27.9507000 | -84.3645667 | 30.0457500 | -88.9719000 | 30.2113000 | -40.7494440 | -20.8061110 | -77.9208333 | 33.9788889 | -39.5166670 | -12.6000000 |
| -112.2345780 | 28.9830680 | -40.5108330 | -20.6580560 | -48.5563890 | -25.5175000 | -41.8215280 | -2.7786390 | -65.5883300 | 18.3872900 | -37.1427780 | -10.8594440 | -39.2500000 | -12.9666670 | -39.1000000 | -13.5833330 |
| -95.1106950 | 16.1877500 | -37.9425000 | -12.3777780 | -47.8055560 | -24.8950000 | -37.2063890 | -11.0147220 | -116.0000000 | 30.5000000 | -37.7341670 | -9.7307220 | -39.5508330 | -18.0866670 | -65.3316700 | 18.3111000 |
| -80.9417380 | 25.1387360 | -39.5666670 | -16.3666670 | -46.4429722 | -0.8845278 | -80.2809700 | 25.6581500 | -57.9666600 | 6.6666600 | -37.0555560 | -6.3944440 | -96.7067690 | 28.4056190 | -80.8000000 | -1.5666700 |
| -80.9085240 | 25.1472840 | -37.8900000 | -12.3291670 | -37.3102780 | -10.9983330 | -40.4666670 | -20.6002780 | -88.2530500 | 17.4613800 | -44.1648610 | -2.4564440 | -95.2055560 | 29.0175000 | -88.8884000 | 30.2365000 |
| -80.5710377 | 25.6136904 | -35.8352780 | -7.6733330 | -82.6558333 | 27.4155556 | -48.4997220 | -1.4077780 | -79.7261100 | 8.6316700 | -37.2780560 | -9.9172220 | -90.1866667 | 29.1035000 | -89.6200000 | 21.0400000 |
| -81.8560242 | 26.4313981 | -44.1516000 | -2.6466250 | -93.2264670 | 18.3706780 | -37.4200000 | -11.3938890 | -87.4333300 | 13.2100000 | -79.0467000 | 33.5188000 | -95.0767280 | 29.7512610 | -64.6558300 | 10.2094400 |
| -81.0439298 | 25.5225162 | -37.0386110 | -10.9088890 | -68.6678030 | 10.8820910 | -40.5108330 | -20.6580560 | -57.9000000 | 6.6333300 | -40.3277780 | -9.6147220 | -93.4755000 | 29.8048333 | -86.7500000 | 15.7833300 |
| -82.2637496 | 26.7529762 | -36.4972222 | -10.1841667 | -47.8375560 | -24.8902780 | -34.8813890 | -8.0544440 | -57.9200000 | 6.6333300 | -40.5500000 | -11.7166670 | -38.8833330 | -13.1000000 | -81.2416600 | 31.4775000 |
| -95.7652650 | 18.3644490 | -82.6136600 | 27.5846600 | -32.4162160 | -3.8455430 | -35.0641670 | -8.3994440 | -71.9853000 | 12.2492900 | -38.7500000 | -12.8666670 | -81.3612662 | 29.9970988 | -93.2200000 | 29.8700000 |
| -48.8742560 | -1.7295030 | -80.2683333 | 25.6750000 | -40.5108330 | -20.6580560 | -46.6666667 | -0.8833333 | -71.1800000 | 18.3300000 | -40.8341670 | -11.6916670 | -84.2048600 | 30.1345300 | -83.0700000 | 10.0000000 |
| -48.5172220 | -0.7277780 | -37.1450000 | -11.1288890 | -80.7981944 | 28.5630278 | -39.9833330 | -15.0966670 | -118.2069000 | 33.7583000 | -37.2610583 | -8.0309806 | -77.6305500 | 34.3652700 | -78.0291700 | 9.2291700 |
| -50.0084444 | 1.6937222 | -37.5113890 | -11.5194440 | -112.0000000 | 26.0000000 | -37.0386110 | -10.9088890 | -79.8800000 | -2.2300000 | -89.1030600 | 30.2263900 | -39.1000000 | -16.4500000 | -112.0833300 | 24.8166700 |
| -37.0386110 | -10.9088890 | -39.1166670 | -14.6833330 | -36.4972222 | -10.1841667 | -37.0541670 | -10.7891670 | -117.8800000 | 33.6200000 | -97.3000000 | 27.4500000 |  |  | -89.6667000 | 21.2833000 |
| -35.1420560 | -6.7418610 | -39.0666670 | -13.3666670 | -80.2504800 | 25.7153000 | -47.8752778 | -0.6972222 | -117.9991600 | 33.6602700 | -84.5106800 | 29.9176000 |  |  | -61.4184000 | 15.3951000 |
| -97.1579720 | 25.9987780 | -47.8947222 | -0.5569444 | -38.8333330 | -13.0333330 | -57.8244750 | -6.2066030 | -88.1600000 | 21.5800000 | -37.1369440 | -4.9561110 |  |  | -67.9300000 | 18.0800000 |
| -80.2683333 | 25.6750000 | -34.9972220 | -7.3811110 | -38.3166670 | -12.8833330 | -42.6833330 | -2.6000000 | -65.7666600 | 18.3833300 | -58.1166670 | -28.7166670 |  |  | -75.7000000 | 10.1333300 |
| -90.1841667 | 29.1386667 | -95.5333300 | 16.0333300 | -44.3425000 | -2.5308330 | -38.9194440 | -12.7783330 | -75.4833300 | 10.5500000 | -40.7669440 | -9.5394440 |  |  | -80.5000000 | -0.7833300 |
| -84.7996654 | 29.8901467 | -79.9733300 | 9.3583300 | -44.8069440 | -2.9555560 | -46.7833330 | -24.1666670 | -80.5477000 | 28.4991000 | -37.5113890 | -11.5194440 |  |  | -74.6284100 | 11.0166400 |
| -112.2393880 | 27.2345400 | -85.6555500 | 10.7916600 | -47.1192222 | -0.6843333 | -38.3368060 | -12.9238890 | -95.1333300 | 29.0669400 | -95.2099720 | 29.0118470 |  |  | -43.5350000 | -23.0500000 |
| -37.1369440 | -4.9561110 | -86.8333300 | 12.2583300 | -34.8338890 | -6.9811110 | -38.9833330 | -15.6666670 | -88.2005600 | 17.5144400 | -65.2833300 | 18.3166700 |  |  | -79.6916600 | 9.5000000 |
| -39.5508330 | -18.0866670 | -80.4166600 | -0.5000000 | -47.5745500 | -0.5887360 | -47.1192222 | -0.6843333 | -87.6000000 | 19.7666700 | -83.7833300 | 11.0166600 |  |  | -88.0291700 | 17.7369400 |
| -46.6930556 | -0.9180556 | -76.2500000 | 4.0333300 | -109.2333330 | 26.4166670 | -47.0866667 | -0.8538889 | -88.2408300 | 17.5325000 | -87.1500000 | 12.8166700 |  |  | -97.0000000 | 20.4800000 |
| -50.0093889 | 1.6457778 | -52.8000000 | 4.1511100 | -37.0541670 | -10.7891670 | -38.3166670 | -12.6833330 | -57.7800000 | 6.5833300 | -83.4875000 | 8.4597200 |  |  | -65.2879200 | 18.3158000 |
| -37.3102780 | -10.9983330 | -83.4666700 | 8.5166700 | -34.8566670 | -7.5319440 | -69.0800000 | 18.4200000 | -117.2292200 | 32.7522400 | -77.3833300 | 8.6666700 |  |  | -79.8333300 | 0.8500000 |
| -36.4658330 | -10.4294440 | -47.6800000 | -0.6166700 | -35.2583330 | -5.7666670 | -67.1468400 | 18.0563500 | -74.1400000 | -9.1839000 | -82.2450000 | 9.3433300 |  |  | -60.6670000 | 11.2500000 |
| -65.2895300 | 18.2937400 | -39.1200000 | -13.4666700 | -38.8666670 | -15.8500000 | -90.0000000 | 13.7000000 | -71.6500000 | 17.9200000 | -58.4166600 | -25.1166600 |  |  | -81.1047000 | 24.7011000 |
| -86.9000000 | 20.5500000 | -84.6700000 | 10.7900000 | -44.3418390 | -2.5772030 | -86.5166600 | 11.7833300 | -117.1083000 | 32.6491000 | -66.8486100 | 17.9538800 |  |  | -67.2000000 | 17.9700000 |
| -87.4333300 | 15.8000000 | -80.6416600 | 25.2966600 | -38.9166670 | -12.7666670 | -88.3233300 | 16.9500000 | -87.7000000 | 21.5152700 | -93.4610000 | 29.7700000 |  |  | -86.5783000 | 30.3961000 |
| -67.2000000 | 17.9583300 | -77.6333300 | 6.9666700 | -38.5000000 | -12.9666670 | -92.6100000 | 18.2600000 | -88.0300000 | 17.7364000 | -59.2166700 | -23.2166700 |  |  | -90.3600000 | 20.8500000 |
| -85.6833300 | 10.8111100 | -79.7500000 | 9.4450000 | -37.6333330 | -11.8000000 | -83.4000000 | 14.0416600 | -98.0300000 | 22.2200000 | -82.6408300 | 9.6333300 |  |  | -64.7584000 | 17.7790000 |
| -85.7375000 | 10.9222200 | -90.0136100 | 21.1661100 | -43.4558330 | -2.5988890 | -80.1583300 | 9.2000000 | -62.6564000 | 17.2494000 | -86.3388800 | 11.6416600 |  |  | -87.3938800 | 15.7741600 |
| -88.1166600 | 18.3500000 | -85.7319400 | 10.9361100 | -37.0716670 | -10.9111110 | -39.0000000 | -14.1000000 | -74.7036100 | 11.0082100 | -85.0928000 | 9.6015300 |  |  | -86.7300000 | 21.2300000 |
| -74.4931700 | 10.9819300 | -105.2500000 | 21.5333300 | -36.4658330 | -10.4294440 | -85.7833300 | 11.1333300 | -105.2500000 | 21.5333300 | -88.2005500 | 17.5144400 |  |  | -80.5330000 | -1.8500000 |
| -75.8249700 | 9.3999400 | -80.4500000 | -0.5200000 | -68.6300000 | 18.8500000 | -80.2160000 | -2.7830000 | -97.9500000 | 22.2200000 | -80.0500000 | 9.2833300 |  |  | -87.5000000 | 15.7727800 |
| -110.3061100 | 24.2316700 | -85.7300000 | 10.9000000 | -78.2000000 | 8.6666700 | -69.5500000 | 19.3166700 | -80.9327700 | 25.1741600 | -99.4819400 | 17.2875000 |  |  | -76.8166700 | 18.2833300 |
| -76.7429700 | 8.0903700 | -77.3500000 | 25.0833300 | -85.6166600 | 10.8333300 | -65.9633300 | 18.4522200 | -93.8677800 | 16.0616700 | -80.4000000 | -0.5830000 |  |  | -87.4877700 | 15.7825000 |
| -87.6286100 | 15.9125000 | -42.1891700 | -22.8502800 | -88.2416600 | 17.5347200 | -82.1166700 | 8.9500000 | -67.2000000 | 17.9666600 | -65.9794000 | 18.4581000 |  |  | -80.9166600 | -2.2500000 |
| -88.9500000 | 13.3500000 | -66.9156300 | 17.9785000 | -80.0166600 | -3.3000000 | -83.6530400 | 8.5283300 | -87.5719400 | 13.3783300 | -94.9124000 | 16.5723000 |  |  | -40.6456000 | -20.8058000 |
| -77.3500000 | 6.0333300 | -90.3600000 | 20.8500000 | -85.3722200 | 9.8444400 | -79.7305600 | 9.4666700 | -80.8800000 | 25.2000000 | -52.3333300 | 4.9333300 |  |  | -76.1374700 | 24.8596300 |
| -57.3333300 | 6.0000000 | -90.0000000 | 13.7000000 | -84.4225900 | 9.5197100 | -88.8697200 | 15.7719400 | -97.9300000 | 22.2000000 | -85.6619700 | 10.7752500 |  |  | -80.6660000 | -1.6160000 |
| -75.5105600 | 10.3569400 | -87.4500000 | 13.4166700 | -80.4160000 | -0.5000000 | -77.4666600 | 4.2500000 | -117.9455000 | 33.6454000 | -66.5188500 | 17.8946400 |  |  | -89.6500000 | 21.1500000 |
| -65.7666600 | 18.3833300 | -89.0655500 | 13.3672200 | -80.3503000 | 27.5281000 | -83.3666600 | 14.0833300 | -80.9328000 | 25.1512000 | -65.4616700 | 18.0972200 |  |  | -51.0788277 | -26.2421851 |
| -62.0666600 | 10.6500000 | -70.5800000 | 18.2900000 | -35.4344400 | -5.8950000 | -68.5200000 | 18.7700000 | -105.2833300 | 21.5500000 | -39.1000000 | -11.9000000 |  |  | -72.0333300 | 11.4500000 |
| -85.3000000 | 10.3400000 | -84.9600000 | 9.8000000 | -83.6880600 | 12.3394400 | -77.1475000 | 18.1869000 | -66.8486100 | 17.9538800 | -65.0500000 | -24.6666700 |  |  | -71.7389200 | 21.8230800 |
| -43.2076686 | -22.9066598 | -74.1500000 | 11.2666700 | -85.7489800 | 11.0284300 | -34.9541700 | -8.2800000 | -81.4400000 | 24.7183300 | -87.9725000 | 13.1600000 |  |  | -111.9000000 | 26.7600000 |
| -44.0408262 | -22.9635597 | -79.9000000 | -2.2000000 | -92.1400000 | 18.5500000 | -81.7452700 | 7.6366600 | -71.5000000 | 18.3700000 | -98.9200000 | 19.4600000 |  |  | -61.2486000 | 10.4606000 |
| -65.5884200 | 18.3866900 | -83.7555600 | 9.1555600 | -87.1500000 | 12.8166700 | -88.9436100 | 15.7161100 | -72.6300000 | 19.5700000 | -90.1870000 | 29.1030000 |  |  | -41.6383300 | -22.2733300 |
| -86.7000000 | 12.0500000 | -44.3030252 | -2.5330733 | -83.3333300 | 8.7333300 | -111.8925000 | 26.7513900 | -111.2588900 | 25.7419400 | -42.0347200 | -22.9438900 |  |  | -88.2188900 | 16.9555600 |
| -83.6503200 | 8.5262900 | -86.1750000 | 11.5250000 | -85.7311100 | 10.9019400 | -92.9552700 | 18.3527700 | -118.0500000 | 33.7026000 | -92.5000000 | 14.8000000 |  |  | -70.0000000 | 19.3666700 |
| -85.7489800 | 11.0284200 | -87.1666600 | 12.5166600 | -80.1505500 | 9.1925000 | -90.6647200 | 13.9261100 | -66.9046200 | 17.9616200 | -88.9689000 | 30.2131000 |  |  | -86.9400000 | 20.7900000 |
| -91.8300000 | 18.6400000 | -96.2383300 | 15.6852700 | -43.0407567 | -22.6569560 | -85.7375000 | 10.9222200 | -86.9808300 | 20.3066600 | -83.5000000 | 10.5333300 |  |  | -40.0200000 | -13.0200000 |
| -89.8200000 | 21.2400000 | -79.0500000 | 1.2000000 | -65.5884200 | 18.3866900 | -47.9298727 | -15.7834208 | -86.8333300 | 12.2583300 | -64.9369400 | 18.3552700 |  |  | -43.6277800 | -23.0491700 |
| -85.6166600 | 10.8333300 | -87.9166600 | 13.1833300 | -65.4235600 | 18.1294000 | -80.7500000 | -2.1166700 | -71.9031700 | 12.1968400 | -63.7666700 | 10.6500000 |  |  | -87.2569000 | 30.3199000 |
| -95.5333300 | 16.0333300 | -79.9497200 | 9.3705600 | -71.8333300 | 11.0333300 | -86.7333300 | 12.1500000 | -88.9500000 | 21.3800000 | -66.1358300 | -17.4172200 |  |  | -90.7000000 | 19.5500000 |
| -78.8351500 | -1.2862600 | -74.3977200 | 10.9751500 | -75.5772500 | 9.5230700 | -59.7552700 | 8.3983300 | -117.1197200 | 32.5452800 | -88.3938800 | 17.4613800 |  |  | -97.2333300 | 25.9500000 |
| -80.2160000 | -2.7330000 | -90.3666700 | 20.2500000 | -81.2500000 | 8.1667000 | -85.6700000 | 10.5700000 | -117.2541700 | 32.9680600 | -80.3500000 | 7.9666700 |  |  | -110.2170000 | 23.4100000 |
| -71.8333300 | 11.0333300 | -82.1633300 | 8.2200000 | -76.7452400 | 8.1853500 | -84.8300000 | 9.9700000 | -70.8300000 | 18.3500000 | -87.2102700 | 12.5272200 |  |  | -42.0313900 | -22.8986100 |
| -87.7922200 | 13.4355500 | -85.6555700 | 10.7916700 | -39.0142849 | -14.1069915 | -80.1500000 | -2.9500000 | -87.0000000 | 20.3100000 | -77.3500000 | 6.2666600 |  |  | -83.7833300 | 11.0166600 |
| -87.9000000 | 21.0000000 | -89.8186100 | 13.5916600 | -79.0500000 | 1.2000000 | -91.1500000 | 17.7700000 | -81.3727700 | 24.6922200 | -88.1069400 | 30.2577800 |  |  | -75.7081000 | 19.9603000 |
| -78.0166600 | 7.3750000 | -75.5816100 | 9.8691100 | -94.4400000 | 16.2300000 | -87.0166600 | 12.3666600 | -72.4988700 | 11.7412200 | -40.4200000 | -20.2639000 |  |  | -70.0411000 | -19.1982000 |
| -65.6309300 | 18.3720400 | -75.5022400 | 10.3015100 | -82.3433300 | 9.2000000 | -88.2500000 | 17.5333300 | -88.1166600 | 18.3500000 | -69.2200000 | 18.4700000 |  |  | -87.8786100 | 15.8816600 |
| -86.7300000 | 21.2300000 | -42.2333300 | -22.9166700 | -89.0000000 | 13.3500000 | -88.3269400 | 17.4727700 | -75.2500000 | 10.7500000 | -67.0757000 | 18.5152000 |  |  | -61.3333000 | 15.4167000 |
| -79.0500000 | 1.2000000 | -85.1388800 | 9.5980500 | -87.6000000 | 15.8333300 | -88.2005500 | 17.5144400 | -76.4333300 | 8.8500000 | -74.0640100 | 11.3137100 |  |  | -38.5429580 | -3.7206118 |
| -44.2858400 | -2.4884600 | -88.3333300 | 17.4666600 | -80.4666600 | -3.5000000 | -90.3700000 | 20.1600000 | -74.5366600 | 10.9858300 | -66.8333300 | 18.0000000 |  |  | -88.4439000 | 30.2010000 |
| -84.7016700 | 9.8827800 | -84.9027800 | 9.9500000 | -79.8333300 | 0.8666600 | -89.3527700 | 18.7725000 | -82.7367000 | 29.0417000 | -79.5719400 | 8.9305600 |  |  | -80.0000000 | -3.2660000 |
| -83.2125000 | 14.3083300 | -87.6286100 | 15.9125000 | -84.1166600 | 11.2666600 | -87.7955600 | 21.5436100 | -105.0244400 | 19.4358300 | -80.3166600 | 7.9000000 |  |  | -86.7772200 | 15.7972200 |
| -85.8666600 | 11.2500000 | -80.5333300 | 8.1583300 | -83.3333300 | 14.1000000 | -47.6800000 | -0.6166700 | -65.4452700 | 18.1494400 | -97.1866600 | 27.6450000 |  |  | -89.6600000 | 21.2800000 |
| -94.0805600 | 18.0980600 | -96.3811000 | 19.5900000 | -88.2100000 | 18.7200000 | -81.3541700 | 24.6694400 | -97.5333300 | 16.0000000 | -82.8430600 | 9.7416700 |  |  | -86.9224000 | 30.3746000 |
| -105.2833300 | 21.5500000 | -80.0300000 | 0.3700000 | -77.8333300 | 8.1500000 | -68.8500000 | 18.9666700 | -78.9886100 | 22.6530600 | -79.8155600 | 0.8905600 |  |  | -87.6200000 | 18.9500000 |
| -72.3500000 | 18.5400000 | -83.2926700 | 22.9530700 | -62.7833300 | 10.0333300 | -73.3666700 | 19.8333300 | -72.1498100 | 12.1959500 | -48.5171100 | -27.1219200 |  |  | -39.9833300 | -14.2666700 |
| -85.6500000 | 10.7930600 | -78.8351500 | -1.2862600 | -75.0000000 | 10.7500000 | -84.6700000 | 10.7900000 | -90.3300000 | 20.8500000 | -70.3166600 | -18.4666600 |  |  | -89.6500000 | 21.1800000 |
| -65.8333300 | 18.4166600 | -38.9300000 | -13.9300000 | -79.9566600 | 9.3641600 | -69.2800000 | 18.4700000 | -96.6667000 | 19.3333000 | -80.7333300 | -0.9500000 |  |  | -77.9500000 | 24.4333000 |
| -77.2833300 | 5.3166600 | -78.8000000 | -1.4166000 | -93.0800000 | 18.3200000 | -39.0441000 | -16.2986200 | -90.0220000 | 29.4988000 | -80.7666700 | -1.4833300 |  |  | -80.3069400 | 9.1552700 |
| -75.6086100 | 9.4717400 | -83.3000000 | 14.1333300 | -61.4333300 | 10.6166700 | -64.3400000 | 10.3500000 | -83.0267000 | 29.1703000 | -64.7606000 | 18.3172000 |  |  | -66.5196000 | 17.8944000 |
| -44.0448000 | -2.8658200 | -79.8800000 | 9.3713800 | -90.8236100 | 13.9200000 | -65.2908200 | 18.2929000 | -92.8108300 | 15.2108300 | -87.6200000 | 18.9500000 |  |  | -87.0000000 | 15.6667000 |
| -86.3388900 | 11.6416700 | -80.4833300 | 8.4333300 | -79.8833300 | -2.2333300 | -86.2161100 | 11.5827700 | -89.9400000 | 21.2000000 | -78.4783300 | -0.1586100 |  |  | -66.4416700 | 18.4652800 |
| -85.2200000 | 10.2100000 | -67.2000000 | 17.9583300 | -65.1322200 | 10.0661100 | -94.1155600 | 18.1677800 | -94.9610278 | 29.1978056 | -77.8825000 | 9.1166700 |  |  | -67.1906500 | 17.9393100 |
| -85.7305600 | 10.9027800 | -90.3861100 | 20.8583300 | -65.8166600 | 18.3666600 | -80.3166600 | 7.9000000 | -97.1730556 | 27.6794444 | -80.7333300 | -1.5166600 |  |  | -86.8225000 | 21.1483300 |
| -87.1666600 | 12.5166600 | -85.1610900 | 10.1077500 | -83.2926700 | 22.9530700 | -79.9566600 | 9.3641600 | -81.4088833 | 30.9515833 | -76.4333300 | 8.8500000 |  |  | -93.2000000 | 18.4100000 |
| -71.9666600 | 12.0833300 | -84.6900000 | 9.8600000 | -69.5900000 | 18.4500000 | -95.0000000 | 18.5166600 | -119.8400000 | 34.4220000 | -85.6500000 | 10.5800000 |  |  | -82.4303000 | 27.0461000 |
| -65.4235500 | 18.1295500 | -88.8000000 | 13.2500000 | -61.3000000 | 15.3700000 | -80.8492000 | 25.2255000 | -113.6075592 | 31.3548097 | -84.2800000 | 9.4900000 |  |  | -43.2076686 | -22.9069598 |
| -90.3700000 | 20.1600000 | -79.1269400 | 8.2583300 | -71.0750000 | 18.1700000 | -86.5833000 | 12.5833000 | -90.1875000 | 29.1055556 | -80.7160000 | -2.0500000 |  |  | -68.3333300 | 10.7333300 |
| -76.4333300 | 6.3500000 | -88.3333300 | 13.2000000 | -48.7811783 | -28.4867241 | -84.9055700 | 9.9465200 | -94.9583330 | 29.2000000 | -39.8589000 | -18.7161000 |  |  | -80.7500000 | -2.1000000 |
| -85.8694400 | 11.2513900 | -65.9416700 | 18.4416700 | -86.7500000 | 12.1666600 | -65.6525000 | 18.3258300 | -119.0725457 | 34.0938418 | -80.1916600 | 9.1500000 |  |  | -93.4160000 | 29.7680000 |
| -82.6200000 | 9.6000000 | -88.4725500 | 17.7674800 | -83.8000000 | 12.0500000 | -59.5789000 | 13.0734000 | -84.4039437 | 30.1705652 | -43.0407567 | -22.6569560 |  |  | -61.3833000 | 15.3000000 |
| -42.3000000 | -22.6000000 | -80.3547200 | 7.9761100 | -76.2175700 | 8.9406600 | -34.8597200 | -7.8186100 | -37.5313889 | -11.2716667 | -59.0000000 | -21.0000000 |  |  | -40.8338900 | -21.0111100 |
| -79.9000000 | -2.2000000 | -82.1666600 | 8.3166600 | -83.4333300 | 14.0166600 | -90.6900000 | 19.2700000 | -113.6296770 | 31.3406580 | -80.4500000 | -0.5160000 |  |  | -80.9200000 | -2.2500000 |
| -92.6880500 | 18.4922200 | -79.9330000 | -2.7330000 | -76.7666600 | 8.3000000 | -71.3997200 | 3.3522200 | -93.2000000 | 18.4100000 | -67.9377800 | 18.0891700 |  |  | -63.7666600 | 10.6500000 |
| -94.0305500 | 18.2083300 | -83.5500000 | 8.4500000 | -92.5188800 | 18.5122200 | -83.3333300 | 14.0916700 | -57.9700000 | 6.6666700 | -57.7833300 | 6.5833300 |  |  | -80.2166600 | -2.7333300 |
| -79.1116700 | 8.2580600 | -83.4166600 | 14.1666600 | -89.7200000 | 21.2700000 | -92.6333300 | 18.4833300 | -77.6505300 | 26.8976700 | -76.6333000 | -12.0000000 |  |  | -86.9458000 | 20.5083000 |
| -80.5333300 | 8.2666600 | -79.1047200 | 8.2494400 | -78.5254000 | 1.8056100 | -92.5205500 | 18.4788800 | -88.1955500 | 17.4841600 | -42.0278871 | -22.9699618 |  |  | -48.6622617 | -26.9120048 |
| -78.5552700 | 1.7400000 | -88.1500000 | 21.5700000 | -48.5733300 | -24.1566700 | -59.7000000 | 8.3722200 | -118.0304000 | 33.7113000 | -85.2208300 | 10.2930600 |  |  | -66.7315400 | 18.4213900 |
| -79.9500000 | -2.2160000 | -88.1166700 | 18.3500000 | -77.2833300 | 5.3166600 | -84.4600000 | 9.5300000 | -118.0388900 | 33.6916700 | -64.6558300 | 10.2094400 |  |  | -48.4776900 | -27.6525500 |
| -38.9965836 | -14.2814954 | -76.4333300 | 6.3500000 | -87.1666600 | 12.8500000 | -85.8644400 | 10.9194400 | -75.7000000 | 10.1333300 | -87.7300000 | 21.3900000 |  |  | -74.5000000 | 10.9666600 |
| -88.0275000 | 17.7353000 | -79.9500000 | -2.0333300 | -80.0833300 | 9.2083300 | -90.0000000 | 16.8333000 | -117.1316000 | 32.5713000 | -80.9160000 | -2.2500000 |  |  | -86.8300000 | 20.4800000 |
| -69.6500000 | 19.1300000 | -84.6100000 | 9.7600000 | -94.0305500 | 18.2083300 | -71.7500000 | 9.3333300 | -73.6452800 | 20.9365800 | -60.6000000 | -24.7000000 |  |  | -64.7444400 | 18.3383300 |
| -76.6160600 | 8.0013200 | -83.7333300 | 8.6166700 | -79.0666700 | 9.4583300 | -93.4000000 | 18.4200000 | -75.5517200 | 10.2536100 | -34.8486100 | -7.1375000 |  |  | -97.1603400 | 25.9975500 |
| -76.7176100 | 8.0826400 | -85.2350000 | 10.2546500 | -65.7748800 | 18.4091100 | -89.1666600 | 13.4833300 | -80.7160000 | -2.0500000 | -80.7500000 | 28.3000000 |  |  | -40.4254210 | -20.6019560 |
| -83.3916700 | 14.0208300 | -57.9666700 | 6.6666700 | -48.3294309 | -25.3107844 | -85.8833300 | 11.2666600 | -111.8925000 | 26.7513900 | -88.0500000 | 21.6000000 |  |  | -38.2833330 | -12.6000000 |
| -80.1583300 | 9.2000000 | -90.7200000 | 19.4000000 | -78.8333300 | 1.2833300 | -88.4000000 | 13.2000000 | -88.1161100 | 30.2775000 | -79.6597200 | 9.2513800 |  |  | -97.4361110 | 27.6958330 |
| -62.1333300 | 9.9166600 | -81.7300000 | 7.5900000 | -90.0000000 | 13.7000000 | -83.3333300 | 14.6500000 | -118.0000000 | 34.0000000 | -80.4950000 | 8.1950000 |  |  | -84.3702100 | 30.0167500 |
| -80.4666600 | -3.5000000 | -81.4400000 | 24.7183300 | -82.1780600 | 8.2513900 | -39.7019000 | -18.4033000 | -117.1516000 | 32.6585300 | -100.3600000 | 24.4500000 |  |  | -46.6168611 | -0.8355556 |
| -80.8492000 | 25.2255000 | -81.9861000 | 26.9972000 | -78.5833300 | 9.4416600 | -48.5558000 | -25.5917000 | -81.2416600 | 31.4775000 | -62.0666700 | 10.6500000 |  |  | -35.2000000 | -6.0833330 |
| -83.4354300 | 8.6498800 | -42.3494400 | -22.9263900 | -88.2005500 | 17.5144400 | -65.6394400 | 18.3569400 | -81.4900000 | 30.6247000 | -81.0000000 | -2.2000000 |  |  | -97.0652778 | 27.8183333 |
| -39.3983300 | -17.7980600 | -86.7500000 | 12.1666600 | -92.6800000 | 18.4800000 | -85.3400000 | 9.8300000 | -93.4188900 | 18.4297200 | -85.3400000 | 9.8300000 |  |  | -110.9835410 | 27.9618610 |
| -53.0500000 | 5.2833300 | -87.1333300 | 13.2333300 | -91.2386100 | 17.8575000 | -111.8670000 | 26.7833000 | -83.7666700 | 11.0000000 | -81.0847000 | 29.3489000 |  |  | -82.3061757 | 27.9312481 |
| -62.1333300 | 9.9166700 | -87.5500000 | 19.8200000 | -93.4977800 | 18.4136100 | -112.2934500 | 29.2244200 | -44.2242000 | -2.4844000 | -67.1906500 | 17.9393100 |  |  | -37.1369440 | -4.9561110 |
| -85.2100000 | 10.3500000 | -87.9725000 | 13.1600000 | -86.3388900 | 11.6416700 | -83.7666600 | 10.9666600 | -90.3300000 | 20.8300000 | -65.6257200 | 18.3649500 |  |  | -37.7591670 | -4.4927780 |
| -65.6527700 | 18.3277700 | -77.0300000 | 26.3700000 | -83.6530400 | 8.5283300 | -83.3000000 | 14.1333300 | -88.0605500 | 17.4886100 | -85.8819400 | 10.9019400 |  |  | -95.2102860 | 29.0130110 |
| -95.2400000 | 16.1686100 | -75.1000000 | 9.0666600 | -43.2720000 | -22.7659000 | -85.9083300 | 15.9583300 |  |  | -86.5166600 | 11.7833300 |  |  | -74.6235500 | 11.0159500 |
| -84.9269400 | 9.7766700 | -83.8833300 | 15.2666700 | -62.7666700 | 10.6166700 | -92.4500000 | 18.4600000 |  |  | -86.7333300 | 12.1500000 |  |  | -80.0500000 | 0.4330000 |
| -62.7666700 | 10.6166700 | -85.9814700 | 11.3476700 | -80.4200000 | -0.5000000 | -79.6666600 | -2.4500000 |  |  | -88.2188900 | 16.9555600 |  |  | -38.6000000 | -12.9500000 |
| -73.6452800 | 20.9365800 | -71.7200000 | 18.4700000 | -89.0752500 | 17.0571300 | -82.6035900 | 9.5917400 |  |  | -73.1471100 | 11.3701100 |  |  | -37.6536100 | -10.9948100 |
| -79.8330000 | 0.8500000 | -77.3604200 | 8.6636600 | -65.9119000 | 7.0758000 | -35.0140305 | -8.1166395 |  |  | -88.8665100 | 13.7369000 |  |  | -66.3465700 | 17.9756400 |
| -69.5900000 | 18.4500000 | -88.4333000 | 18.0500000 | -88.8000000 | 13.2500000 | -63.7000000 | 10.4500000 |  |  | -42.1891700 | -22.8502800 |  |  | -88.1225000 | 30.2572200 |
| -79.8833300 | -2.2333300 | -76.4500000 | 8.5333300 | -93.2800000 | 18.4200000 | -79.8330000 | 0.8500000 |  |  | -90.4000000 | 20.8500000 |  |  | -47.4000000 | -24.6500000 |
| -86.8333300 | 12.2583300 | -79.5702700 | 8.9372200 | -87.5900000 | 19.8000000 | -84.9100000 | 10.0400000 |  |  | -86.7200000 | 21.2100000 |  |  | -47.9298727 | -15.7834208 |
| -85.7855000 | 10.8420400 | -90.3333300 | 20.8333300 | -83.5916700 | 8.4805600 | -83.3300000 | 8.7300000 |  |  | -71.2275000 | 12.2813900 |  |  | -90.0200000 | 21.1500000 |
| -95.9883300 | 15.8122200 | -79.8691600 | 9.4027700 | -82.2333300 | 8.9972200 | -81.2384000 | 22.2485000 |  |  | -79.8330000 | 0.8500000 |  |  | -84.3008300 | 22.4397200 |
| -86.7333300 | 12.1500000 | -92.6405500 | 18.4225000 | -81.8700000 | 7.5100000 | -82.2700000 | 9.3800000 |  |  | -84.8300000 | 9.9700000 |  |  | -64.7008000 | 18.3103000 |
| -80.3500000 | 7.9666700 | -88.2833300 | 18.1833300 | -83.5900000 | 10.7900000 | -81.7008300 | 7.4094400 |  |  | -59.0666700 | -23.3666700 |  |  | -67.0502600 | 18.5139900 |
| -71.9853000 | 12.2492900 | -85.5336400 | 9.8846500 | -83.2800000 | 8.4000000 | -92.9777700 | 18.3397200 |  |  | -87.1666600 | 12.8500000 |  |  | -87.0045000 | 30.3586000 |
| -85.6633100 | 10.7801100 | -83.3355600 | 8.3986100 | -77.3556000 | 6.0130600 | -78.2250000 | 9.2833300 |  |  | -94.0366600 | 29.6763800 |  |  | -94.3800000 | 18.0900000 |
| -88.9494400 | 21.3827800 | -71.6300000 | 18.5300000 | -42.3494400 | -22.9263900 | -83.7600000 | 9.1800000 |  |  | -84.6666700 | 9.8666700 |  |  | -81.0000000 | -2.2000000 |
| -85.9083300 | 15.9583300 | -38.6081589 | -12.9642651 | -85.7833300 | 11.1500000 | -85.6890600 | 9.9834800 |  |  | -105.2500000 | 21.5333300 |  |  | -65.8075800 | 18.0642900 |
| -62.8333300 | 10.5500000 | -75.6956100 | 10.1360300 | -92.6616700 | 18.5216700 | -88.7000000 | 17.6000000 |  |  | -86.3000000 | 12.2500000 |  |  | -67.4805600 | 18.3842600 |
| -72.4166700 | 11.4166700 | -81.8300000 | 7.5900000 | -87.4333300 | 13.4166600 | -95.5600000 | 15.9900000 |  |  | -85.6300000 | 10.6800000 |  |  | -71.5300000 | 18.3000000 |
| -59.7738900 | 8.4030600 | -85.0700000 | 9.6600000 | -84.4600000 | 9.5300000 | -83.4355600 | 8.6497200 |  |  | -87.0045000 | 30.3586000 |  |  | -80.0833000 | -0.6667000 |
| -83.4963900 | 8.8269400 | -86.7200000 | 21.2100000 | -83.4091700 | 8.7333300 | -88.4333000 | 18.0500000 |  |  | -40.2816700 | -15.2569400 |  |  | -88.1666600 | 18.2500000 |
| -85.6194300 | 10.9242200 | -82.1780600 | 8.2513900 | -95.9661400 | 15.8205000 | -71.6500000 | 17.9166700 |  |  | -59.0000000 | -24.0000000 |  |  | -79.1660000 | 1.1000000 |
| -88.2216600 | 16.9680500 | -88.1700000 | 18.9000000 | -63.7000000 | 10.4500000 | -83.5916700 | 8.4805600 |  |  | -88.1500000 | 21.5600000 |  |  | -34.8486100 | -7.1375000 |
| -87.6666600 | 19.8333300 | -80.7500000 | -2.1000000 | -80.3166600 | 7.9000000 | -88.3641600 | 18.3494400 |  |  | -78.3166700 | -9.6666700 |  |  | -42.8184417 | -22.9232605 |
| -89.6927800 | 21.2563900 | -65.1322200 | 10.0661100 | -92.9552800 | 18.3527800 | -89.7300000 | 21.2600000 |  |  | -75.7000000 | 10.1333300 |  |  | -40.3500000 | -20.3500000 |
| -88.8050000 | 16.0975000 | -72.1500000 | 18.6666600 | -80.5333300 | 8.1583300 | -90.3300000 | 20.8800000 |  |  | -80.0000000 | -2.2000000 |  |  | -79.9497200 | 9.3705500 |
| -83.2926700 | 22.9530700 | -76.5125000 | -8.1841600 | -80.3317000 | 27.2600000 | -92.6800000 | 18.4800000 |  |  | -71.7300000 | 18.5700000 |  |  | -86.7333300 | 12.1500000 |
| -72.4988700 | 11.7412200 | -75.5798400 | 10.3346200 | -75.8427800 | 9.7936100 | -97.5300000 | 16.0000000 |  |  | -86.1750000 | 11.5250000 |  |  |  |  |
| -75.5121200 | 10.4533700 | -83.0000000 | 9.9200000 | -71.6666700 | 11.0000000 | -83.0800000 | 9.9900000 |  |  | -85.2188900 | 10.2859700 |  |  |  |  |
| -111.8670000 | 26.7833000 | -85.3722200 | 9.8444400 | -74.5366600 | 10.9858300 | -80.4666600 | -3.5000000 |  |  | -40.2733000 | -19.8203000 |  |  |  |  |
| -90.0000000 | 13.7000000 | -85.2500000 | 10.5000000 | -80.3697000 | 27.5867000 | -71.8333300 | 11.0333300 |  |  | -75.1387800 | 10.8420100 |  |  |  |  |
| -88.1955500 | 17.4841600 | -59.5002000 | 13.0639000 | -83.6666600 | 10.9333300 | -44.2500000 | -2.5000000 |  |  | -65.6527700 | 18.3277700 |  |  |  |  |
| -88.0605500 | 17.4886100 | -83.5833300 | 8.4666700 | -67.1615200 | 18.0079500 | -65.8200000 | 18.3700000 |  |  | -35.6913747 | -6.9668080 |  |  |  |  |
| -92.0333300 | 14.3902700 | -83.3666600 | 14.0833300 | -64.7644400 | 18.3213900 | -83.6033300 | 8.6166700 |  |  | -65.8802900 | 18.4248900 |  |  |  |  |
| -79.9666600 | -2.1666600 | -92.4200000 | 18.6400000 | -98.0300000 | 22.2200000 | -37.6000000 | -11.8000000 |  |  | -67.2000000 | 17.9700000 |  |  |  |  |
| -87.1666700 | 12.8500000 | -88.8665100 | 13.7369000 | -81.7300000 | 7.6100000 | -86.3388900 | 11.6416700 |  |  | -42.0183864 | -22.8832604 |  |  |  |  |
| -66.8876100 | 17.9586800 | -58.7017000 | 4.7364000 | -80.0300000 | 0.3700000 | -88.8600000 | 21.3900000 |  |  | -38.6000000 | -12.9500000 |  |  |  |  |
| -58.0000000 | 5.1667000 | -67.9377800 | 18.0891700 | -75.6086100 | 9.4717400 | -105.2833300 | 21.5500000 |  |  | -70.1666600 | -30.8000000 |  |  |  |  |
| -85.2500000 | 10.2666700 | -71.4000000 | 19.8700000 | -92.4563900 | 18.4719400 | -80.4841600 | 8.0113800 |  |  | -76.5000000 | 3.7500000 |  |  |  |  |
| -80.2160000 | -2.7830000 | -75.5517200 | 10.2536100 | -84.9000000 | 9.9500000 | -80.7175000 | 24.8622200 |  |  | -84.5572200 | 9.6336100 |  |  |  |  |
| -85.3400000 | 10.3400000 | -92.6000000 | 18.3500000 | -87.0388800 | 12.3777700 | -83.4200000 | 8.5200000 |  |  | -85.7159400 | 11.0872800 |  |  |  |  |
| -51.7825000 | -18.7575000 | -79.7125000 | 9.4666600 | -85.3500000 | 10.3500000 | -81.8364000 | 26.3917000 |  |  | -93.4000000 | 18.4200000 |  |  |  |  |
| -84.8333000 | 10.0000000 | -88.7500000 | 19.8700000 | -85.2350000 | 10.2546500 | -79.7333300 | -2.5000000 |  |  | -84.7333300 | 9.9416700 |  |  |  |  |
| -93.4000000 | 18.4200000 | -97.7700000 | 22.1200000 | -89.0100000 | 21.3700000 | -83.6000000 | 13.3500000 |  |  | -79.9333300 | -2.7333300 |  |  |  |  |
| -109.5486100 | 26.7061100 | -87.5833000 | 13.5833000 | -97.9500000 | 22.2200000 | -83.4833300 | 13.9250000 |  |  | -80.4475000 | 25.0861100 |  |  |  |  |
| -61.4333300 | 10.6166700 | -74.5000000 | 10.0000000 | -40.0800000 | -19.8300000 | -94.0305500 | 18.2083300 |  |  | -88.4248000 | 30.2062000 |  |  |  |  |
| -105.2500000 | 21.5333300 | -97.6389000 | 21.7603000 | -47.7000000 | -0.6200000 | -80.3333300 | -2.6833300 |  |  | -40.3333300 | -12.5000000 |  |  |  |  |
| -67.1468400 | 18.0563500 | -47.5800000 | -1.0166700 | -77.7291700 | 8.9416700 | -87.4333300 | 13.4166600 |  |  | -97.2700000 | 25.9500000 |  |  |  |  |
| -87.4975000 | 13.2988800 | -83.7500000 | 12.3333300 | -94.1155600 | 18.1677800 | -92.1713800 | 14.4925000 |  |  | -89.9550000 | 29.2630000 |  |  |  |  |
| -87.4416600 | 13.1925000 | -65.6527700 | 18.3277700 | -88.9500000 | 13.3500000 | -81.2500000 | 8.1667000 |  |  | -67.4806400 | 18.3843000 |  |  |  |  |
| -65.1322200 | 10.0661100 | -66.8625000 | 17.9708000 | -80.1916600 | 9.1500000 | -85.7855000 | 10.8420400 |  |  | -58.3910200 | -23.2362100 |  |  |  |  |
| -83.1800000 | 8.6300000 | -88.3180500 | 17.4433300 | -75.5076100 | 10.3346900 | -65.5613800 | 18.1147200 |  |  | -84.6700000 | 10.7900000 |  |  |  |  |
| -74.0803000 | 11.3156000 | -88.2058300 | 21.5552800 | -84.7300000 | 9.9600000 | -90.4400000 | 13.8941000 |  |  | -80.1500000 | -2.9330000 |  |  |  |  |
| -70.5583000 | 18.2200000 | -87.5800000 | 20.1200000 | -83.5666600 | 13.4000000 | -47.5761100 | -0.5847200 |  |  | -82.7000000 | 9.6300000 |  |  |  |  |
| -89.2450000 | 13.4844400 | -80.1583300 | 9.2000000 | -78.0291700 | 9.2291700 | -38.9333300 | -13.9500000 |  |  | -90.6800000 | 19.5800000 |  |  |  |  |
| -79.5894400 | 8.9930600 | -65.7666600 | 18.3833300 | -79.9013800 | 9.3591600 | -69.1000000 | 18.4200000 |  |  | -88.8884000 | 30.2381000 |  |  |  |  |
| -79.0833300 | 9.4500000 | -89.6600000 | 21.2600000 | -80.5333300 | 8.2666600 | -88.6666600 | 18.1166600 |  |  | -59.5147000 | 13.0494000 |  |  |  |  |
| -83.6722200 | 11.6722200 | -90.7538800 | 13.9330500 | -87.4416600 | 13.1925000 | -83.2125000 | 14.3083300 |  |  | -74.7518600 | 10.7000400 |  |  |  |  |
| -77.4666600 | 4.2500000 | -68.7300000 | 18.1300000 | -75.5978500 | 9.8114900 | -79.7530600 | 9.1683300 |  |  | -64.8000000 | -24.0666700 |  |  |  |  |
| -85.2188900 | 10.2859700 | -66.5196000 | 17.8944000 | -77.3555600 | 6.0463900 | -89.4527700 | 21.3180500 |  |  | -40.3378000 | -20.3194000 |  |  |  |  |
| -85.3194000 | 10.3235600 | -37.8916700 | -12.3833300 | -90.5000000 | 19.8700000 | -84.6327900 | 9.6173200 |  |  | -65.5886400 | 18.3869200 |  |  |  |  |
| -62.7500000 | 10.0833300 | -83.4000000 | 8.7388900 | -79.5555600 | 8.7930600 | -48.3420000 | -25.5090000 |  |  | -96.6667000 | 19.3333000 |  |  |  |  |
| -48.9761700 | -1.6192200 | -75.8427800 | 9.7936100 | -92.6300000 | 18.2700000 | -80.6097000 | 28.4169000 |  |  | -78.0291700 | 9.2291700 |  |  |  |  |
| -80.6099000 | 28.4171000 | -68.6181000 | 18.3645000 | -92.6136100 | 18.3725000 | -43.8327800 | -22.9100000 |  |  | -80.1333300 | 9.2083300 |  |  |  |  |
| -87.5141700 | 13.2747200 | -69.5000000 | 19.0800000 | -79.9586100 | 9.3650000 | -71.2000000 | 18.1700000 |  |  | -79.6000000 | 0.9500000 |  |  |  |  |
| -75.1000000 | 9.0666600 | -82.8219000 | 28.0236000 | -64.4000000 | 10.2833300 | -80.0300000 | 0.3700000 |  |  | -75.1333300 | 10.3600000 |  |  |  |  |
| -79.0000000 | 1.2160000 | -88.3680500 | 17.4641600 | -81.7063800 | 7.4088800 | -78.0291700 | 9.2291700 |  |  | -36.7897220 | -6.4119440 |  |  |  |  |
| -63.7666600 | 10.6500000 | -85.1166600 | 10.1766600 | -75.5000000 | 7.0000000 | -83.4333300 | 14.0166600 |  |  | -84.8038690 | 29.8902390 |  |  |  |  |
| -84.6965000 | 9.8676900 | -80.0894400 | 7.6772200 | -61.3557600 | 15.4356000 | -83.7055500 | 11.5972200 |  |  | -40.1430560 | -6.6052780 |  |  |  |  |
| -85.7000000 | 10.3000000 | -75.1666600 | 9.9666600 | -76.7224700 | 8.0779600 | -48.5800000 | -25.8800000 |  |  | -97.2958330 | 27.7055560 |  |  |  |  |
| -84.6300000 | 9.8000000 | -85.1604800 | 10.0334200 | -85.1610900 | 10.1077500 | -69.7700000 | 19.2700000 |  |  | -37.5313889 | -11.2716667 |  |  |  |  |
| -85.3400000 | 9.8300000 | -69.5900000 | 18.4500000 | -78.8351500 | -1.2862600 | -89.0100000 | 21.3700000 |  |  | -37.4141670 | -10.0369440 |  |  |  |  |
| -88.1611100 | 18.3163900 | -75.8525000 | 23.5580500 | -48.3200000 | -25.2700000 | -90.4300000 | 20.6800000 |  |  | -39.0666670 | -13.3666670 |  |  |  |  |
| -71.0750000 | 18.1700000 | -88.3641600 | 18.3494400 | -86.8830500 | 20.8508300 | -65.8333300 | 18.4166600 |  |  | -95.0767280 | 29.7512610 |  |  |  |  |
| -39.0310900 | -14.4724700 | -87.9666700 | 18.9733300 | -77.9333300 | 7.9916600 | -83.2955600 | 8.5352800 |  |  | -41.4178611 | -2.9109167 |  |  |  |  |
| -47.6833300 | -0.6166700 | -65.4452700 | 18.1494400 | -83.5916700 | 10.7944400 | -82.1780600 | 8.2513900 |  |  | -39.0833330 | -12.5333330 |  |  |  |  |
| -88.2005500 | 17.5144400 | -90.8236100 | 13.9200000 | -58.1833300 | 6.6666600 | -88.0605500 | 17.4886100 |  |  | -37.9400000 | -9.5572222 |  |  |  |  |
| -93.2800000 | 18.4200000 | -95.9658300 | 15.8240600 | -71.1000000 | 18.2000000 | -79.7500000 | 9.4450000 |  |  | -92.2700500 | 14.8890500 |  |  |  |  |
| -88.2408300 | 17.5325000 | -87.9391600 | 13.1600000 | -97.6389000 | 21.7603000 | -91.1200000 | 18.5400000 |  |  | -96.1229220 | 19.1421280 |  |  |  |  |
| -57.9166600 | 6.6333300 | -83.3102800 | 8.5305600 | -85.6667000 | 15.7500000 | -85.8189300 | 10.9223200 |  |  | -38.5000000 | -12.2608330 |  |  |  |  |
| -62.7000000 | 10.1166700 | -68.8500000 | 18.9667000 | -86.2161100 | 11.5827700 | -48.7122581 | -25.4324846 |  |  | -39.6575000 | -15.1533330 |  |  |  |  |
| -57.7800000 | 6.5833300 | -57.9200000 | 6.6300000 | -92.6213800 | 18.2794400 | -67.8921000 | 18.0866900 |  |  | -60.5833330 | -29.6666670 |  |  |  |  |
| -84.1600000 | 9.4200000 | -71.5000000 | 18.3700000 | -84.9333300 | 9.7833300 | -48.9297500 | -25.0192500 |  |  | -40.4592500 | -2.8731940 |  |  |  |  |
| -50.7600000 | -0.1100000 | -80.0500000 | 0.0333300 | -92.1713900 | 14.4925000 | -76.7228000 | 8.0988200 |  |  | -93.8677800 | 16.0616700 |  |  |  |  |
| -85.6791600 | 10.5744400 | -76.4333300 | 8.0833300 | -76.4500000 | 8.5333300 | -65.8895500 | 18.4294000 |  |  | -80.7500000 | -2.1166600 |  |  |  |  |
| -87.4500000 | 13.4166600 | -86.9166700 | 16.1166700 | -77.3137100 | 5.6573400 | -66.6572500 | 17.9889100 |  |  | -79.5525000 | 8.8002700 |  |  |  |  |
| -77.4333300 | 6.1333300 | -78.9186100 | 8.3666700 | -61.4695000 | 15.5872000 | -92.4600000 | 18.6300000 |  |  | -66.0769400 | -30.0461100 |  |  |  |  |
| -69.1000000 | 12.2666700 | -85.6900000 | 10.5500000 | -81.9839000 | 26.9992000 | -105.2500000 | 21.5333300 |  |  | -85.9781000 | 11.3461000 |  |  |  |  |
| -74.1641900 | 11.2959200 | -88.2408300 | 17.5325000 | -67.9377800 | 18.0891700 | -39.1000000 | -16.4500000 |  |  | -88.1200000 | 21.5700000 |  |  |  |  |
| -77.6693800 | 2.4203400 | -85.6472200 | 9.9111100 | -76.8160000 | 18.2833000 | -84.1600000 | 9.4500000 |  |  | -43.2076686 | -22.9069598 |  |  |  |  |
| -81.3717000 | 30.1103000 | -88.1958300 | 17.4844400 | -57.9166600 | 6.6333300 | -38.0666700 | -12.3500000 |  |  | -92.9727700 | 31.7547200 |  |  |  |  |
| -66.6469400 | 17.9841700 | -39.0000000 | -14.1000000 | -83.7333300 | 8.6166700 | -48.5269400 | -27.6577800 |  |  | -80.4833300 | 8.0166700 |  |  |  |  |
| -80.0500000 | 0.0333300 | -37.6000000 | -11.7500000 | -88.9319400 | 15.7655600 | -66.9038700 | 17.9579000 |  |  | -80.8294000 | 28.7839000 |  |  |  |  |
| -87.6469400 | 20.0502700 | -71.6700000 | 18.9200000 | -62.1333300 | 9.9166700 | -34.8727216 | -7.9443347 |  |  | -59.6666700 | -23.0500000 |  |  |  |  |
| -86.9633300 | 20.7302700 | -88.2166600 | 17.5000000 | -64.8400000 | 10.0700000 | -68.6417000 | 18.2200000 |  |  | -71.0750000 | 18.1700000 |  |  |  |  |
| -80.5333300 | 8.1583300 | -85.6452700 | 10.9369400 | -79.5894400 | 8.9930600 | -90.3300000 | 20.8300000 |  |  | -65.7666600 | 18.3833300 |  |  |  |  |
| -83.3083300 | 8.5416700 | -87.0430500 | 20.6802700 | -84.7597200 | 9.9977700 | -87.5900000 | 19.8000000 |  |  | -40.5180838 | -11.1844226 |  |  |  |  |
| -84.6555600 | 9.7222200 | -79.6666600 | -2.4500000 | -77.7333300 | 7.7333300 | -75.8427800 | 9.7936100 |  |  | -59.9666700 | -22.3000000 |  |  |  |  |
| -85.2031600 | 10.1852700 | -89.2450000 | 13.4844400 | -79.6666600 | -2.4500000 | -88.2408300 | 17.5325000 |  |  | -80.4160000 | -0.5000000 |  |  |  |  |
| -67.0453400 | 17.9907500 | -85.7972200 | 10.5055600 | -85.2694400 | 10.1138900 | -83.8166600 | 10.9166600 |  |  | -84.6600000 | 9.8600000 |  |  |  |  |
| -67.2000000 | 17.9700000 | -84.3008300 | 22.4397200 | -85.7333300 | 10.9347200 | -72.1667000 | 10.0000000 |  |  | -75.0000000 | 10.7500000 |  |  |  |  |
| -62.7833300 | 10.0333300 | -88.0297000 | 17.7378000 | -84.3008300 | 22.4397200 | -90.3861100 | 20.8583300 |  |  | -79.9502700 | 9.3416600 |  |  |  |  |
| -75.2534700 | 10.7520600 | -68.8000000 | 18.3250000 | -48.5097444 | -25.5241867 | -86.8833300 | 12.2833300 |  |  | -62.8500000 | -28.4500000 |  |  |  |  |
| -75.6903600 | 9.3936000 | -66.8481200 | 17.9564400 | -70.5500000 | 19.7500000 | -93.3300000 | 15.6000000 |  |  | -88.0264000 | 17.7367000 |  |  |  |  |
| -77.2833300 | 3.5666700 | -82.4139000 | 27.7290000 | -75.5022400 | 10.3015100 | -93.8677800 | 16.0616700 |  |  | -39.0000000 | -14.1000000 |  |  |  |  |
| -85.8833300 | 11.2666600 | -81.3541700 | 24.6694400 | -83.4800000 | 8.7000000 | -75.7000000 | 10.1333300 |  |  | -74.1897900 | 10.9873000 |  |  |  |  |
| -79.8786100 | 9.3713900 | -89.0100000 | 21.3700000 | -39.1000000 | -16.4500000 | -84.7194400 | 9.9055600 |  |  | -83.4000000 | 14.0166600 |  |  |  |  |
| -110.4666700 | 24.1833300 | -86.5166600 | 11.7833300 | -43.2075000 | -22.9028000 | -65.4235500 | 18.1295500 |  |  | -79.7500000 | 9.4450000 |  |  |  |  |
| -105.0333300 | 19.4500000 | -79.1069400 | 8.3988800 | -80.7500000 | -2.1000000 | -88.0269000 | 17.7353000 |  |  | -90.1100000 | 19.0400000 |  |  |  |  |
| -85.2333300 | 10.8111100 | -83.3083300 | 8.5416700 | -88.0605500 | 17.4886100 | -43.2076686 | -22.9069598 |  |  | -86.7500000 | 12.1666600 |  |  |  |  |
| -85.6662500 | 11.0477700 | -87.4316700 | 13.1727800 | -84.8405500 | 9.9763800 | -46.1386733 | -23.8582696 |  |  | -68.6000000 | 18.3800000 |  |  |  |  |
| -52.3166700 | 4.9333300 | -90.3800000 | 19.9000000 | -62.0666600 | 10.6500000 | -71.6666700 | 11.0000000 |  |  | -71.6300000 | 18.5300000 |  |  |  |  |
| -69.2800000 | 18.4700000 | -77.1983600 | 8.4091700 | -85.8356400 | 10.9392500 | -62.7666700 | 10.6166700 |  |  | -86.5783000 | 30.3961000 |  |  |  |  |
| -89.9781600 | 29.2555400 | -75.3504400 | 9.5009200 | -111.8925000 | 26.7513900 | -84.6900000 | 9.8600000 |  |  | -65.3316700 | 18.3110900 |  |  |  |  |
| -85.6883300 | 10.9761100 | -88.6166600 | 16.7500000 | -89.2666600 | 13.4833300 | -76.9353000 | 8.0112400 |  |  | -83.5900000 | 8.4800000 |  |  |  |  |
| -85.8444400 | 10.3000000 | -93.8219400 | 15.9466700 | -72.5000000 | 11.5000000 | -82.0000000 | 27.0333000 |  |  | -80.0000000 | -3.2666600 |  |  |  |  |
| -85.2171400 | 10.3092500 | -78.0291700 | 9.2291700 | -74.7739900 | 11.0374600 | -44.0408262 | -22.9635597 |  |  | -85.7644400 | 10.9043400 |  |  |  |  |
| -85.3548900 | 10.3156500 | -83.2125000 | 14.3083300 | -92.1211100 | 14.4588800 | -65.7666700 | 18.3833300 |  |  | -68.9500000 | 12.1166700 |  |  |  |  |
| -84.8280600 | 10.0008300 | -87.4000000 | 12.7500000 | -79.5705500 | 8.9686100 | -83.4800000 | 8.7000000 |  |  | -59.0000000 | -21.7500000 |  |  |  |  |
| -90.5000000 | 19.8700000 | -73.4175000 | -3.9694400 | -83.4354300 | 8.6498800 | -78.7677800 | 1.1158300 |  |  | -85.5336400 | 9.8846500 |  |  |  |  |
| -84.7100000 | 9.9300000 | -85.7855000 | 10.8420400 | -85.6555700 | 10.7916700 | -94.1000000 | 18.1600000 |  |  | -88.3058300 | 30.3794400 |  |  |  |  |
| -61.7899000 | 17.1147700 | -75.5750000 | 10.2317400 | -76.7445600 | 8.0923300 | -86.9719400 | 12.3280600 |  |  | -105.1666600 | 21.4333300 |  |  |  |  |
| -61.8896200 | 17.0554600 | -75.5076100 | 10.3346900 | -83.1800000 | 8.6300000 | -85.1587400 | 10.1749500 |  |  | -83.2055600 | 8.7027800 |  |  |  |  |
| -61.8578800 | 17.0190600 | -82.5161000 | 27.2183000 | -84.6200000 | 9.7700000 | -113.5120000 | 29.0669000 |  |  | -85.7489800 | 11.0284200 |  |  |  |  |
| -81.2956200 | 19.2960100 | -86.7666600 | 12.2000000 | -81.2444000 | 29.7253000 | -78.5636100 | 1.7333300 |  |  |  |  |  |  |  |  |
| -81.3197600 | 19.2896100 | -83.5666600 | 13.4000000 | -38.6000000 | -12.9500000 | -70.5500000 | 19.7500000 |  |  |  |  |  |  |  |  |
| -81.2711900 | 19.3711100 | -89.0000000 | 13.3500000 | -76.5000000 | 3.7500000 | -75.1000000 | 9.0666600 |  |  |  |  |  |  |  |  |
| -81.0857400 | 19.3351700 | -85.7489800 | 11.0284200 | -87.5141600 | 13.2747200 | -74.1500000 | 11.2666700 |  |  |  |  |  |  |  |  |
| -39.7818950 | -18.7572240 | -76.4166600 | -2.9166600 | -90.7538800 | 13.9330500 | -88.8758300 | 15.7644400 |  |  |  |  |  |  |  |  |
| -49.6095110 | -0.0568780 | -87.4500000 | 13.4166600 | -66.0551600 | 18.3916100 | -81.7300000 | 7.6100000 |  |  |  |  |  |  |  |  |
| -80.4143210 | 25.1126310 | -38.9965836 | -14.2814954 | -67.0808000 | 18.5133000 | -81.2954600 | 19.2979900 |  |  |  |  |  |  |  |  |
| -111.9069780 | 26.7268580 | -65.3169000 | 18.3289000 | -77.3613800 | 8.6301900 | -61.7898300 | 17.1148500 |  |  |  |  |  |  |  |  |
| -80.1314670 | 26.0530970 | -67.4806400 | 18.3843000 | -75.5029700 | 10.2308000 | -77.0412000 | 17.8563600 |  |  |  |  |  |  |  |  |
| -82.7365336 | 29.3236005 | -70.7000000 | 18.4166700 | -82.6000000 | 9.5900000 | -82.6790000 | 27.7710000 |  |  |  |  |  |  |  |  |
| -39.0605560 | -14.7955560 | -69.1000000 | 18.4000000 | -74.0803000 | 11.3156000 | -81.4070000 | 24.6850000 |  |  |  |  |  |  |  |  |
| -81.7670670 | 24.5527000 | -71.6500000 | 17.9800000 | -77.3531000 | 4.9563900 | -80.6340000 | 25.5550000 |  |  |  |  |  |  |  |  |
| -111.0386450 | 27.9610410 | -70.0000000 | 18.4500000 | -34.9075000 | -7.2600000 | -80.9180000 | 25.1620000 |  |  |  |  |  |  |  |  |
| -81.1617943 | 29.0460339 | -75.2500000 | 10.7500000 | -62.1333300 | 9.9166600 | -81.3780000 | 29.8190000 |  |  |  |  |  |  |  |  |
| -90.7173200 | 29.0517160 | -78.4166700 | 8.0666700 | -79.6280500 | 8.8894400 | -82.0790000 | 26.5070000 |  |  |  |  |  |  |  |  |
| -34.8630560 | -7.1150000 | -89.9897200 | 21.1130600 | -85.9083300 | 15.9583300 | -80.1490000 | 27.1240000 |  |  |  |  |  |  |  |  |
| -35.0576670 | -8.5848610 | -97.5333300 | 16.0000000 | -81.1269400 | 7.9072200 | -80.7700000 | 25.1750000 |  |  |  |  |  |  |  |  |
| -37.2063890 | -11.0147220 | -79.9233300 | 9.2705600 | -92.6294400 | 18.3741600 | -80.8010000 | 25.2820000 |  |  |  |  |  |  |  |  |
| -46.5122222 | -1.0333333 | -84.8280600 | 10.0008300 | -89.1666600 | 13.4833300 | -80.5060000 | 25.0300000 |  |  |  |  |  |  |  |  |
| -92.9700000 | 18.3800000 | -96.6667000 | 19.3333000 | -88.3683300 | 16.5158300 | -80.4090000 | 25.1240000 |  |  |  |  |  |  |  |  |
| -79.8666700 | 0.8000000 | -38.0666700 | -12.3500000 | -83.3083300 | 8.5416700 | -80.1950000 | 26.1330000 |  |  |  |  |  |  |  |  |
| -85.1586100 | 10.1750000 | -44.1819400 | -2.4655600 | -82.8219000 | 28.0236000 | -82.5750000 | 27.5920000 |  |  |  |  |  |  |  |  |
| -83.3333300 | 14.1000000 | -78.9886100 | 22.6530600 | -83.3102800 | 8.5305600 | -81.7680000 | 24.5530000 |  |  |  |  |  |  |  |  |
| -78.1513800 | 8.4205500 | -83.4622100 | 8.6871900 | -83.0956800 | 9.9986900 | -80.1180000 | 26.0380000 |  |  |  |  |  |  |  |  |
| -75.7000000 | 10.1333300 | -83.4333300 | 14.0166600 | -52.3000000 | 4.8333300 | -80.7560000 | 28.8220000 |  |  |  |  |  |  |  |  |
| -75.8525000 | 23.5580500 | -82.2633300 | 9.2350000 | -75.5750000 | 10.2466700 | -81.0699000 | 24.7115000 |  |  |  |  |  |  |  |  |
| -73.1471100 | 11.3701100 | -57.7833300 | 6.5833300 | -96.6667000 | 19.3333000 | -80.6569000 | 24.9003000 |  |  |  |  |  |  |  |  |
| -87.6666700 | 19.8333300 | -78.2855100 | 2.6548000 | -87.4833300 | 12.9500000 | -97.2647000 | 27.5159000 |  |  |  |  |  |  |  |  |
| -89.1666600 | 13.4833300 | -75.2522200 | 10.2847200 | -86.9666600 | 12.3333300 | -38.4412640 | -3.7800500 |  |  |  |  |  |  |  |  |
| -86.1666600 | 11.5166600 | -83.4354300 | 8.6498800 | -65.8333300 | 18.4166600 | -48.3907500 | -25.5505280 |  |  |  |  |  |  |  |  |
| -87.1500000 | 12.8166700 | -85.6277800 | 10.8375000 | -85.1604800 | 10.0334200 | -37.1461110 | -10.8422220 |  |  |  |  |  |  |  |  |
| -81.0892000 | 29.3503000 | -93.8677800 | 16.0616700 | -81.2954600 | 19.2979900 | -47.9500000 | -24.9847220 |  |  |  |  |  |  |  |  |
| -65.5913400 | 18.2520100 | -79.7261100 | 8.6316700 | -61.7388400 | 12.0146700 | -96.3777800 | 19.5916700 |  |  |  |  |  |  |  |  |
| -90.5288800 | 14.6219400 | -88.1611100 | 18.3163900 | -61.7898300 | 17.1148500 | -37.5833330 | -11.8833330 |  |  |  |  |  |  |  |  |
| -85.2611100 | 10.3097200 | -76.0845300 | 9.3311300 | -61.8581100 | 17.0192000 | -39.0564720 | -3.4129720 |  |  |  |  |  |  |  |  |
| -84.9013900 | 9.9361100 | -38.5108330 | -12.9713890 | -77.0412000 | 17.8563600 | -34.9786330 | -6.4936170 |  |  |  |  |  |  |  |  |
| -89.0000000 | 13.3500000 | -37.7666670 | -4.7794440 | -80.7740000 | 28.6660000 | -82.0467000 | 26.7428170 |  |  |  |  |  |  |  |  |
| -79.5719400 | 8.9305600 | -56.0761110 | -2.2247220 | -82.6790000 | 27.7710000 | -38.9633330 | -14.0766670 |  |  |  |  |  |  |  |  |
| -75.2666600 | 10.1333300 | -47.5745500 | -0.5887360 | -81.4400000 | 24.7180000 | -79.8975000 | -2.2038889 |  |  |  |  |  |  |  |  |
| -71.9031700 | 12.1968400 | -37.1434170 | -10.8396110 | -80.4470000 | 25.0860000 | -80.4444210 | 25.2877370 |  |  |  |  |  |  |  |  |
| -41.7869000 | -22.3708000 | -46.6266667 | -0.8766667 | -80.8490000 | 25.2260000 | -47.8376390 | -24.8902220 |  |  |  |  |  |  |  |  |
| -85.6083300 | 10.8750000 | -68.2771110 | 10.9342820 | -80.6100000 | 28.4170000 | -38.4527780 | -12.8005560 |  |  |  |  |  |  |  |  |
| -92.1713800 | 14.4925000 | -40.1555560 | -19.9538890 | -82.0000000 | 27.0330000 | -50.0089722 | 1.6923611 |  |  |  |  |  |  |  |  |
| -37.6000000 | -11.8000000 | -37.2063890 | -11.0147220 | -81.8360000 | 26.3920000 | -46.6000000 | -0.8333330 |  |  |  |  |  |  |  |  |
| -75.2500000 | 10.7500000 | -87.7166670 | 18.7166670 | -81.4070000 | 24.6850000 | -37.0716670 | -10.9111110 |  |  |  |  |  |  |  |  |
| -84.4277800 | 9.5194400 | -80.0567000 | 26.8822000 | -80.6340000 | 25.5550000 | -81.0439298 | 25.5225162 |  |  |  |  |  |  |  |  |
| -85.2671600 | 10.1418200 | -91.0094440 | 18.1333330 | -80.0410000 | 26.5860000 | -48.5091670 | -25.5211110 |  |  |  |  |  |  |  |  |
| -84.1600000 | 9.4100000 | -37.1964170 | -11.1860560 | -80.9180000 | 25.1620000 | -92.9600000 | 18.3300000 |  |  |  |  |  |  |  |  |
| -80.3688800 | 7.9575000 | -104.9166600 | 19.3166600 | -81.3780000 | 29.8190000 | -83.6880600 | 12.3394400 |  |  |  |  |  |  |  |  |
| -83.7600000 | 9.1800000 | -105.2833300 | 21.5500000 | -82.0790000 | 26.5070000 | -81.4400000 | 24.7183300 |  |  |  |  |  |  |  |  |
| -76.2175700 | 8.9406600 | -85.7833300 | 11.1333300 | -80.1490000 | 27.1240000 | -80.4472200 | 25.0863800 |  |  |  |  |  |  |  |  |
| -90.1945100 | 29.4057800 | -75.6510900 | 9.4038600 | -80.7700000 | 25.1750000 | -84.1166600 | 11.2666600 |  |  |  |  |  |  |  |  |
| -93.2000000 | 18.4100000 | -64.7061100 | 18.3463900 | -80.8010000 | 25.2820000 | -105.0772200 | 19.5133300 |  |  |  |  |  |  |  |  |
| -74.5865300 | 11.0067300 | -87.7922200 | 13.4355600 | -80.9420000 | 25.3630000 | -88.1167000 | 18.3500000 |  |  |  |  |  |  |  |  |
| -86.7500000 | 12.1666600 | -88.0583300 | 17.3797200 | -80.5060000 | 25.0300000 | -87.6286100 | 15.9125000 |  |  |  |  |  |  |  |  |
| -86.8700000 | 20.9400000 | -88.4000000 | 17.4500000 | -80.4090000 | 25.1240000 | -80.0414000 | 26.5858000 |  |  |  |  |  |  |  |  |
| -93.8677800 | 16.0616700 | -67.0808000 | 18.5130000 | -80.7500000 | 25.2960000 | -77.6750000 | 8.8083300 |  |  |  |  |  |  |  |  |
| -90.2041600 | 13.7808300 | -65.1638800 | 9.9444400 | -80.1190000 | 26.9070000 | -81.8563800 | 7.5236100 |  |  |  |  |  |  |  |  |
| -65.8200000 | 18.3700000 | -74.7500000 | -8.6666600 | -80.1950000 | 26.1330000 | -79.5666600 | 8.9333300 |  |  |  |  |  |  |  |  |
| -92.8108300 | 15.2100000 | -84.9000000 | 9.8166700 | -82.5750000 | 27.5920000 | -78.8330000 | 1.2330000 |  |  |  |  |  |  |  |  |
| -84.9500000 | 10.0600000 | -88.7800000 | 19.8900000 | -81.3830000 | 25.8430000 | -85.6166600 | 10.8333300 |  |  |  |  |  |  |  |  |
| -83.5900000 | 8.4800000 | -86.7000000 | 12.0500000 | -81.7680000 | 24.5530000 | -80.2160000 | -2.7330000 |  |  |  |  |  |  |  |  |
| -81.9861000 | 26.9972000 | -85.9083300 | 10.9083300 | -80.1180000 | 26.0380000 | -78.8077800 | 1.6922200 |  |  |  |  |  |  |  |  |
| -91.7500000 | 18.3500000 | -81.7500000 | 7.6300000 | -81.0699000 | 24.7115000 | -91.8666700 | 18.3500000 |  |  |  |  |  |  |  |  |
| -83.3102800 | 8.5305600 | -109.5700000 | 26.6917000 | -80.0425000 | 26.6750000 | -34.9075000 | -7.2600000 |  |  |  |  |  |  |  |  |
| -78.0291700 | 9.2291700 | -87.5141700 | 13.2747200 | -81.3197900 | 19.2896200 | -86.1666600 | 11.5333300 |  |  |  |  |  |  |  |  |
| -71.9640500 | 12.2540800 | -88.0605500 | 17.4886100 | -81.2625000 | 19.3649500 | -85.8600000 | 10.9100000 |  |  |  |  |  |  |  |  |
| -86.8200000 | 21.0300000 | -89.1666600 | 13.4833300 | -61.7739360 | 12.0160900 | -92.6300000 | 18.2600000 |  |  |  |  |  |  |  |  |
| -81.3541700 | 24.6694400 | -83.7263900 | 9.1513900 | -61.8580700 | 17.1424000 | -82.2888800 | 9.1958300 |  |  |  |  |  |  |  |  |
| -76.7521200 | 8.3333600 | -85.6730200 | 9.9474900 | -77.0755200 | 17.8879900 | -77.4011500 | 6.2225500 |  |  |  |  |  |  |  |  |
| -39.8000000 | -17.5000000 | -75.2415400 | 10.7452800 | -38.7500000 | -12.8666670 | -60.3666600 | 8.5833300 |  |  |  |  |  |  |  |  |
| -79.0500000 | 1.1666600 | -74.0803000 | 11.3156000 | -37.1547220 | -4.9797220 | -89.2461100 | 13.4672200 |  |  |  |  |  |  |  |  |
| -79.6666600 | -2.4500000 | -80.1186100 | 7.6600000 | -38.4458330 | -12.7741670 | -87.6000000 | 15.8333300 |  |  |  |  |  |  |  |  |
| -80.4475000 | 25.0861100 | -85.2500000 | 10.2666600 | -38.9500000 | -12.6166670 | -85.7445300 | 10.9028900 |  |  |  |  |  |  |  |  |
| -83.8500000 | 9.2416700 | -83.5916700 | 8.4805600 | -48.5533330 | -27.1583330 | -80.7160000 | -2.0500000 |  |  |  |  |  |  |  |  |
| -61.8543200 | 17.1444600 | -68.3700000 | 18.5250000 | -35.0641670 | -8.3994440 | -67.1989900 | 17.9567900 |  |  |  |  |  |  |  |  |
| -77.0812300 | 17.8863700 | -80.4472200 | 25.0863800 | -35.2097220 | -5.7950000 | -84.4277800 | 9.5188900 |  |  |  |  |  |  |  |  |
| -81.1935800 | 19.2966900 | -89.9872200 | 20.1800000 | -48.6985280 | -26.3376110 | -81.9839000 | 26.9992000 |  |  |  |  |  |  |  |  |
|  |  | -83.4000000 | 14.0166600 | -40.4197220 | -20.2641670 | -47.8375000 | -0.6750000 |  |  |  |  |  |  |  |  |
|  |  | -52.3300000 | 4.5000000 | -37.3833330 | -11.4500000 | -85.1990300 | 10.0636000 |  |  |  |  |  |  |  |  |
|  |  | -88.3700000 | 16.5200000 | -40.3375000 | -20.3197220 | -87.0000000 | 20.3100000 |  |  |  |  |  |  |  |  |
|  |  | -75.4833300 | 10.5500000 | -48.6277780 | -26.8225000 | -90.4100000 | 20.6000000 |  |  |  |  |  |  |  |  |
|  |  | -75.5227400 | 10.2760300 | -37.5363610 | -11.4165560 | -67.1786100 | 17.9973500 |  |  |  |  |  |  |  |  |
|  |  | -83.6794400 | 12.3366700 | -34.8630560 | -7.1150000 | -79.0666700 | 9.4583300 |  |  |  |  |  |  |  |  |
|  |  | -65.6394400 | 18.3569400 | -47.9166670 | -25.0166670 | -79.5894400 | 8.9930600 |  |  |  |  |  |  |  |  |
|  |  | -72.8400000 | 19.5300000 | -35.8355560 | -7.6727780 | -84.7811100 | 9.9861100 |  |  |  |  |  |  |  |  |
|  |  | -66.6572500 | 17.9889100 | -39.0666670 | -13.3666670 | -71.9666600 | 12.0833300 |  |  |  |  |  |  |  |  |
|  |  | -39.0751883 | -15.2970178 | -66.0166670 | 18.4333330 | -85.1610900 | 10.1077500 |  |  |  |  |  |  |  |  |
|  |  | -75.1387800 | 10.8420100 | -47.3969444 | -0.9686111 | -80.7739000 | 28.6660000 |  |  |  |  |  |  |  |  |
|  |  | -80.6833000 | 28.3706000 | -95.7652650 | 18.3644490 | -92.6000000 | 18.3500000 |  |  |  |  |  |  |  |  |
|  |  | -80.0019400 | 7.5286100 | -40.3041670 | -20.2766670 | -67.9377800 | 18.0891700 |  |  |  |  |  |  |  |  |
|  |  | -88.2558300 | 18.6116700 | -80.2683333 | 25.6750000 | -83.7750000 | 11.5041600 |  |  |  |  |  |  |  |  |
|  |  | -83.2906400 | 8.5233900 | -48.5173330 | -27.5775000 | -87.4333300 | 13.2100000 |  |  |  |  |  |  |  |  |
|  |  | -85.6891700 | 10.9761100 | -37.4483330 | -11.3508330 | -80.1333300 | -2.9166600 |  |  |  |  |  |  |  |  |
|  |  | -65.5884200 | 18.3866900 | -47.9228470 | -25.0728890 | -82.3500000 | 9.3000000 |  |  |  |  |  |  |  |  |
|  |  | -75.0000000 | 10.7500000 | -37.7833330 | -11.5500000 | -94.0805500 | 18.0980500 |  |  |  |  |  |  |  |  |
|  |  | -86.3388800 | 11.6416600 | -92.8483300 | 18.2472200 | -61.6102800 | 12.2189400 |  |  |  |  |  |  |  |  |
|  |  | -71.8333300 | 11.0333300 | -85.3500000 | 10.3300000 | -61.7388400 | 12.0146700 |  |  |  |  |  |  |  |  |
|  |  | -85.1000000 | 9.5800000 | -80.3069400 | 9.1552700 | -61.8581100 | 17.0192000 |  |  |  |  |  |  |  |  |
|  |  | -88.2005600 | 17.5144400 | -79.7125000 | 9.4666600 | -80.9420000 | 25.3630000 |  |  |  |  |  |  |  |  |
|  |  | -74.1641900 | 11.2959200 | -77.4666600 | 4.2500000 | -82.5030000 | 27.7020000 |  |  |  |  |  |  |  |  |
|  |  | -72.8333300 | -3.3666700 | -58.1833300 | 6.6666700 | -80.7500000 | 25.2960000 |  |  |  |  |  |  |  |  |
|  |  | -87.7180500 | 19.7280500 | -90.0936100 | 21.1555500 | -80.1190000 | 26.9070000 |  |  |  |  |  |  |  |  |
|  |  | -82.9100000 | 8.1500000 | -79.7333300 | -2.5000000 | -81.3830000 | 25.8430000 |  |  |  |  |  |  |  |  |
|  |  | -69.2800000 | 18.4700000 | -38.0666700 | -12.3500000 | -80.6050000 | 28.0740000 |  |  |  |  |  |  |  |  |
|  |  | -78.2000000 | 8.4500000 | -79.5694400 | 8.9250000 | -80.0425000 | 26.6750000 |  |  |  |  |  |  |  |  |
|  |  | -92.6213800 | 18.2794400 | -93.8677800 | 16.0616700 |  |  |  |  |  |  |  |  |  |  |
|  |  | -89.2666700 | 21.3333300 | -84.8333000 | 10.0000000 |  |  |  |  |  |  |  |  |  |  |
|  |  | -79.6280500 | 8.8894400 | -75.6510900 | 9.4038600 |  |  |  |  |  |  |  |  |  |  |
|  |  | -82.5758400 | 9.5771900 | -75.6956100 | 10.1360300 |  |  |  |  |  |  |  |  |  |  |
|  |  | -76.4333300 | 8.8500000 | -71.2865800 | 11.9199200 |  |  |  |  |  |  |  |  |  |  |
|  |  | -95.5666600 | 15.9666600 | -75.3914500 | 24.2480500 |  |  |  |  |  |  |  |  |  |  |
|  |  | -82.6035900 | 9.5917400 | -59.5767000 | 13.0752000 |  |  |  |  |  |  |  |  |  |  |
|  |  | -37.6000000 | -11.8000000 | -85.6791600 | 10.5744400 |  |  |  |  |  |  |  |  |  |  |
|  |  | -85.2031600 | 10.1852700 | -67.2000000 | 17.9583300 |  |  |  |  |  |  |  |  |  |  |
|  |  |  |  | -84.1441100 | 9.3864200 |  |  |  |  |  |  |  |  |  |  |
|  |  |  |  | -86.7333300 | 12.1500000 |  |  |  |  |  |  |  |  |  |  |
|  |  |  |  | -77.4333300 | 6.1333300 |  |  |  |  |  |  |  |  |  |  |
|  |  |  |  | -75.1666600 | 9.9666600 |  |  |  |  |  |  |  |  |  |  |
|  |  |  |  | -65.2895400 | 18.2937400 |  |  |  |  |  |  |  |  |  |  |
|  |  |  |  | -84.6900000 | 9.8600000 |  |  |  |  |  |  |  |  |  |  |
|  |  |  |  | -69.6500000 | 19.1300000 |  |  |  |  |  |  |  |  |  |  |
|  |  |  |  | -38.9469400 | -15.6666700 |  |  |  |  |  |  |  |  |  |  |
|  |  |  |  | -77.7666700 | 8.0833300 |  |  |  |  |  |  |  |  |  |  |
|  |  |  |  | -83.7000000 | 8.6000000 |  |  |  |  |  |  |  |  |  |  |
|  |  |  |  | -84.1600000 | 9.4200000 |  |  |  |  |  |  |  |  |  |  |
|  |  |  |  | -81.1047000 | 29.3542000 |  |  |  |  |  |  |  |  |  |  |
|  |  |  |  | -77.6693800 | 2.4203400 |  |  |  |  |  |  |  |  |  |  |
|  |  |  |  | -80.0500000 | 0.0333300 |  |  |  |  |  |  |  |  |  |  |
|  |  |  |  | -66.9156300 | 17.9785000 |  |  |  |  |  |  |  |  |  |  |
|  |  |  |  | -70.7000000 | 18.4200000 |  |  |  |  |  |  |  |  |  |  |
|  |  |  |  | -40.0722000 | -19.3911000 |  |  |  |  |  |  |  |  |  |  |
|  |  |  |  | -46.1386733 | -23.8582696 |  |  |  |  |  |  |  |  |  |  |
|  |  |  |  | -76.4191000 | 8.8661900 |  |  |  |  |  |  |  |  |  |  |
|  |  |  |  | -52.3333300 | 4.9333300 |  |  |  |  |  |  |  |  |  |  |
|  |  |  |  | -80.0833300 | -3.4166600 |  |  |  |  |  |  |  |  |  |  |
|  |  |  |  | -83.7600000 | 9.1800000 |  |  |  |  |  |  |  |  |  |  |
|  |  |  |  | -80.5166600 | -3.5000000 |  |  |  |  |  |  |  |  |  |  |
|  |  |  |  | -105.2500000 | 21.5333300 |  |  |  |  |  |  |  |  |  |  |
|  |  |  |  | -79.8891600 | 9.3277700 |  |  |  |  |  |  |  |  |  |  |
|  |  |  |  | -75.5000000 | 10.4166600 |  |  |  |  |  |  |  |  |  |  |
|  |  |  |  | -69.2800000 | 18.4700000 |  |  |  |  |  |  |  |  |  |  |
|  |  |  |  | -81.3541700 | 24.6694400 |  |  |  |  |  |  |  |  |  |  |
|  |  |  |  | -85.3400000 | 9.8300000 |  |  |  |  |  |  |  |  |  |  |
|  |  |  |  | -71.6500000 | 17.9200000 |  |  |  |  |  |  |  |  |  |  |
|  |  |  |  | -81.6938800 | 7.5044400 |  |  |  |  |  |  |  |  |  |  |
|  |  |  |  | -79.6455500 | 9.0355500 |  |  |  |  |  |  |  |  |  |  |
|  |  |  |  | -75.7000000 | 1.3186400 |  |  |  |  |  |  |  |  |  |  |
|  |  |  |  | -91.5300000 | 18.7700000 |  |  |  |  |  |  |  |  |  |  |
|  |  |  |  | -89.0016600 | 15.6591600 |  |  |  |  |  |  |  |  |  |  |
|  |  |  |  | -85.8376900 | 10.3880600 |  |  |  |  |  |  |  |  |  |  |
|  |  |  |  | -61.6102800 | 12.2189400 |  |  |  |  |  |  |  |  |  |  |
|  |  |  |  | -80.7180000 | 24.8620000 |  |  |  |  |  |  |  |  |  |  |
|  |  |  |  | -80.4440000 | 25.2880000 |  |  |  |  |  |  |  |  |  |  |
|  |  |  |  | -82.0470000 | 26.7430000 |  |  |  |  |  |  |  |  |  |  |
|  |  |  |  | -82.5030000 | 27.7020000 |  |  |  |  |  |  |  |  |  |  |
|  |  |  |  | -80.6050000 | 28.0740000 |  |  |  |  |  |  |  |  |  |  |
|  |  |  |  | -80.7560000 | 28.8220000 |  |  |  |  |  |  |  |  |  |  |
|  |  |  |  | -80.6569000 | 24.9003000 |  |  |  |  |  |  |  |  |  |  |
|  |  |  |  | -61.8896000 | 17.0554800 |  |  |  |  |  |  |  |  |  |  |

**Supplemental Table S3.** The SDM model parameter selection results from ENMEval. For each species, the optimal model as determined by ΔAICc scores is highlighted in gray.

| **Avicennia.germinans** | settings | features | rm | train.AUC | avg.test.AUC | var.test.AUC | avg.diff.AUC | var.diff.AUC | avg.test.orMTP | var.test.orMTP | avg.test.or10pct | var.test.or10pct | AICc | delta.AICc | w.AIC | parameters |
| --- | --- | --- | --- | --- | --- | --- | --- | --- | --- | --- | --- | --- | --- | --- | --- | --- |
| 1 | L_0.5 | L | 0.5 | 0.723 | 0.717 | 0.004 | 0.024 | 0.002 | 0.030 | 0.004 | 0.118 | 0.007 | 4851.207 | 53.900 | 1.61E-12 | 6 |
| 2 | LQ_0.5 | LQ | 0.5 | 0.724 | 0.715 | 0.002 | 0.025 | 0.001 | 0.013 | 0.001 | 0.113 | 0.006 | 4854.605 | 57.298 | 2.95E-13 | 11 |
| 3 | LQH_0.5 | LQH | 0.5 | 0.835 | 0.774 | 0.002 | 0.063 | 0.002 | 0.022 | 0.001 | 0.196 | 0.000 | 4836.258 | 38.951 | 2.84E-09 | 39 |
| 4 | LQHP_0.5 | LQHP | 0.5 | 0.847 | 0.801 | 0.000 | 0.047 | 0.001 | 0.027 | 0.001 | 0.185 | 0.006 | 4806.393 | 9.086 | 0.008691 | 37 |
| 5 | LQHPT_0.5 | LQHPT | 0.5 | 0.881 | 0.821 | 0.001 | 0.058 | 0.001 | 0.030 | 0.004 | 0.237 | 0.010 | 4825.669 | 28.362 | 5.67E-07 | 52 |
| 6 | L_1 | L | 1 | 0.724 | 0.716 | 0.004 | 0.024 | 0.002 | 0.030 | 0.004 | 0.118 | 0.007 | 4852.628 | 55.321 | 7.93E-13 | 6 |
| 7 | LQ_1 | LQ | 1 | 0.725 | 0.716 | 0.003 | 0.025 | 0.001 | 0.013 | 0.001 | 0.104 | 0.005 | 4856.219 | 58.912 | 1.32E-13 | 10 |
| 8 | LQH_1 | LQH | 1 | 0.810 | 0.763 | 0.001 | 0.042 | 0.001 | 0.017 | 0.001 | 0.126 | 0.003 | 4829.458 | 32.151 | 8.52E-08 | 25 |
| 9 | LQHP_1 | LQHP | 1 | 0.827 | 0.792 | 0.000 | 0.031 | 0.001 | 0.013 | 0.000 | 0.139 | 0.003 | 4797.307 | 0.000 | 0.8166688 | 22 |
| 10 | LQHPT_1 | LQHPT | 1 | 0.838 | 0.805 | 0.000 | 0.030 | 0.001 | 0.013 | 0.000 | 0.150 | 0.009 | 4802.625 | 5.318 | 0.0571824 | 30 |
| 11 | L_1.5 | L | 1.5 | 0.724 | 0.716 | 0.004 | 0.025 | 0.002 | 0.022 | 0.002 | 0.113 | 0.007 | 4854.248 | 56.941 | 3.53E-13 | 6 |
| 12 | LQ_1.5 | LQ | 1.5 | 0.725 | 0.717 | 0.003 | 0.026 | 0.001 | 0.013 | 0.001 | 0.100 | 0.005 | 4857.194 | 59.887 | 8.09E-14 | 9 |
| 13 | LQH_1.5 | LQH | 1.5 | 0.787 | 0.751 | 0.001 | 0.029 | 0.001 | 0.022 | 0.002 | 0.127 | 0.003 | 4837.556 | 40.249 | 1.49E-09 | 21 |
| 14 | LQHP_1.5 | LQHP | 1.5 | 0.808 | 0.783 | 0.000 | 0.022 | 0.000 | 0.013 | 0.000 | 0.154 | 0.005 | 4813.670 | 16.363 | 0.0002285 | 20 |
| 15 | LQHPT_1.5 | LQHPT | 1.5 | 0.821 | 0.792 | 0.001 | 0.024 | 0.001 | 0.009 | 0.000 | 0.140 | 0.008 | 4801.195 | 3.888 | 0.1169191 | 20 |
| 16 | L_2 | L | 2 | 0.724 | 0.715 | 0.004 | 0.025 | 0.002 | 0.022 | 0.002 | 0.099 | 0.005 | 4855.967 | 58.660 | 1.49E-13 | 6 |
| 17 | LQ_2 | LQ | 2 | 0.726 | 0.715 | 0.003 | 0.027 | 0.001 | 0.009 | 0.000 | 0.100 | 0.005 | 4860.548 | 63.241 | 1.51E-14 | 9 |
| 18 | LQH_2 | LQH | 2 | 0.764 | 0.737 | 0.001 | 0.026 | 0.001 | 0.026 | 0.003 | 0.126 | 0.005 | 4844.618 | 47.311 | 4.35E-11 | 18 |
| 19 | LQHP_2 | LQHP | 2 | 0.796 | 0.772 | 0.001 | 0.023 | 0.001 | 0.013 | 0.000 | 0.149 | 0.003 | 4819.153 | 21.846 | 1.47E-05 | 16 |
| 20 | LQHPT_2 | LQHPT | 2 | 0.805 | 0.781 | 0.001 | 0.024 | 0.001 | 0.009 | 0.000 | 0.140 | 0.008 | 4813.183 | 15.876 | 0.0002915 | 18 |
| 21 | L_2.5 | L | 2.5 | 0.724 | 0.715 | 0.004 | 0.025 | 0.002 | 0.017 | 0.001 | 0.096 | 0.003 | 4857.790 | 60.482 | 6.00E-14 | 6 |
| 22 | LQ_2.5 | LQ | 2.5 | 0.726 | 0.714 | 0.003 | 0.028 | 0.002 | 0.009 | 0.000 | 0.109 | 0.006 | 4860.181 | 62.874 | 1.82E-14 | 8 |
| 23 | LQH_2.5 | LQH | 2.5 | 0.752 | 0.729 | 0.001 | 0.025 | 0.001 | 0.026 | 0.003 | 0.124 | 0.006 | 4849.264 | 51.957 | 4.26E-12 | 16 |
| 24 | LQHP_2.5 | LQHP | 2.5 | 0.784 | 0.761 | 0.001 | 0.023 | 0.001 | 0.018 | 0.001 | 0.124 | 0.002 | 4829.407 | 32.100 | 8.74E-08 | 15 |
| 25 | LQHPT_2.5 | LQHPT | 2.5 | 0.795 | 0.771 | 0.001 | 0.022 | 0.001 | 0.013 | 0.001 | 0.139 | 0.004 | 4822.838 | 25.531 | 2.33E-06 | 17 |
| 26 | L_3 | L | 3 | 0.724 | 0.714 | 0.004 | 0.025 | 0.002 | 0.017 | 0.001 | 0.096 | 0.003 | 4859.729 | 62.422 | 2.28E-14 | 6 |
| 27 | LQ_3 | LQ | 3 | 0.725 | 0.713 | 0.003 | 0.028 | 0.002 | 0.009 | 0.000 | 0.090 | 0.005 | 4864.370 | 67.063 | 2.24E-15 | 9 |
| 28 | LQH_3 | LQH | 3 | 0.746 | 0.723 | 0.002 | 0.027 | 0.001 | 0.026 | 0.003 | 0.147 | 0.006 | 4849.250 | 51.943 | 4.29E-12 | 13 |
| 29 | LQHP_3 | LQHP | 3 | 0.772 | 0.752 | 0.001 | 0.021 | 0.001 | 0.004 | 0.000 | 0.131 | 0.004 | 4836.420 | 39.113 | 2.62E-09 | 13 |
| 30 | LQHPT_3 | LQHPT | 3 | 0.785 | 0.761 | 0.001 | 0.020 | 0.001 | 0.004 | 0.000 | 0.109 | 0.005 | 4825.044 | 27.737 | 7.75E-07 | 13 |
| 31 | L_3.5 | L | 3.5 | 0.724 | 0.713 | 0.003 | 0.026 | 0.002 | 0.013 | 0.001 | 0.092 | 0.002 | 4861.764 | 64.457 | 8.23E-15 | 6 |
| 32 | LQ_3.5 | LQ | 3.5 | 0.725 | 0.712 | 0.003 | 0.028 | 0.002 | 0.009 | 0.000 | 0.096 | 0.003 | 4866.438 | 69.131 | 7.95E-16 | 9 |
| 33 | LQH_3.5 | LQH | 3.5 | 0.742 | 0.718 | 0.002 | 0.028 | 0.001 | 0.026 | 0.003 | 0.147 | 0.006 | 4850.780 | 53.473 | 2.00E-12 | 11 |
| 34 | LQHP_3.5 | LQHP | 3.5 | 0.761 | 0.742 | 0.001 | 0.022 | 0.001 | 0.004 | 0.000 | 0.138 | 0.004 | 4844.654 | 47.347 | 4.27E-11 | 12 |
| 35 | LQHPT_3.5 | LQHPT | 3.5 | 0.773 | 0.750 | 0.001 | 0.019 | 0.001 | 0.004 | 0.000 | 0.113 | 0.006 | 4833.102 | 35.795 | 1.38E-08 | 12 |
| 36 | L_4 | L | 4 | 0.724 | 0.713 | 0.003 | 0.025 | 0.002 | 0.013 | 0.001 | 0.092 | 0.002 | 4863.890 | 66.583 | 2.84E-15 | 6 |
| 37 | LQ_4 | LQ | 4 | 0.724 | 0.712 | 0.003 | 0.028 | 0.002 | 0.009 | 0.000 | 0.106 | 0.003 | 4866.371 | 69.064 | 8.22E-16 | 8 |
| 38 | LQH_4 | LQH | 4 | 0.740 | 0.716 | 0.002 | 0.027 | 0.002 | 0.017 | 0.001 | 0.133 | 0.006 | 4856.482 | 59.175 | 1.15E-13 | 11 |
| 39 | LQHP_4 | LQHP | 4 | 0.756 | 0.735 | 0.001 | 0.021 | 0.001 | 0.004 | 0.000 | 0.134 | 0.003 | 4851.227 | 53.920 | 1.60E-12 | 12 |
| 40 | LQHPT_4 | LQHPT | 4 | 0.765 | 0.742 | 0.001 | 0.020 | 0.001 | 0.004 | 0.000 | 0.118 | 0.003 | 4838.660 | 41.353 | 8.56E-10 | 11 |
| **Conocarpus.erectus** | settings | features | rm | train.AUC | avg.test.AUC | var.test.AUC | avg.diff.AUC | var.diff.AUC | avg.test.orMTP | var.test.orMTP | avg.test.or10pct | var.test.or10pct | AICc | delta.AICc | w.AIC | parameters |
| 1 | L_0.5 | L | 0.5 | 0.759 | 0.742 | 0.007 | 0.042 | 0.005 | 0.005 | 0.000 | 0.219 | 0.028 | 3838.648 | 44.236 | 2.00E-10 | 6 |
| 2 | LQ_0.5 | LQ | 0.5 | 0.792 | 0.755 | 0.004 | 0.049 | 0.006 | 0.010 | 0.000 | 0.194 | 0.018 | 3811.222 | 16.810 | 0.0001808 | 10 |
| 3 | LQH_0.5 | LQH | 0.5 | 0.876 | 0.823 | 0.004 | 0.057 | 0.005 | 0.005 | 0.000 | 0.243 | 0.012 | 3813.960 | 19.548 | 4.60E-05 | 33 |
| 4 | LQHP_0.5 | LQHP | 0.5 | 0.884 | 0.830 | 0.004 | 0.060 | 0.007 | 0.005 | 0.000 | 0.252 | 0.013 | 3816.534 | 22.121 | 1.27E-05 | 38 |
| 5 | LQHPT_0.5 | LQHPT | 0.5 | 0.918 | 0.850 | 0.006 | 0.068 | 0.007 | 0.010 | 0.000 | 0.249 | 0.022 | 3819.712 | 25.299 | 2.59E-06 | 49 |
| 6 | L_1 | L | 1 | 0.756 | 0.740 | 0.007 | 0.040 | 0.005 | 0.005 | 0.000 | 0.204 | 0.024 | 3840.239 | 45.827 | 9.04E-11 | 6 |
| 7 | LQ_1 | LQ | 1 | 0.785 | 0.755 | 0.006 | 0.045 | 0.006 | 0.005 | 0.000 | 0.170 | 0.025 | 3819.826 | 25.414 | 2.45E-06 | 10 |
| 8 | LQH_1 | LQH | 1 | 0.855 | 0.809 | 0.004 | 0.049 | 0.005 | 0.005 | 0.000 | 0.188 | 0.010 | 3803.175 | 8.763 | 0.0101102 | 20 |
| 9 | LQHP_1 | LQHP | 1 | 0.858 | 0.812 | 0.005 | 0.056 | 0.006 | 0.005 | 0.000 | 0.223 | 0.021 | 3799.664 | 5.252 | 0.0584821 | 22 |
| 10 | LQHPT_1 | LQHPT | 1 | 0.871 | 0.819 | 0.005 | 0.058 | 0.006 | 0.005 | 0.000 | 0.190 | 0.014 | 3798.474 | 4.061 | 0.1060729 | 25 |
| 11 | L_1.5 | L | 1.5 | 0.753 | 0.738 | 0.006 | 0.039 | 0.004 | 0.005 | 0.000 | 0.170 | 0.025 | 3842.145 | 47.733 | 3.49E-11 | 6 |
| 12 | LQ_1.5 | LQ | 1.5 | 0.771 | 0.753 | 0.007 | 0.043 | 0.006 | 0.005 | 0.000 | 0.147 | 0.025 | 3829.467 | 35.054 | 1.97E-08 | 10 |
| 13 | LQH_1.5 | LQH | 1.5 | 0.843 | 0.799 | 0.004 | 0.049 | 0.005 | 0.005 | 0.000 | 0.165 | 0.007 | 3805.443 | 11.030 | 0.0032533 | 16 |
| 14 | LQHP_1.5 | LQHP | 1.5 | 0.848 | 0.807 | 0.006 | 0.053 | 0.006 | 0.005 | 0.000 | 0.167 | 0.005 | 3806.817 | 12.405 | 0.0016363 | 20 |
| 15 | LQHPT_1.5 | LQHPT | 1.5 | 0.851 | 0.811 | 0.006 | 0.054 | 0.005 | 0.005 | 0.000 | 0.215 | 0.017 | 3794.412 | 0.000 | 0.8082237 | 17 |
| 16 | L_2 | L | 2 | 0.748 | 0.737 | 0.006 | 0.037 | 0.004 | 0.005 | 0.000 | 0.175 | 0.027 | 3844.315 | 49.903 | 1.18E-11 | 6 |
| 17 | LQ_2 | LQ | 2 | 0.763 | 0.749 | 0.008 | 0.042 | 0.005 | 0.005 | 0.000 | 0.167 | 0.020 | 3833.409 | 38.997 | 2.75E-09 | 8 |
| 18 | LQH_2 | LQH | 2 | 0.830 | 0.784 | 0.005 | 0.052 | 0.004 | 0.005 | 0.000 | 0.194 | 0.009 | 3809.452 | 15.039 | 0.0004383 | 14 |
| 19 | LQHP_2 | LQHP | 2 | 0.840 | 0.797 | 0.006 | 0.054 | 0.006 | 0.005 | 0.000 | 0.225 | 0.011 | 3806.570 | 12.158 | 0.0018515 | 16 |
| 20 | LQHPT_2 | LQHPT | 2 | 0.844 | 0.803 | 0.006 | 0.053 | 0.006 | 0.005 | 0.000 | 0.213 | 0.016 | 3803.854 | 9.442 | 0.0071976 | 17 |
| 21 | L_2.5 | L | 2.5 | 0.746 | 0.735 | 0.006 | 0.035 | 0.004 | 0.005 | 0.000 | 0.157 | 0.029 | 3843.605 | 49.192 | 1.68E-11 | 5 |
| 22 | LQ_2.5 | LQ | 2.5 | 0.757 | 0.744 | 0.008 | 0.040 | 0.005 | 0.005 | 0.000 | 0.167 | 0.020 | 3838.108 | 43.696 | 2.62E-10 | 7 |
| 23 | LQH_2.5 | LQH | 2.5 | 0.822 | 0.772 | 0.007 | 0.053 | 0.005 | 0.005 | 0.000 | 0.188 | 0.010 | 3812.568 | 18.156 | 9.23E-05 | 12 |
| 24 | LQHP_2.5 | LQHP | 2.5 | 0.831 | 0.790 | 0.007 | 0.054 | 0.007 | 0.005 | 0.000 | 0.223 | 0.014 | 3808.597 | 14.185 | 0.0006719 | 13 |
| 25 | LQHPT_2.5 | LQHPT | 2.5 | 0.836 | 0.794 | 0.007 | 0.054 | 0.006 | 0.005 | 0.000 | 0.203 | 0.013 | 3806.818 | 12.406 | 0.0016355 | 14 |
| 26 | L_3 | L | 3 | 0.744 | 0.733 | 0.006 | 0.034 | 0.003 | 0.005 | 0.000 | 0.147 | 0.025 | 3844.902 | 50.490 | 8.79E-12 | 5 |
| 27 | LQ_3 | LQ | 3 | 0.751 | 0.741 | 0.007 | 0.039 | 0.005 | 0.005 | 0.000 | 0.172 | 0.022 | 3844.129 | 49.717 | 1.29E-11 | 7 |
| 28 | LQH_3 | LQH | 3 | 0.812 | 0.764 | 0.008 | 0.049 | 0.005 | 0.005 | 0.000 | 0.168 | 0.016 | 3815.971 | 21.559 | 1.68E-05 | 10 |
| 29 | LQHP_3 | LQHP | 3 | 0.825 | 0.783 | 0.008 | 0.053 | 0.007 | 0.005 | 0.000 | 0.208 | 0.018 | 3814.270 | 19.858 | 3.94E-05 | 12 |
| 30 | LQHPT_3 | LQHPT | 3 | 0.830 | 0.787 | 0.008 | 0.052 | 0.007 | 0.005 | 0.000 | 0.218 | 0.021 | 3815.262 | 20.850 | 2.40E-05 | 14 |
| 31 | L_3.5 | L | 3.5 | 0.742 | 0.731 | 0.006 | 0.033 | 0.003 | 0.005 | 0.000 | 0.142 | 0.023 | 3843.949 | 49.537 | 1.42E-11 | 4 |
| 32 | LQ_3.5 | LQ | 3.5 | 0.749 | 0.736 | 0.006 | 0.038 | 0.004 | 0.005 | 0.000 | 0.167 | 0.020 | 3843.261 | 48.849 | 2.00E-11 | 6 |
| 33 | LQH_3.5 | LQH | 3.5 | 0.802 | 0.755 | 0.008 | 0.044 | 0.004 | 0.005 | 0.000 | 0.168 | 0.016 | 3820.617 | 26.205 | 1.65E-06 | 9 |
| 34 | LQHP_3.5 | LQHP | 3.5 | 0.816 | 0.773 | 0.008 | 0.053 | 0.007 | 0.005 | 0.000 | 0.217 | 0.023 | 3817.790 | 23.378 | 6.78E-06 | 10 |
| 35 | LQHPT_3.5 | LQHPT | 3.5 | 0.822 | 0.776 | 0.008 | 0.053 | 0.007 | 0.005 | 0.000 | 0.217 | 0.023 | 3821.882 | 27.470 | 8.76E-07 | 13 |
| 36 | L_4 | L | 4 | 0.741 | 0.730 | 0.006 | 0.033 | 0.003 | 0.005 | 0.000 | 0.155 | 0.019 | 3844.810 | 50.397 | 9.20E-12 | 4 |
| 37 | LQ_4 | LQ | 4 | 0.748 | 0.734 | 0.006 | 0.038 | 0.004 | 0.005 | 0.000 | 0.157 | 0.018 | 3844.653 | 50.241 | 9.95E-12 | 6 |
| 38 | LQH_4 | LQH | 4 | 0.787 | 0.751 | 0.008 | 0.040 | 0.005 | 0.010 | 0.000 | 0.173 | 0.018 | 3828.212 | 33.800 | 3.70E-08 | 9 |
| 39 | LQHP_4 | LQHP | 4 | 0.805 | 0.764 | 0.008 | 0.050 | 0.007 | 0.005 | 0.000 | 0.210 | 0.024 | 3828.247 | 33.835 | 3.63E-08 | 11 |
| 40 | LQHPT_4 | LQHPT | 4 | 0.811 | 0.767 | 0.008 | 0.050 | 0.007 | 0.005 | 0.000 | 0.205 | 0.022 | 3824.752 | 30.340 | 2.09E-07 | 11 |
| **Laguncularia.racemosa** | settings | features | rm | train.AUC | avg.test.AUC | var.test.AUC | avg.diff.AUC | var.diff.AUC | avg.test.orMTP | var.test.orMTP | avg.test.or10pct | var.test.or10pct | AICc | delta.AICc | w.AIC | parameters |
| 1 | L_0.5 | L | 0.5 | 0.774 | 0.769 | 0.001 | 0.018 | 0.000 | 0.004 | 0.000 | 0.104 | 0.003 | 5319.191 | 95.090 | 2.25E-21 | 5 |
| 2 | LQ_0.5 | LQ | 0.5 | 0.803 | 0.789 | 0.002 | 0.021 | 0.001 | 0.004 | 0.000 | 0.125 | 0.002 | 5276.663 | 52.561 | 3.86E-12 | 11 |
| 3 | LQH_0.5 | LQH | 0.5 | 0.868 | 0.826 | 0.001 | 0.044 | 0.002 | 0.011 | 0.000 | 0.178 | 0.011 | 5262.219 | 38.118 | 5.28E-09 | 39 |
| 4 | LQHP_0.5 | LQHP | 0.5 | 0.880 | 0.845 | 0.001 | 0.038 | 0.001 | 0.021 | 0.000 | 0.154 | 0.005 | 5224.101 | 0.000 | 0.9994985 | 38 |
| 5 | LQHPT_0.5 | LQHPT | 0.5 | 0.907 | 0.854 | 0.001 | 0.055 | 0.001 | 0.041 | 0.000 | 0.259 | 0.002 | 5251.688 | 27.587 | 1.02E-06 | 56 |
| 6 | L_1 | L | 1 | 0.774 | 0.768 | 0.001 | 0.019 | 0.000 | 0.004 | 0.000 | 0.104 | 0.003 | 5320.534 | 96.433 | 1.15E-21 | 5 |
| 7 | LQ_1 | LQ | 1 | 0.799 | 0.786 | 0.002 | 0.023 | 0.001 | 0.004 | 0.000 | 0.149 | 0.009 | 5284.199 | 60.098 | 8.91E-14 | 9 |
| 8 | LQH_1 | LQH | 1 | 0.844 | 0.813 | 0.001 | 0.029 | 0.001 | 0.008 | 0.000 | 0.148 | 0.002 | 5266.721 | 42.620 | 5.56E-10 | 28 |
| 9 | LQHP_1 | LQHP | 1 | 0.861 | 0.835 | 0.000 | 0.024 | 0.000 | 0.011 | 0.000 | 0.118 | 0.001 | 5239.308 | 15.207 | 0.0004984 | 30 |
| 10 | LQHPT_1 | LQHPT | 1 | 0.869 | 0.839 | 0.000 | 0.034 | 0.000 | 0.011 | 0.000 | 0.134 | 0.001 | 5251.702 | 27.601 | 1.01E-06 | 38 |
| 11 | L_1.5 | L | 1.5 | 0.774 | 0.768 | 0.001 | 0.019 | 0.000 | 0.004 | 0.000 | 0.104 | 0.003 | 5321.898 | 97.797 | 5.80E-22 | 5 |
| 12 | LQ_1.5 | LQ | 1.5 | 0.795 | 0.784 | 0.002 | 0.023 | 0.001 | 0.004 | 0.000 | 0.136 | 0.009 | 5291.529 | 67.428 | 2.28E-15 | 7 |
| 13 | LQH_1.5 | LQH | 1.5 | 0.826 | 0.805 | 0.001 | 0.023 | 0.001 | 0.004 | 0.000 | 0.125 | 0.003 | 5264.320 | 40.219 | 1.85E-09 | 18 |
| 14 | LQHP_1.5 | LQHP | 1.5 | 0.842 | 0.825 | 0.000 | 0.019 | 0.000 | 0.007 | 0.000 | 0.110 | 0.002 | 5251.726 | 27.625 | 1.00E-06 | 23 |
| 15 | LQHPT_1.5 | LQHPT | 1.5 | 0.845 | 0.826 | 0.001 | 0.021 | 0.000 | 0.007 | 0.000 | 0.123 | 0.001 | 5262.505 | 38.404 | 4.58E-09 | 28 |
| 16 | L_2 | L | 2 | 0.774 | 0.768 | 0.001 | 0.019 | 0.000 | 0.004 | 0.000 | 0.104 | 0.003 | 5323.227 | 99.126 | 2.98E-22 | 5 |
| 17 | LQ_2 | LQ | 2 | 0.793 | 0.782 | 0.001 | 0.023 | 0.001 | 0.004 | 0.000 | 0.118 | 0.004 | 5299.456 | 75.355 | 4.33E-17 | 7 |
| 18 | LQH_2 | LQH | 2 | 0.820 | 0.799 | 0.001 | 0.020 | 0.001 | 0.004 | 0.000 | 0.114 | 0.001 | 5266.151 | 42.050 | 7.39E-10 | 14 |
| 19 | LQHP_2 | LQHP | 2 | 0.832 | 0.814 | 0.001 | 0.018 | 0.001 | 0.007 | 0.000 | 0.109 | 0.001 | 5262.272 | 38.171 | 5.14E-09 | 19 |
| 20 | LQHPT_2 | LQHPT | 2 | 0.832 | 0.813 | 0.001 | 0.020 | 0.001 | 0.007 | 0.000 | 0.109 | 0.001 | 5264.429 | 40.328 | 1.75E-09 | 20 |
| 21 | L_2.5 | L | 2.5 | 0.774 | 0.767 | 0.001 | 0.020 | 0.001 | 0.004 | 0.000 | 0.104 | 0.003 | 5324.681 | 100.580 | 1.44E-22 | 5 |
| 22 | LQ_2.5 | LQ | 2.5 | 0.788 | 0.779 | 0.001 | 0.023 | 0.001 | 0.004 | 0.000 | 0.114 | 0.003 | 5307.653 | 83.552 | 7.19E-19 | 7 |
| 23 | LQH_2.5 | LQH | 2.5 | 0.813 | 0.794 | 0.001 | 0.021 | 0.001 | 0.004 | 0.000 | 0.116 | 0.002 | 5278.042 | 53.941 | 1.93E-12 | 15 |
| 24 | LQHP_2.5 | LQHP | 2.5 | 0.820 | 0.806 | 0.001 | 0.018 | 0.001 | 0.007 | 0.000 | 0.121 | 0.002 | 5274.017 | 49.916 | 1.45E-11 | 17 |
| 25 | LQHPT_2.5 | LQHPT | 2.5 | 0.820 | 0.804 | 0.001 | 0.019 | 0.001 | 0.007 | 0.000 | 0.121 | 0.002 | 5276.107 | 52.006 | 5.09E-12 | 18 |
| 26 | L_3 | L | 3 | 0.774 | 0.766 | 0.002 | 0.020 | 0.001 | 0.004 | 0.000 | 0.104 | 0.003 | 5326.186 | 102.085 | 6.80E-23 | 5 |
| 27 | LQ_3 | LQ | 3 | 0.784 | 0.775 | 0.001 | 0.022 | 0.001 | 0.004 | 0.000 | 0.110 | 0.003 | 5315.958 | 91.857 | 1.13E-20 | 7 |
| 28 | LQH_3 | LQH | 3 | 0.809 | 0.791 | 0.001 | 0.021 | 0.001 | 0.004 | 0.000 | 0.116 | 0.002 | 5284.264 | 60.163 | 8.62E-14 | 14 |
| 29 | LQHP_3 | LQHP | 3 | 0.811 | 0.798 | 0.001 | 0.018 | 0.001 | 0.004 | 0.000 | 0.118 | 0.002 | 5284.150 | 60.049 | 9.13E-14 | 15 |
| 30 | LQHPT_3 | LQHPT | 3 | 0.811 | 0.797 | 0.002 | 0.019 | 0.001 | 0.016 | 0.000 | 0.105 | 0.001 | 5285.363 | 61.262 | 4.98E-14 | 16 |
| 31 | L_3.5 | L | 3.5 | 0.774 | 0.765 | 0.002 | 0.021 | 0.001 | 0.004 | 0.000 | 0.104 | 0.003 | 5327.730 | 103.629 | 3.14E-23 | 5 |
| 32 | LQ_3.5 | LQ | 3.5 | 0.780 | 0.772 | 0.001 | 0.022 | 0.001 | 0.004 | 0.000 | 0.110 | 0.003 | 5324.210 | 100.109 | 1.83E-22 | 7 |
| 33 | LQH_3.5 | LQH | 3.5 | 0.806 | 0.788 | 0.001 | 0.021 | 0.001 | 0.004 | 0.000 | 0.116 | 0.002 | 5289.752 | 65.651 | 5.54E-15 | 13 |
| 34 | LQHP_3.5 | LQHP | 3.5 | 0.808 | 0.791 | 0.001 | 0.018 | 0.001 | 0.004 | 0.000 | 0.118 | 0.002 | 5288.063 | 63.962 | 1.29E-14 | 13 |
| 35 | LQHPT_3.5 | LQHPT | 3.5 | 0.809 | 0.790 | 0.001 | 0.020 | 0.001 | 0.013 | 0.000 | 0.105 | 0.001 | 5288.694 | 64.592 | 9.41E-15 | 14 |
| 36 | L_4 | L | 4 | 0.774 | 0.765 | 0.002 | 0.021 | 0.001 | 0.004 | 0.000 | 0.104 | 0.003 | 5329.329 | 105.228 | 1.41E-23 | 5 |
| 37 | LQ_4 | LQ | 4 | 0.779 | 0.770 | 0.001 | 0.021 | 0.001 | 0.004 | 0.000 | 0.110 | 0.003 | 5324.201 | 100.100 | 1.83E-22 | 6 |
| 38 | LQH_4 | LQH | 4 | 0.802 | 0.784 | 0.001 | 0.022 | 0.001 | 0.004 | 0.000 | 0.120 | 0.003 | 5294.934 | 70.833 | 4.15E-16 | 12 |
| 39 | LQHP_4 | LQHP | 4 | 0.806 | 0.788 | 0.001 | 0.018 | 0.001 | 0.004 | 0.000 | 0.118 | 0.002 | 5290.625 | 66.524 | 3.58E-15 | 11 |
| 40 | LQHPT_4 | LQHPT | 4 | 0.807 | 0.787 | 0.001 | 0.020 | 0.001 | 0.004 | 0.000 | 0.127 | 0.002 | 5288.600 | 64.499 | 9.87E-15 | 11 |
| **Rhizophora.mangle** | settings | features | rm | train.AUC | avg.test.AUC | var.test.AUC | avg.diff.AUC | var.diff.AUC | avg.test.orMTP | var.test.orMTP | avg.test.or10pct | var.test.or10pct | AICc | delta.AICc | w.AIC | parameters |
| 1 | L_0.5 | L | 0.5 | 0.766 | 0.758 | 0.002 | 0.023 | 0.001 | 0.004 | 0.000 | 0.096 | 0.002 | 4859.459 | 84.326 | 2.58E-19 | 5 |
| 2 | LQ_0.5 | LQ | 0.5 | 0.806 | 0.789 | 0.003 | 0.031 | 0.001 | 0.004 | 0.000 | 0.127 | 0.007 | 4807.433 | 32.299 | 5.12E-08 | 10 |
| 3 | LQH_0.5 | LQH | 0.5 | 0.866 | 0.817 | 0.003 | 0.051 | 0.004 | 0.008 | 0.000 | 0.242 | 0.011 | 4806.697 | 31.563 | 7.39E-08 | 39 |
| 4 | LQHP_0.5 | LQHP | 0.5 | 0.873 | 0.834 | 0.001 | 0.043 | 0.002 | 0.020 | 0.000 | 0.184 | 0.005 | 4775.134 | 0.000 | 0.5278998 | 40 |
| 5 | LQHPT_0.5 | LQHPT | 0.5 | 0.904 | 0.852 | 0.002 | 0.054 | 0.003 | 0.032 | 0.002 | 0.252 | 0.021 | 4818.258 | 43.124 | 2.28E-10 | 62 |
| 6 | L_1 | L | 1 | 0.766 | 0.757 | 0.002 | 0.024 | 0.001 | 0.004 | 0.000 | 0.104 | 0.004 | 4860.436 | 85.302 | 1.58E-19 | 5 |
| 7 | LQ_1 | LQ | 1 | 0.801 | 0.786 | 0.002 | 0.031 | 0.001 | 0.004 | 0.000 | 0.124 | 0.005 | 4817.199 | 42.065 | 3.87E-10 | 9 |
| 8 | LQH_1 | LQH | 1 | 0.846 | 0.811 | 0.002 | 0.039 | 0.002 | 0.008 | 0.000 | 0.182 | 0.006 | 4814.425 | 39.292 | 1.55E-09 | 28 |
| 9 | LQHP_1 | LQHP | 1 | 0.854 | 0.829 | 0.001 | 0.030 | 0.001 | 0.012 | 0.000 | 0.153 | 0.002 | 4783.249 | 8.115 | 0.0091296 | 28 |
| 10 | LQHPT_1 | LQHPT | 1 | 0.864 | 0.827 | 0.001 | 0.035 | 0.002 | 0.012 | 0.000 | 0.150 | 0.002 | 4791.404 | 16.271 | 0.0001547 | 36 |
| 11 | L_1.5 | L | 1.5 | 0.766 | 0.756 | 0.002 | 0.024 | 0.001 | 0.004 | 0.000 | 0.104 | 0.004 | 4861.429 | 86.295 | 9.64E-20 | 5 |
| 12 | LQ_1.5 | LQ | 1.5 | 0.792 | 0.782 | 0.002 | 0.030 | 0.001 | 0.004 | 0.000 | 0.124 | 0.005 | 4829.305 | 54.172 | 9.11E-13 | 9 |
| 13 | LQH_1.5 | LQH | 1.5 | 0.835 | 0.811 | 0.002 | 0.029 | 0.001 | 0.004 | 0.000 | 0.147 | 0.003 | 4808.533 | 33.399 | 2.95E-08 | 18 |
| 14 | LQHP_1.5 | LQHP | 1.5 | 0.843 | 0.825 | 0.001 | 0.022 | 0.001 | 0.008 | 0.000 | 0.134 | 0.001 | 4777.642 | 2.509 | 0.1505995 | 16 |
| 15 | LQHPT_1.5 | LQHPT | 1.5 | 0.844 | 0.823 | 0.001 | 0.026 | 0.001 | 0.012 | 0.000 | 0.142 | 0.002 | 4776.185 | 1.051 | 0.3121814 | 17 |
| 16 | L_2 | L | 2 | 0.766 | 0.756 | 0.002 | 0.025 | 0.001 | 0.004 | 0.000 | 0.104 | 0.004 | 4862.396 | 87.262 | 5.94E-20 | 5 |
| 17 | LQ_2 | LQ | 2 | 0.785 | 0.777 | 0.002 | 0.027 | 0.001 | 0.004 | 0.000 | 0.114 | 0.003 | 4839.286 | 64.152 | 6.20E-15 | 8 |
| 18 | LQH_2 | LQH | 2 | 0.827 | 0.804 | 0.002 | 0.027 | 0.001 | 0.004 | 0.000 | 0.124 | 0.003 | 4813.908 | 38.774 | 2.01E-09 | 14 |
| 19 | LQHP_2 | LQHP | 2 | 0.837 | 0.819 | 0.001 | 0.019 | 0.001 | 0.008 | 0.000 | 0.139 | 0.002 | 4802.077 | 26.943 | 7.45E-07 | 20 |
| 20 | LQHPT_2 | LQHPT | 2 | 0.838 | 0.819 | 0.001 | 0.023 | 0.001 | 0.012 | 0.000 | 0.150 | 0.002 | 4794.432 | 19.298 | 3.40E-05 | 18 |
| 21 | L_2.5 | L | 2.5 | 0.766 | 0.756 | 0.002 | 0.024 | 0.001 | 0.004 | 0.000 | 0.104 | 0.004 | 4863.477 | 88.343 | 3.46E-20 | 5 |
| 22 | LQ_2.5 | LQ | 2.5 | 0.778 | 0.771 | 0.002 | 0.026 | 0.001 | 0.004 | 0.000 | 0.095 | 0.004 | 4847.007 | 71.873 | 1.30E-16 | 6 |
| 23 | LQH_2.5 | LQH | 2.5 | 0.818 | 0.796 | 0.002 | 0.026 | 0.001 | 0.004 | 0.000 | 0.132 | 0.004 | 4830.561 | 55.427 | 4.86E-13 | 16 |
| 24 | LQHP_2.5 | LQHP | 2.5 | 0.829 | 0.814 | 0.001 | 0.020 | 0.001 | 0.008 | 0.000 | 0.139 | 0.001 | 4810.271 | 35.138 | 1.24E-08 | 17 |
| 25 | LQHPT_2.5 | LQHPT | 2.5 | 0.831 | 0.815 | 0.001 | 0.022 | 0.001 | 0.012 | 0.000 | 0.154 | 0.002 | 4810.100 | 34.966 | 1.35E-08 | 18 |
| 26 | L_3 | L | 3 | 0.766 | 0.756 | 0.002 | 0.024 | 0.001 | 0.004 | 0.000 | 0.104 | 0.004 | 4864.597 | 89.463 | 1.98E-20 | 5 |
| 27 | LQ_3 | LQ | 3 | 0.774 | 0.765 | 0.001 | 0.025 | 0.001 | 0.004 | 0.000 | 0.107 | 0.003 | 4855.042 | 79.908 | 2.35E-18 | 6 |
| 28 | LQH_3 | LQH | 3 | 0.805 | 0.790 | 0.001 | 0.023 | 0.001 | 0.004 | 0.000 | 0.128 | 0.004 | 4836.440 | 61.306 | 2.57E-14 | 14 |
| 29 | LQHP_3 | LQHP | 3 | 0.821 | 0.809 | 0.001 | 0.020 | 0.001 | 0.008 | 0.000 | 0.122 | 0.001 | 4814.835 | 39.702 | 1.26E-09 | 13 |
| 30 | LQHPT_3 | LQHPT | 3 | 0.823 | 0.809 | 0.001 | 0.022 | 0.001 | 0.012 | 0.000 | 0.136 | 0.001 | 4810.215 | 35.081 | 1.27E-08 | 12 |
| 31 | L_3.5 | L | 3.5 | 0.766 | 0.755 | 0.002 | 0.024 | 0.001 | 0.004 | 0.000 | 0.108 | 0.004 | 4865.767 | 90.634 | 1.10E-20 | 5 |
| 32 | LQ_3.5 | LQ | 3.5 | 0.769 | 0.761 | 0.001 | 0.023 | 0.001 | 0.000 | 0.000 | 0.103 | 0.003 | 4862.754 | 87.620 | 4.97E-20 | 6 |
| 33 | LQH_3.5 | LQH | 3.5 | 0.797 | 0.783 | 0.001 | 0.023 | 0.001 | 0.004 | 0.000 | 0.113 | 0.003 | 4838.733 | 63.599 | 8.17E-15 | 12 |
| 34 | LQHP_3.5 | LQHP | 3.5 | 0.812 | 0.804 | 0.001 | 0.021 | 0.001 | 0.008 | 0.000 | 0.122 | 0.001 | 4824.730 | 49.596 | 8.97E-12 | 12 |
| 35 | LQHPT_3.5 | LQHPT | 3.5 | 0.813 | 0.804 | 0.001 | 0.022 | 0.001 | 0.008 | 0.000 | 0.122 | 0.001 | 4827.695 | 52.561 | 2.04E-12 | 14 |
| 36 | L_4 | L | 4 | 0.765 | 0.755 | 0.001 | 0.023 | 0.001 | 0.004 | 0.000 | 0.108 | 0.004 | 4866.982 | 91.848 | 6.00E-21 | 5 |
| 37 | LQ_4 | LQ | 4 | 0.768 | 0.760 | 0.001 | 0.022 | 0.001 | 0.000 | 0.000 | 0.107 | 0.002 | 4862.013 | 86.879 | 7.19E-20 | 5 |
| 38 | LQH_4 | LQH | 4 | 0.795 | 0.778 | 0.001 | 0.023 | 0.001 | 0.004 | 0.000 | 0.109 | 0.003 | 4833.624 | 58.490 | 1.05E-13 | 8 |
| 39 | LQHP_4 | LQHP | 4 | 0.805 | 0.797 | 0.001 | 0.022 | 0.001 | 0.008 | 0.000 | 0.125 | 0.001 | 4830.145 | 55.011 | 5.99E-13 | 10 |
| 40 | LQHPT_4 | LQHPT | 4 | 0.806 | 0.799 | 0.001 | 0.023 | 0.001 | 0.008 | 0.000 | 0.125 | 0.001 | 4831.788 | 56.654 | 2.63E-13 | 11 |
| **Batis.maritima** | settings | features | rm | train.AUC | avg.test.AUC | var.test.AUC | avg.diff.AUC | var.diff.AUC | avg.test.orMTP | var.test.orMTP | avg.test.or10pct | var.test.or10pct | AICc | delta.AICc | w.AIC | parameters |
| 1 | L_0.5 | L | 0.5 | 0.734 | 0.719 | 0.016 | 0.044 | 0.017 | 0.007 | 0.000 | 0.147 | 0.032 | 2934.294 | 53.007 | 1.85E-12 | 6 |
| 2 | LQ_0.5 | LQ | 0.5 | 0.750 | 0.729 | 0.011 | 0.045 | 0.012 | 0.000 | 0.000 | 0.153 | 0.028 | 2928.855 | 47.567 | 2.81E-11 | 10 |
| 3 | LQH_0.5 | LQH | 0.5 | 0.893 | 0.808 | 0.008 | 0.081 | 0.009 | 0.013 | 0.000 | 0.244 | 0.022 | 2972.663 | 91.375 | 8.62E-21 | 46 |
| 4 | LQHP_0.5 | LQHP | 0.5 | 0.907 | 0.840 | 0.001 | 0.066 | 0.001 | 0.014 | 0.001 | 0.260 | 0.002 | 2989.609 | 108.322 | 1.80E-24 | 49 |
| 5 | LQHPT_0.5 | LQHPT | 0.5 | 0.941 | 0.864 | 0.001 | 0.075 | 0.002 | 0.101 | 0.003 | 0.282 | 0.013 | 3024.405 | 143.118 | 5.01E-32 | 65 |
| 6 | L_1 | L | 1 | 0.733 | 0.720 | 0.017 | 0.045 | 0.018 | 0.007 | 0.000 | 0.147 | 0.032 | 2935.112 | 53.824 | 1.23E-12 | 6 |
| 7 | LQ_1 | LQ | 1 | 0.747 | 0.729 | 0.014 | 0.047 | 0.013 | 0.000 | 0.000 | 0.147 | 0.032 | 2929.494 | 48.207 | 2.04E-11 | 9 |
| 8 | LQH_1 | LQH | 1 | 0.847 | 0.793 | 0.011 | 0.077 | 0.007 | 0.013 | 0.000 | 0.175 | 0.015 | 2882.257 | 0.969 | 0.3687984 | 19 |
| 9 | LQHP_1 | LQHP | 1 | 0.870 | 0.807 | 0.003 | 0.063 | 0.003 | 0.000 | 0.000 | 0.209 | 0.022 | 2893.314 | 12.027 | 0.0014645 | 24 |
| 10 | LQHPT_1 | LQHPT | 1 | 0.889 | 0.811 | 0.002 | 0.073 | 0.003 | 0.000 | 0.000 | 0.245 | 0.016 | 2904.930 | 23.642 | 4.40E-06 | 32 |
| 11 | L_1.5 | L | 1.5 | 0.732 | 0.720 | 0.017 | 0.045 | 0.018 | 0.007 | 0.000 | 0.147 | 0.032 | 2936.069 | 54.781 | 7.61E-13 | 6 |
| 12 | LQ_1.5 | LQ | 1.5 | 0.743 | 0.730 | 0.016 | 0.046 | 0.015 | 0.007 | 0.000 | 0.147 | 0.032 | 2933.507 | 52.219 | 2.74E-12 | 9 |
| 13 | LQH_1.5 | LQH | 1.5 | 0.825 | 0.784 | 0.012 | 0.066 | 0.008 | 0.025 | 0.001 | 0.160 | 0.035 | 2881.288 | 0.000 | 0.5987044 | 14 |
| 14 | LQHP_1.5 | LQHP | 1.5 | 0.845 | 0.783 | 0.005 | 0.064 | 0.006 | 0.000 | 0.000 | 0.215 | 0.021 | 2889.151 | 7.864 | 0.0117393 | 19 |
| 15 | LQHPT_1.5 | LQHPT | 1.5 | 0.848 | 0.783 | 0.004 | 0.066 | 0.005 | 0.000 | 0.000 | 0.227 | 0.020 | 2899.277 | 17.990 | 7.43E-05 | 24 |
| 16 | L_2 | L | 2 | 0.731 | 0.720 | 0.018 | 0.045 | 0.018 | 0.007 | 0.000 | 0.147 | 0.032 | 2937.069 | 55.782 | 4.62E-13 | 6 |
| 17 | LQ_2 | LQ | 2 | 0.741 | 0.729 | 0.016 | 0.047 | 0.016 | 0.007 | 0.000 | 0.147 | 0.032 | 2933.702 | 52.415 | 2.49E-12 | 8 |
| 18 | LQH_2 | LQH | 2 | 0.809 | 0.778 | 0.015 | 0.057 | 0.010 | 0.019 | 0.001 | 0.160 | 0.035 | 2888.257 | 6.969 | 0.0183573 | 12 |
| 19 | LQHP_2 | LQHP | 2 | 0.824 | 0.774 | 0.009 | 0.059 | 0.010 | 0.000 | 0.000 | 0.217 | 0.026 | 2898.335 | 17.047 | 0.000119 | 17 |
| 20 | LQHPT_2 | LQHPT | 2 | 0.826 | 0.775 | 0.007 | 0.061 | 0.010 | 0.000 | 0.000 | 0.215 | 0.021 | 2895.619 | 14.332 | 0.0004625 | 17 |
| 21 | L_2.5 | L | 2.5 | 0.731 | 0.719 | 0.019 | 0.047 | 0.019 | 0.007 | 0.000 | 0.154 | 0.037 | 2938.144 | 56.857 | 2.70E-13 | 6 |
| 22 | LQ_2.5 | LQ | 2.5 | 0.740 | 0.728 | 0.017 | 0.046 | 0.017 | 0.007 | 0.000 | 0.147 | 0.032 | 2936.072 | 54.785 | 7.60E-13 | 8 |
| 23 | LQH_2.5 | LQH | 2.5 | 0.797 | 0.769 | 0.016 | 0.049 | 0.013 | 0.019 | 0.001 | 0.147 | 0.032 | 2896.797 | 15.510 | 0.0002567 | 11 |
| 24 | LQHP_2.5 | LQHP | 2.5 | 0.812 | 0.768 | 0.012 | 0.056 | 0.014 | 0.018 | 0.001 | 0.217 | 0.026 | 2904.274 | 22.986 | 6.11E-06 | 15 |
| 25 | LQHPT_2.5 | LQHPT | 2.5 | 0.814 | 0.770 | 0.010 | 0.057 | 0.013 | 0.018 | 0.001 | 0.210 | 0.022 | 2907.597 | 26.310 | 1.16E-06 | 17 |
| 26 | L_3 | L | 3 | 0.730 | 0.718 | 0.020 | 0.048 | 0.020 | 0.007 | 0.000 | 0.154 | 0.037 | 2939.264 | 57.977 | 1.54E-13 | 6 |
| 27 | LQ_3 | LQ | 3 | 0.738 | 0.727 | 0.017 | 0.046 | 0.018 | 0.007 | 0.000 | 0.147 | 0.032 | 2935.452 | 54.164 | 1.04E-12 | 7 |
| 28 | LQH_3 | LQH | 3 | 0.786 | 0.764 | 0.015 | 0.047 | 0.015 | 0.019 | 0.001 | 0.154 | 0.037 | 2904.237 | 22.950 | 6.22E-06 | 10 |
| 29 | LQHP_3 | LQHP | 3 | 0.805 | 0.758 | 0.016 | 0.057 | 0.017 | 0.012 | 0.001 | 0.171 | 0.033 | 2906.165 | 24.877 | 2.37E-06 | 13 |
| 30 | LQHPT_3 | LQHPT | 3 | 0.807 | 0.760 | 0.013 | 0.056 | 0.017 | 0.012 | 0.001 | 0.196 | 0.024 | 2907.671 | 26.384 | 1.12E-06 | 14 |
| 31 | L_3.5 | L | 3.5 | 0.729 | 0.719 | 0.021 | 0.048 | 0.020 | 0.007 | 0.000 | 0.154 | 0.037 | 2940.459 | 59.171 | 8.48E-14 | 6 |
| 32 | LQ_3.5 | LQ | 3.5 | 0.738 | 0.728 | 0.018 | 0.045 | 0.018 | 0.007 | 0.000 | 0.147 | 0.032 | 2936.484 | 55.196 | 6.19E-13 | 7 |
| 33 | LQH_3.5 | LQH | 3.5 | 0.782 | 0.761 | 0.017 | 0.046 | 0.016 | 0.007 | 0.000 | 0.154 | 0.037 | 2906.474 | 25.186 | 2.03E-06 | 8 |
| 34 | LQHP_3.5 | LQHP | 3.5 | 0.795 | 0.748 | 0.018 | 0.060 | 0.020 | 0.000 | 0.000 | 0.167 | 0.035 | 2913.352 | 32.064 | 6.52E-08 | 13 |
| 35 | LQHPT_3.5 | LQHPT | 3.5 | 0.798 | 0.748 | 0.018 | 0.060 | 0.020 | 0.000 | 0.000 | 0.167 | 0.035 | 2915.057 | 33.769 | 2.78E-08 | 14 |
| 36 | L_4 | L | 4 | 0.728 | 0.719 | 0.022 | 0.049 | 0.020 | 0.007 | 0.000 | 0.154 | 0.037 | 2941.713 | 60.426 | 4.53E-14 | 6 |
| 37 | LQ_4 | LQ | 4 | 0.737 | 0.728 | 0.019 | 0.046 | 0.019 | 0.007 | 0.000 | 0.147 | 0.032 | 2937.558 | 56.271 | 3.62E-13 | 7 |
| 38 | LQH_4 | LQH | 4 | 0.779 | 0.757 | 0.018 | 0.048 | 0.017 | 0.007 | 0.000 | 0.154 | 0.037 | 2910.510 | 29.222 | 2.70E-07 | 8 |
| 39 | LQHP_4 | LQHP | 4 | 0.785 | 0.741 | 0.020 | 0.061 | 0.022 | 0.000 | 0.000 | 0.154 | 0.037 | 2917.776 | 36.488 | 7.14E-09 | 12 |
| 40 | LQHPT_4 | LQHPT | 4 | 0.787 | 0.741 | 0.020 | 0.061 | 0.022 | 0.000 | 0.000 | 0.154 | 0.037 | 2922.173 | 40.885 | 7.93E-10 | 14 |
| **Sesuvium.portulacastrum** | settings | features | rm | train.AUC | avg.test.AUC | var.test.AUC | avg.diff.AUC | var.diff.AUC | avg.test.orMTP | var.test.orMTP | avg.test.or10pct | var.test.or10pct | AICc | delta.AICc | w.AIC | parameters |
| 1 | L_0.5 | L | 0.5 | 0.704 | 0.671 | 0.014 | 0.066 | 0.013 | 0.011 | 0.001 | 0.193 | 0.035 | 2195.202 | 53.967 | 1.38E-12 | 6 |
| 2 | LQ_0.5 | LQ | 0.5 | 0.731 | 0.698 | 0.024 | 0.076 | 0.022 | 0.011 | 0.001 | 0.162 | 0.013 | 2193.483 | 52.248 | 3.27E-12 | 10 |
| 3 | LQH_0.5 | LQH | 0.5 | 0.890 | 0.812 | 0.019 | 0.083 | 0.022 | 0.049 | 0.003 | 0.259 | 0.044 | 2196.067 | 54.832 | 8.98E-13 | 33 |
| 4 | LQHP_0.5 | LQHP | 0.5 | 0.921 | 0.832 | 0.018 | 0.086 | 0.022 | 0.077 | 0.006 | 0.301 | 0.052 | 2141.235 | 0.000 | 0.7240463 | 32 |
| 5 | LQHPT_0.5 | LQHPT | 0.5 | 0.942 | 0.803 | 0.007 | 0.132 | 0.008 | 0.113 | 0.010 | 0.447 | 0.011 | 2255.503 | 114.268 | 1.11E-25 | 54 |
| 6 | L_1 | L | 1 | 0.704 | 0.668 | 0.013 | 0.065 | 0.013 | 0.011 | 0.001 | 0.175 | 0.026 | 2195.880 | 54.645 | 9.86E-13 | 6 |
| 7 | LQ_1 | LQ | 1 | 0.733 | 0.694 | 0.022 | 0.077 | 0.021 | 0.011 | 0.001 | 0.190 | 0.021 | 2194.510 | 53.276 | 1.95E-12 | 10 |
| 8 | LQH_1 | LQH | 1 | 0.842 | 0.773 | 0.026 | 0.080 | 0.030 | 0.022 | 0.001 | 0.186 | 0.045 | 2175.283 | 34.049 | 2.93E-08 | 20 |
| 9 | LQHP_1 | LQHP | 1 | 0.890 | 0.788 | 0.022 | 0.082 | 0.028 | 0.021 | 0.002 | 0.264 | 0.068 | 2145.383 | 4.149 | 0.090976 | 25 |
| 10 | LQHPT_1 | LQHPT | 1 | 0.903 | 0.780 | 0.019 | 0.094 | 0.025 | 0.021 | 0.002 | 0.334 | 0.060 | 2159.802 | 18.568 | 6.73E-05 | 31 |
| 11 | L_1.5 | L | 1.5 | 0.703 | 0.666 | 0.013 | 0.065 | 0.013 | 0.011 | 0.001 | 0.185 | 0.029 | 2196.556 | 55.321 | 7.03E-13 | 6 |
| 12 | LQ_1.5 | LQ | 1.5 | 0.729 | 0.691 | 0.019 | 0.075 | 0.019 | 0.011 | 0.001 | 0.211 | 0.028 | 2196.017 | 54.782 | 9.20E-13 | 10 |
| 13 | LQH_1.5 | LQH | 1.5 | 0.808 | 0.751 | 0.022 | 0.075 | 0.027 | 0.022 | 0.001 | 0.187 | 0.056 | 2174.231 | 32.997 | 4.95E-08 | 13 |
| 14 | LQHP_1.5 | LQHP | 1.5 | 0.861 | 0.759 | 0.025 | 0.087 | 0.029 | 0.032 | 0.002 | 0.264 | 0.068 | 2144.217 | 2.983 | 0.1629623 | 18 |
| 15 | LQHPT_1.5 | LQHPT | 1.5 | 0.868 | 0.757 | 0.024 | 0.089 | 0.029 | 0.032 | 0.002 | 0.264 | 0.068 | 2148.548 | 7.314 | 0.0186909 | 20 |
| 16 | L_2 | L | 2 | 0.703 | 0.664 | 0.012 | 0.065 | 0.013 | 0.011 | 0.001 | 0.178 | 0.033 | 2197.328 | 56.094 | 4.78E-13 | 6 |
| 17 | LQ_2 | LQ | 2 | 0.728 | 0.689 | 0.017 | 0.072 | 0.016 | 0.011 | 0.001 | 0.172 | 0.020 | 2200.441 | 59.207 | 1.01E-13 | 11 |
| 18 | LQH_2 | LQH | 2 | 0.800 | 0.738 | 0.023 | 0.079 | 0.026 | 0.022 | 0.001 | 0.204 | 0.035 | 2177.520 | 36.286 | 9.56E-09 | 12 |
| 19 | LQHP_2 | LQHP | 2 | 0.845 | 0.737 | 0.023 | 0.088 | 0.028 | 0.022 | 0.001 | 0.245 | 0.057 | 2152.084 | 10.849 | 0.0031908 | 16 |
| 20 | LQHPT_2 | LQHPT | 2 | 0.849 | 0.737 | 0.023 | 0.088 | 0.028 | 0.022 | 0.001 | 0.245 | 0.057 | 2160.766 | 19.531 | 4.16E-05 | 19 |
| 21 | L_2.5 | L | 2.5 | 0.704 | 0.663 | 0.011 | 0.063 | 0.012 | 0.011 | 0.001 | 0.178 | 0.033 | 2198.145 | 56.911 | 3.18E-13 | 6 |
| 22 | LQ_2.5 | LQ | 2.5 | 0.726 | 0.686 | 0.015 | 0.069 | 0.015 | 0.011 | 0.001 | 0.182 | 0.023 | 2197.429 | 56.195 | 4.54E-13 | 9 |
| 23 | LQH_2.5 | LQH | 2.5 | 0.791 | 0.719 | 0.024 | 0.084 | 0.026 | 0.022 | 0.001 | 0.162 | 0.035 | 2183.727 | 42.493 | 4.29E-10 | 12 |
| 24 | LQHP_2.5 | LQHP | 2.5 | 0.832 | 0.721 | 0.021 | 0.086 | 0.026 | 0.022 | 0.001 | 0.192 | 0.027 | 2163.220 | 21.985 | 1.22E-05 | 16 |
| 25 | LQHPT_2.5 | LQHPT | 2.5 | 0.832 | 0.722 | 0.022 | 0.086 | 0.026 | 0.022 | 0.001 | 0.192 | 0.027 | 2163.226 | 21.991 | 1.21E-05 | 16 |
| 26 | L_3 | L | 3 | 0.703 | 0.661 | 0.010 | 0.063 | 0.012 | 0.011 | 0.001 | 0.193 | 0.026 | 2198.996 | 57.762 | 2.07E-13 | 6 |
| 27 | LQ_3 | LQ | 3 | 0.722 | 0.685 | 0.014 | 0.066 | 0.013 | 0.011 | 0.001 | 0.144 | 0.011 | 2199.513 | 58.278 | 1.60E-13 | 9 |
| 28 | LQH_3 | LQH | 3 | 0.776 | 0.702 | 0.021 | 0.085 | 0.026 | 0.022 | 0.001 | 0.166 | 0.023 | 2193.480 | 52.245 | 3.27E-12 | 13 |
| 29 | LQHP_3 | LQHP | 3 | 0.822 | 0.701 | 0.017 | 0.084 | 0.025 | 0.022 | 0.001 | 0.155 | 0.015 | 2173.852 | 32.617 | 5.98E-08 | 17 |
| 30 | LQHPT_3 | LQHPT | 3 | 0.822 | 0.701 | 0.017 | 0.084 | 0.025 | 0.022 | 0.001 | 0.155 | 0.015 | 2173.852 | 32.617 | 5.98E-08 | 17 |
| 31 | L_3.5 | L | 3.5 | 0.703 | 0.660 | 0.010 | 0.062 | 0.012 | 0.011 | 0.001 | 0.193 | 0.026 | 2199.897 | 58.662 | 1.32E-13 | 6 |
| 32 | LQ_3.5 | LQ | 3.5 | 0.719 | 0.686 | 0.012 | 0.062 | 0.011 | 0.011 | 0.001 | 0.144 | 0.011 | 2201.745 | 60.510 | 5.25E-14 | 9 |
| 33 | LQH_3.5 | LQH | 3.5 | 0.757 | 0.694 | 0.018 | 0.083 | 0.024 | 0.022 | 0.001 | 0.175 | 0.024 | 2199.926 | 58.691 | 1.30E-13 | 13 |
| 34 | LQHP_3.5 | LQHP | 3.5 | 0.813 | 0.692 | 0.016 | 0.082 | 0.022 | 0.022 | 0.001 | 0.202 | 0.030 | 2171.872 | 30.637 | 1.61E-07 | 14 |
| 35 | LQHPT_3.5 | LQHPT | 3.5 | 0.813 | 0.692 | 0.016 | 0.082 | 0.022 | 0.022 | 0.001 | 0.202 | 0.030 | 2171.872 | 30.637 | 1.61E-07 | 14 |
| 36 | L_4 | L | 4 | 0.703 | 0.660 | 0.010 | 0.061 | 0.011 | 0.011 | 0.001 | 0.178 | 0.033 | 2200.838 | 59.603 | 8.26E-14 | 6 |
| 37 | LQ_4 | LQ | 4 | 0.718 | 0.686 | 0.012 | 0.060 | 0.011 | 0.011 | 0.001 | 0.144 | 0.011 | 2200.790 | 59.556 | 8.46E-14 | 8 |
| 38 | LQH_4 | LQH | 4 | 0.735 | 0.689 | 0.016 | 0.078 | 0.020 | 0.022 | 0.001 | 0.175 | 0.024 | 2204.683 | 63.449 | 1.21E-14 | 12 |
| 39 | LQHP_4 | LQHP | 4 | 0.807 | 0.688 | 0.015 | 0.075 | 0.018 | 0.022 | 0.001 | 0.153 | 0.014 | 2177.621 | 36.387 | 9.09E-09 | 14 |
| 40 | LQHPT_4 | LQHPT | 4 | 0.807 | 0.688 | 0.015 | 0.075 | 0.018 | 0.022 | 0.001 | 0.153 | 0.014 | 2177.621 | 36.387 | 9.09E-09 | 14 |
| **Spartina.alterniflora** | settings | features | rm | train.AUC | avg.test.AUC | var.test.AUC | avg.diff.AUC | var.diff.AUC | avg.test.orMTP | var.test.orMTP | avg.test.or10pct | var.test.or10pct | AICc | delta.AICc | w.AIC | parameters |
| 1 | L_0.5 | L | 0.5 | 0.819 | 0.801 | 0.006 | 0.034 | 0.003 | 0.022 | 0.002 | 0.135 | 0.013 | 1471.255 | 44.507 | 1.60E-10 | 6 |
| 2 | LQ_0.5 | LQ | 0.5 | 0.878 | 0.867 | 0.001 | 0.015 | 0.001 | 0.025 | 0.003 | 0.126 | 0.023 | 1429.532 | 2.784 | 0.1831579 | 8 |
| 3 | LQH_0.5 | LQH | 0.5 | 0.950 | 0.917 | 0.001 | 0.031 | 0.001 | 0.077 | 0.008 | 0.199 | 0.005 | 1456.122 | 29.373 | 3.08E-07 | 28 |
| 4 | LQHP_0.5 | LQHP | 0.5 | 0.954 | 0.909 | 0.001 | 0.041 | 0.001 | 0.077 | 0.008 | 0.235 | 0.013 | 1437.188 | 10.440 | 0.0039835 | 25 |
| 5 | LQHPT_0.5 | LQHPT | 0.5 | 0.961 | 0.915 | 0.000 | 0.044 | 0.000 | 0.140 | 0.009 | 0.317 | 0.009 | 1483.993 | 57.244 | 2.73E-13 | 35 |
| 6 | L_1 | L | 1 | 0.818 | 0.799 | 0.007 | 0.035 | 0.004 | 0.022 | 0.002 | 0.113 | 0.012 | 1472.263 | 45.515 | 9.64E-11 | 6 |
| 7 | LQ_1 | LQ | 1 | 0.873 | 0.848 | 0.003 | 0.024 | 0.001 | 0.047 | 0.003 | 0.105 | 0.007 | 1436.253 | 9.505 | 0.0063576 | 7 |
| 8 | LQH_1 | LQH | 1 | 0.929 | 0.904 | 0.002 | 0.028 | 0.002 | 0.077 | 0.008 | 0.167 | 0.010 | 1426.748 | 0.000 | 0.7366454 | 15 |
| 9 | LQHP_1 | LQHP | 1 | 0.930 | 0.905 | 0.001 | 0.026 | 0.001 | 0.077 | 0.008 | 0.167 | 0.010 | 1436.042 | 9.294 | 0.007065 | 17 |
| 10 | LQHPT_1 | LQHPT | 1 | 0.939 | 0.906 | 0.001 | 0.030 | 0.001 | 0.093 | 0.012 | 0.229 | 0.010 | 1432.131 | 5.383 | 0.0499248 | 19 |
| 11 | L_1.5 | L | 1.5 | 0.816 | 0.799 | 0.007 | 0.035 | 0.004 | 0.022 | 0.002 | 0.113 | 0.012 | 1470.963 | 44.215 | 1.85E-10 | 5 |
| 12 | LQ_1.5 | LQ | 1.5 | 0.869 | 0.821 | 0.007 | 0.033 | 0.003 | 0.022 | 0.002 | 0.138 | 0.014 | 1447.248 | 20.500 | 2.60E-05 | 7 |
| 13 | LQH_1.5 | LQH | 1.5 | 0.920 | 0.898 | 0.003 | 0.028 | 0.002 | 0.043 | 0.008 | 0.127 | 0.008 | 1440.540 | 13.792 | 0.0007454 | 16 |
| 14 | LQHP_1.5 | LQHP | 1.5 | 0.921 | 0.898 | 0.002 | 0.025 | 0.002 | 0.077 | 0.008 | 0.167 | 0.010 | 1435.625 | 8.876 | 0.008705 | 14 |
| 15 | LQHPT_1.5 | LQHPT | 1.5 | 0.923 | 0.897 | 0.001 | 0.028 | 0.002 | 0.043 | 0.008 | 0.201 | 0.011 | 1442.985 | 16.237 | 0.0002195 | 17 |
| 16 | L_2 | L | 2 | 0.816 | 0.799 | 0.007 | 0.035 | 0.004 | 0.022 | 0.002 | 0.113 | 0.012 | 1471.836 | 45.087 | 1.19E-10 | 5 |
| 17 | LQ_2 | LQ | 2 | 0.851 | 0.814 | 0.007 | 0.035 | 0.004 | 0.022 | 0.002 | 0.113 | 0.012 | 1459.330 | 32.582 | 6.20E-08 | 7 |
| 18 | LQH_2 | LQH | 2 | 0.911 | 0.888 | 0.003 | 0.029 | 0.002 | 0.077 | 0.008 | 0.127 | 0.008 | 1439.419 | 12.671 | 0.0013056 | 12 |
| 19 | LQHP_2 | LQHP | 2 | 0.913 | 0.887 | 0.002 | 0.026 | 0.002 | 0.077 | 0.008 | 0.142 | 0.004 | 1440.479 | 13.731 | 0.0007684 | 12 |
| 20 | LQHPT_2 | LQHPT | 2 | 0.913 | 0.886 | 0.002 | 0.029 | 0.002 | 0.043 | 0.008 | 0.159 | 0.004 | 1440.479 | 13.731 | 0.0007684 | 12 |
| 21 | L_2.5 | L | 2.5 | 0.815 | 0.798 | 0.007 | 0.036 | 0.004 | 0.022 | 0.002 | 0.113 | 0.012 | 1472.747 | 45.999 | 7.56E-11 | 5 |
| 22 | LQ_2.5 | LQ | 2.5 | 0.827 | 0.813 | 0.008 | 0.036 | 0.004 | 0.022 | 0.002 | 0.113 | 0.012 | 1468.503 | 41.755 | 6.31E-10 | 6 |
| 23 | LQH_2.5 | LQH | 2.5 | 0.902 | 0.869 | 0.004 | 0.035 | 0.002 | 0.102 | 0.006 | 0.149 | 0.013 | 1442.856 | 16.108 | 0.0002342 | 10 |
| 24 | LQHP_2.5 | LQHP | 2.5 | 0.904 | 0.866 | 0.002 | 0.030 | 0.001 | 0.055 | 0.004 | 0.161 | 0.016 | 1446.125 | 19.377 | 4.57E-05 | 10 |
| 25 | LQHPT_2.5 | LQHPT | 2.5 | 0.904 | 0.864 | 0.002 | 0.033 | 0.001 | 0.055 | 0.004 | 0.161 | 0.016 | 1446.125 | 19.377 | 4.57E-05 | 10 |
| 26 | L_3 | L | 3 | 0.814 | 0.797 | 0.008 | 0.037 | 0.005 | 0.022 | 0.002 | 0.113 | 0.012 | 1473.680 | 46.932 | 4.74E-11 | 5 |
| 27 | LQ_3 | LQ | 3 | 0.826 | 0.812 | 0.008 | 0.036 | 0.004 | 0.022 | 0.002 | 0.145 | 0.007 | 1470.590 | 43.842 | 2.22E-10 | 6 |
| 28 | LQH_3 | LQH | 3 | 0.888 | 0.836 | 0.009 | 0.043 | 0.003 | 0.000 | 0.000 | 0.157 | 0.017 | 1453.099 | 26.351 | 1.40E-06 | 10 |
| 29 | LQHP_3 | LQHP | 3 | 0.895 | 0.818 | 0.009 | 0.043 | 0.003 | 0.022 | 0.002 | 0.135 | 0.013 | 1456.776 | 30.027 | 2.22E-07 | 10 |
| 30 | LQHPT_3 | LQHPT | 3 | 0.895 | 0.822 | 0.007 | 0.042 | 0.002 | 0.022 | 0.002 | 0.135 | 0.013 | 1456.776 | 30.027 | 2.22E-07 | 10 |
| 31 | L_3.5 | L | 3.5 | 0.814 | 0.797 | 0.008 | 0.037 | 0.005 | 0.022 | 0.002 | 0.113 | 0.012 | 1474.648 | 47.900 | 2.92E-11 | 5 |
| 32 | LQ_3.5 | LQ | 3.5 | 0.826 | 0.810 | 0.009 | 0.037 | 0.005 | 0.022 | 0.002 | 0.120 | 0.012 | 1469.366 | 42.617 | 4.10E-10 | 5 |
| 33 | LQH_3.5 | LQH | 3.5 | 0.869 | 0.821 | 0.008 | 0.038 | 0.004 | 0.022 | 0.002 | 0.145 | 0.007 | 1462.059 | 35.311 | 1.58E-08 | 9 |
| 34 | LQHP_3.5 | LQHP | 3.5 | 0.878 | 0.804 | 0.006 | 0.037 | 0.003 | 0.022 | 0.002 | 0.110 | 0.018 | 1463.246 | 36.498 | 8.75E-09 | 8 |
| 35 | LQHPT_3.5 | LQHPT | 3.5 | 0.878 | 0.804 | 0.006 | 0.037 | 0.003 | 0.022 | 0.002 | 0.110 | 0.018 | 1463.246 | 36.498 | 8.75E-09 | 8 |
| 36 | L_4 | L | 4 | 0.814 | 0.797 | 0.009 | 0.038 | 0.005 | 0.022 | 0.002 | 0.113 | 0.012 | 1475.644 | 48.895 | 1.78E-11 | 5 |
| 37 | LQ_4 | LQ | 4 | 0.825 | 0.808 | 0.009 | 0.038 | 0.005 | 0.022 | 0.002 | 0.120 | 0.012 | 1470.538 | 43.790 | 2.28E-10 | 5 |
| 38 | LQH_4 | LQH | 4 | 0.835 | 0.816 | 0.009 | 0.036 | 0.004 | 0.022 | 0.002 | 0.120 | 0.012 | 1475.550 | 48.802 | 1.86E-11 | 9 |
| 39 | LQHP_4 | LQHP | 4 | 0.850 | 0.799 | 0.007 | 0.032 | 0.004 | 0.022 | 0.002 | 0.110 | 0.018 | 1473.977 | 47.228 | 4.09E-11 | 8 |
| 40 | LQHPT_4 | LQHPT | 4 | 0.850 | 0.799 | 0.007 | 0.032 | 0.004 | 0.022 | 0.002 | 0.110 | 0.018 | 1473.977 | 47.228 | 4.09E-11 | 8 |
| **Sporobolus.virginicus** | settings | features | rm | train.AUC | avg.test.AUC | var.test.AUC | avg.diff.AUC | var.diff.AUC | avg.test.orMTP | var.test.orMTP | avg.test.or10pct | var.test.or10pct | AICc | delta.AICc | w.AIC | parameters |
| 1 | L_0.5 | L | 0.5 | 0.657 | 0.628 | 0.009 | 0.052 | 0.011 | 0.008 | 0.000 | 0.145 | 0.017 | 2924.016 | 93.893 | 3.57E-21 | 6 |
| 2 | LQ_0.5 | LQ | 0.5 | 0.735 | 0.687 | 0.002 | 0.057 | 0.003 | 0.015 | 0.001 | 0.153 | 0.001 | 2894.650 | 64.527 | 8.49E-15 | 11 |
| 3 | LQH_0.5 | LQH | 0.5 | 0.867 | 0.783 | 0.006 | 0.092 | 0.009 | 0.051 | 0.003 | 0.266 | 0.020 | 2880.329 | 50.207 | 1.09E-11 | 37 |
| 4 | LQHP_0.5 | LQHP | 0.5 | 0.879 | 0.785 | 0.005 | 0.100 | 0.007 | 0.064 | 0.005 | 0.276 | 0.003 | 2861.290 | 31.168 | 1.49E-07 | 37 |
| 5 | LQHPT_0.5 | LQHPT | 0.5 | 0.911 | 0.799 | 0.002 | 0.110 | 0.003 | 0.124 | 0.004 | 0.315 | 0.003 | 2892.244 | 62.122 | 2.83E-14 | 49 |
| 6 | L_1 | L | 1 | 0.658 | 0.629 | 0.009 | 0.052 | 0.012 | 0.008 | 0.000 | 0.110 | 0.003 | 2924.634 | 94.511 | 2.62E-21 | 6 |
| 7 | LQ_1 | LQ | 1 | 0.730 | 0.683 | 0.003 | 0.056 | 0.005 | 0.015 | 0.001 | 0.161 | 0.002 | 2895.618 | 65.495 | 5.23E-15 | 9 |
| 8 | LQH_1 | LQH | 1 | 0.844 | 0.785 | 0.004 | 0.065 | 0.006 | 0.035 | 0.001 | 0.200 | 0.002 | 2852.783 | 22.661 | 1.05E-05 | 20 |
| 9 | LQHP_1 | LQHP | 1 | 0.862 | 0.790 | 0.008 | 0.076 | 0.011 | 0.042 | 0.002 | 0.222 | 0.019 | 2834.074 | 3.952 | 0.1209631 | 22 |
| 10 | LQHPT_1 | LQHPT | 1 | 0.869 | 0.786 | 0.007 | 0.086 | 0.009 | 0.051 | 0.002 | 0.230 | 0.017 | 2830.122 | 0.000 | 0.8725927 | 23 |
| 11 | L_1.5 | L | 1.5 | 0.658 | 0.629 | 0.010 | 0.052 | 0.012 | 0.008 | 0.000 | 0.110 | 0.003 | 2925.267 | 95.145 | 1.91E-21 | 6 |
| 12 | LQ_1.5 | LQ | 1.5 | 0.727 | 0.679 | 0.003 | 0.053 | 0.006 | 0.015 | 0.001 | 0.194 | 0.001 | 2900.382 | 70.259 | 4.83E-16 | 9 |
| 13 | LQH_1.5 | LQH | 1.5 | 0.833 | 0.774 | 0.003 | 0.061 | 0.006 | 0.043 | 0.000 | 0.166 | 0.010 | 2858.199 | 28.077 | 6.98E-07 | 16 |
| 14 | LQHP_1.5 | LQHP | 1.5 | 0.853 | 0.783 | 0.011 | 0.071 | 0.014 | 0.037 | 0.002 | 0.213 | 0.021 | 2846.014 | 15.892 | 0.000309 | 21 |
| 15 | LQHPT_1.5 | LQHPT | 1.5 | 0.858 | 0.782 | 0.010 | 0.073 | 0.014 | 0.030 | 0.002 | 0.213 | 0.021 | 2846.941 | 16.819 | 0.0001944 | 23 |
| 16 | L_2 | L | 2 | 0.659 | 0.629 | 0.010 | 0.052 | 0.012 | 0.008 | 0.000 | 0.110 | 0.003 | 2926.003 | 95.881 | 1.32E-21 | 6 |
| 17 | LQ_2 | LQ | 2 | 0.719 | 0.673 | 0.005 | 0.051 | 0.008 | 0.015 | 0.001 | 0.175 | 0.007 | 2905.794 | 75.672 | 3.23E-17 | 9 |
| 18 | LQH_2 | LQH | 2 | 0.824 | 0.758 | 0.003 | 0.059 | 0.006 | 0.050 | 0.000 | 0.132 | 0.008 | 2868.288 | 38.165 | 4.50E-09 | 15 |
| 19 | LQHP_2 | LQHP | 2 | 0.840 | 0.779 | 0.013 | 0.069 | 0.016 | 0.037 | 0.002 | 0.197 | 0.013 | 2841.184 | 11.061 | 0.0034584 | 14 |
| 20 | LQHPT_2 | LQHPT | 2 | 0.845 | 0.779 | 0.012 | 0.071 | 0.015 | 0.037 | 0.002 | 0.197 | 0.013 | 2842.251 | 12.129 | 0.0020283 | 16 |
| 21 | L_2.5 | L | 2.5 | 0.658 | 0.630 | 0.010 | 0.052 | 0.012 | 0.008 | 0.000 | 0.110 | 0.003 | 2926.798 | 96.675 | 8.87E-22 | 6 |
| 22 | LQ_2.5 | LQ | 2.5 | 0.712 | 0.672 | 0.006 | 0.049 | 0.009 | 0.008 | 0.000 | 0.159 | 0.005 | 2911.879 | 81.756 | 1.54E-18 | 9 |
| 23 | LQH_2.5 | LQH | 2.5 | 0.808 | 0.739 | 0.006 | 0.057 | 0.008 | 0.018 | 0.000 | 0.139 | 0.009 | 2880.731 | 50.608 | 8.94E-12 | 15 |
| 24 | LQHP_2.5 | LQHP | 2.5 | 0.838 | 0.765 | 0.015 | 0.075 | 0.017 | 0.024 | 0.002 | 0.197 | 0.013 | 2846.716 | 16.594 | 0.0002176 | 13 |
| 25 | LQHPT_2.5 | LQHPT | 2.5 | 0.839 | 0.766 | 0.015 | 0.077 | 0.017 | 0.024 | 0.002 | 0.197 | 0.013 | 2846.693 | 16.570 | 0.0002201 | 14 |
| 26 | L_3 | L | 3 | 0.659 | 0.629 | 0.010 | 0.052 | 0.013 | 0.008 | 0.000 | 0.110 | 0.003 | 2925.243 | 95.121 | 1.93E-21 | 5 |
| 27 | LQ_3 | LQ | 3 | 0.704 | 0.671 | 0.007 | 0.047 | 0.009 | 0.008 | 0.000 | 0.134 | 0.005 | 2914.468 | 84.346 | 4.22E-19 | 8 |
| 28 | LQH_3 | LQH | 3 | 0.791 | 0.717 | 0.009 | 0.061 | 0.010 | 0.005 | 0.000 | 0.121 | 0.004 | 2888.029 | 57.906 | 2.33E-13 | 13 |
| 29 | LQHP_3 | LQHP | 3 | 0.832 | 0.753 | 0.016 | 0.077 | 0.017 | 0.024 | 0.002 | 0.183 | 0.010 | 2855.018 | 24.896 | 3.43E-06 | 13 |
| 30 | LQHPT_3 | LQHPT | 3 | 0.833 | 0.755 | 0.016 | 0.078 | 0.017 | 0.024 | 0.002 | 0.183 | 0.010 | 2856.329 | 26.207 | 1.78E-06 | 14 |
| 31 | L_3.5 | L | 3.5 | 0.659 | 0.628 | 0.011 | 0.053 | 0.013 | 0.008 | 0.000 | 0.102 | 0.003 | 2925.846 | 95.724 | 1.43E-21 | 5 |
| 32 | LQ_3.5 | LQ | 3.5 | 0.694 | 0.666 | 0.007 | 0.046 | 0.009 | 0.008 | 0.000 | 0.134 | 0.005 | 2918.479 | 88.357 | 5.68E-20 | 8 |
| 33 | LQH_3.5 | LQH | 3.5 | 0.772 | 0.698 | 0.010 | 0.060 | 0.010 | 0.000 | 0.000 | 0.142 | 0.005 | 2893.897 | 63.775 | 1.24E-14 | 11 |
| 34 | LQHP_3.5 | LQHP | 3.5 | 0.825 | 0.741 | 0.016 | 0.078 | 0.017 | 0.024 | 0.002 | 0.183 | 0.010 | 2866.241 | 36.119 | 1.25E-08 | 14 |
| 35 | LQHPT_3.5 | LQHPT | 3.5 | 0.825 | 0.742 | 0.016 | 0.079 | 0.017 | 0.024 | 0.002 | 0.183 | 0.010 | 2866.241 | 36.119 | 1.25E-08 | 14 |
| 36 | L_4 | L | 4 | 0.657 | 0.627 | 0.011 | 0.053 | 0.013 | 0.008 | 0.000 | 0.102 | 0.003 | 2926.487 | 96.364 | 1.04E-21 | 5 |
| 37 | LQ_4 | LQ | 4 | 0.689 | 0.664 | 0.007 | 0.042 | 0.008 | 0.008 | 0.000 | 0.134 | 0.005 | 2919.566 | 89.444 | 3.30E-20 | 7 |
| 38 | LQH_4 | LQH | 4 | 0.745 | 0.688 | 0.010 | 0.054 | 0.010 | 0.000 | 0.000 | 0.153 | 0.008 | 2903.131 | 73.008 | 1.22E-16 | 10 |
| 39 | LQHP_4 | LQHP | 4 | 0.816 | 0.725 | 0.015 | 0.081 | 0.015 | 0.024 | 0.002 | 0.172 | 0.012 | 2875.074 | 44.952 | 1.51E-10 | 14 |
| 40 | LQHPT_4 | LQHPT | 4 | 0.816 | 0.727 | 0.015 | 0.081 | 0.015 | 0.024 | 0.002 | 0.172 | 0.012 | 2875.074 | 44.952 | 1.51E-10 | 14 |
